# Whole-genome sequencing of the invasive golden apple snail *Pomacea canaliculata* from Asia reveals rapid expansion and adaptive evolution

**DOI:** 10.1093/gigascience/giae064

**Published:** 2024-09-23

**Authors:** Yan Lu, Fang Luo, An Zhou, Cun Yi, Hao Chen, Jian Li, Yunhai Guo, Yuxiang Xie, Wei Zhang, Datao Lin, Yaming Yang, Zhongdao Wu, Yi Zhang, Shuhua Xu, Wei Hu

**Affiliations:** State Key Laboratory of Genetic Engineering, Collaborative Innovation Center of Genetics and Development, School of Life Sciences, Fudan University, Shanghai 200438, China; Center for Evolutionary Biology, Ministry of Education Key Laboratory of Contemporary Anthropology, Fudan University, Shanghai 200438, China; State Key Laboratory of Genetic Engineering, Collaborative Innovation Center of Genetics and Development, School of Life Sciences, Fudan University, Shanghai 200438, China; State Key Laboratory of Genetic Engineering, Collaborative Innovation Center of Genetics and Development, School of Life Sciences, Fudan University, Shanghai 200438, China; Center for Evolutionary Biology, Ministry of Education Key Laboratory of Contemporary Anthropology, Fudan University, Shanghai 200438, China; State Key Laboratory of Genetic Engineering, Collaborative Innovation Center of Genetics and Development, School of Life Sciences, Fudan University, Shanghai 200438, China; Joint Research Laboratory of Genetics and Ecology on Parasite-host Interaction, Chinese Center for Disease Control and Prevention & Fudan University, Shanghai 200438, China; Key Laboratory of Computational Biology, Shanghai Institute of Nutrition and Health, University of Chinese Academy of Sciences, Chinese Academy of Sciences, Shanghai 200031, China; China Basic Medical College, Guangxi Traditional Chinese Medical University, Nanning 530005, China; National Institute of Parasitic Diseases, Chinese Center for Disease Control and Prevention (Chinese Center for Tropical Diseases Research); NHC Key Laboratory of Parasite and Vector Biology; WHO Collaborating Centre for Tropical Diseases; National Center for International Research on Tropical Diseases, Shanghai 200025, China; State Key Laboratory of Genetic Engineering, Collaborative Innovation Center of Genetics and Development, School of Life Sciences, Fudan University, Shanghai 200438, China; Joint Research Laboratory of Genetics and Ecology on Parasite-host Interaction, Chinese Center for Disease Control and Prevention & Fudan University, Shanghai 200438, China; State Key Laboratory of Genetic Engineering, Collaborative Innovation Center of Genetics and Development, School of Life Sciences, Fudan University, Shanghai 200438, China; Joint Research Laboratory of Genetics and Ecology on Parasite-host Interaction, Chinese Center for Disease Control and Prevention & Fudan University, Shanghai 200438, China; Zhongshan School of Medicine, Sun Yat-sen University, Guangzhou 510080, China; Yunnan Institute of Parasitic Diseases, Yunnan 665000, China; Zhongshan School of Medicine, Sun Yat-sen University, Guangzhou 510080, China; National Institute of Parasitic Diseases, Chinese Center for Disease Control and Prevention (Chinese Center for Tropical Diseases Research); NHC Key Laboratory of Parasite and Vector Biology; WHO Collaborating Centre for Tropical Diseases; National Center for International Research on Tropical Diseases, Shanghai 200025, China; State Key Laboratory of Genetic Engineering, Collaborative Innovation Center of Genetics and Development, School of Life Sciences, Fudan University, Shanghai 200438, China; Center for Evolutionary Biology, Ministry of Education Key Laboratory of Contemporary Anthropology, Fudan University, Shanghai 200438, China; State Key Laboratory of Genetic Engineering, Collaborative Innovation Center of Genetics and Development, School of Life Sciences, Fudan University, Shanghai 200438, China; Joint Research Laboratory of Genetics and Ecology on Parasite-host Interaction, Chinese Center for Disease Control and Prevention & Fudan University, Shanghai 200438, China; College of Life Sciences, Inner Mongolia University, Hohhot 010070, China

## Abstract

*Pomacea canaliculata*, an invasive species native to South America, is recognized for its broad geographic distribution and adaptability to a variety of ecological conditions. The details concerning the evolution and adaptation of *P. canaliculate* remain unclear due to a lack of whole-genome resequencing data. We examined 173 *P. canaliculata* genomes representing 17 geographic populations in East and Southeast Asia. Interestingly, *P. canaliculata* showed a higher level of genetic diversity than other mollusks, and our analysis suggested that the dispersal of *P. canaliculata* could have been driven by climate changes and human activities. Notably, we identified a set of genes associated with low temperature adaptation, including *Csde1*, a cold shock protein coding gene. Further RNA sequencing analysis and reverse transcription quantitative polymerase chain reaction experiments demonstrated the gene’s dynamic pattern and biological functions during cold exposure. Moreover, both positive selection and balancing selection are likely to have contributed to the rapid environmental adaptation of *P. canaliculata* populations. In particular, genes associated with energy metabolism and stress response were undergoing positive selection, while a large number of immune-related genes showed strong signatures of balancing selection. Our study has advanced our understanding of the evolution of *P. canaliculata* and has provided a valuable resource concerning an invasive species.

## Introduction

*Pomacea canaliculata* (NCBI:txid400727), commonly known as the golden apple snail, is a species of freshwater snail that originated in South America. As an invasive species, it was recently introduced to Asia as a commercial venture where it has become a serious pest of aquatic crops and rice [1]. *P. canaliculata* is listed among the 100 World’s Worst Invasive Species [2]. This species has become a widely distributed agricultural and environmental pest in southern China since its introduction in the 1980s [3]. *P. canaliculata* stands out among mollusks due to its wide geographic range and its ability to survive in a variety of ecological conditions. At present, rapid growth and expansion with high population densities have disturbed the local ecological balance and caused significant losses in many countries [4]. *P. canaliculata* is also a severe threat to human health in a number of areas, as it serves as a vector for a number of parasites that cause human diseases [5]. The snail acts as an intermediate host for the pathogen *Angiostrongylus cantonensis* that can infect humans and cause potentially fatal eosinophilic meningitis [6, 7].

*P. canaliculata* is thought to have experienced multiple origins based on the genetic study of mitochondrial cytochrome oxidase subunit 1 (COI) gene sequences [3, 8]. It has established natural populations in most of southern China but none in the northern China [3]. Geographical barriers are an important factor governing distribution patterns of native species. Human factors, however, were also likely to have been drivers of its invasion. *P. canaliculata* is highly adaptable, with tolerance to a variety of ecological environments as well as pathogen invasion. The recent successful range expansion of *P. canaliculata* provides a convenient system for studying the genetic diversity and the signature of rapid microevolution, particularly genetic mechanisms related to rapid local adaptation to novel environmental conditions in a short period of time. In addition, environmental factors such as temperature and pathogen load have influenced the distribution range of *P. canaliculata* [9]. Temperature may be a key environmental factor restricting the migration of *P. canaliculata* [10]. The ability to survive at low temperature constitutes a critical factor for successful range expansion of *P. canaliculata* in temperate East Asia as well as tropical Southeast Asia [11]. It has been suggested that low temperature in winter is a limiting factor in the geographic expansion and successful establishment of apple snail populations [12]. Previous study has shown that the expression of glycerol kinase (*GK*), heat shock protein 70 (*HSP70*), Na^+^/K^+^-ATPase (*NKA*), and glycerol-3-phosphate dehydrogenase (*GPDH*) genes is related to the cold hardiness of *P. canaliculate* [13, 14]. Transcriptome sequencing revealed that candidate cold-resistance genes were related to the glucose metabolism pathway. The lncRNA of *P. canaliculata* could participate in cold acclimation by regulating the expression of E3 ubiquitin protein ligase, 26S proteasome non-ATPase dependent regulation subunit, glutathione S-transferase, sodium/glucose cotransporter, and cytochrome *P450* [15]. However, the genetic mechanism of low temperature adaptation in *P. canaliculata* has not yet been investigated based on a large scale of whole-genome sequencing data, particularly at the population genetic level.

Despite the increasing biological and economic impacts of this invasive species, little is known about the evolutionary processes that underlie the geographic range expansion and adaptive evolution of invasiveness of *P. canaliculata*. In this study, we assembled a chromosomal-level reference genome from an adult female *P. canaliculata* that was collected from Shanghai, China, and we investigated the population structure, demographic history, genetic diversity, and local adaptation of *P. canaliculata* by sequencing and analyzing 173 whole genomes covering most of the current range of distribution in Asia. Our study revealed that *P. canaliculata* populations in Asia have undergone multiple episodes of rapid expansion that may have been driven by human factors. Furthermore, we identified a set of genes that may be involved in the adaptive invasion, particularly concerning adaptation to low temperatures. Additionally, balancing selection is likely to have contributed to the rapid environmental adaptation of *P. canaliculata* populations in Asia. Our findings provide insights into the genomic mechanisms of this invasive species that underlie the rapid local adaptation to novel ecological environments.

## Results

### A new reference genome for *P. canaliculata*

We assembled the *P. canaliculata* genome collected from Shanghai, China, by incorporating high coverage of PacBio CLR and high-throughput chromatin conformation capture (Hi-C) technologies. The PacBio reads were *de novo* assembled into contigs, followed by polishing with both PacBio and Illumina reads. This resulted in an assembly of 2,235 contigs with an N50 length of 1.16 Mb (Supplementary Table S1). A total of 434 million Hi-C read pairs were generated to scaffold the assembled contigs. Finally, we obtained a *P. canaliculata* reference genome (Pcan_SH) with scaffolds N50 of 31.4 Mb and genome length of 440.8 Mb. Notably, 432.4 Mb (98.11%) of sequence was anchored to 14 pseudochromosomes, which is similar to the published genome of *P. canaliculata* (Pcan_SZ, NCBI Accession: GCF_003073045) [16] (Supplementary Table S1, Supplementary Fig. S1). In total, 24,832 protein-coding genes were predicted, with over 91.81% of these genes being functionally annotated using the public databases (Supplementary Table S1).

Genome comparative analysis between the *P. canaliculata* genomes (Pcan_SH and Pcan_SZ) revealed intriguing insights. Hi-C interaction heatmaps for the Pcan_SH assembly displayed minimal interchromosomal interactions, contrasting with noticeable off-diagonal interactions in Pcan_SZ (Supplementary Fig. S2). Alignment of the genomes of Pcan_SH and Pcan_SZ showed good collinearity between the 2 reference genomes (Supplementary Fig. S3). Despite the high collinearity, we identified a total of 95 inversion, 1,242 translocations events. These chromosomal rearrangements were further supported by the high-density contacts in Hi-C heatmaps generated from Pcan_SZ Hi-C reads aligned to the Pcan_SZ genome, while no off-diagonal interactions were visible in Pcan_SH (Supplementary Fig. S4). These results suggested a more precise and accurate assembly process for Pcan_SH genome.

### Population structure and demographic history

**Figure 1:**
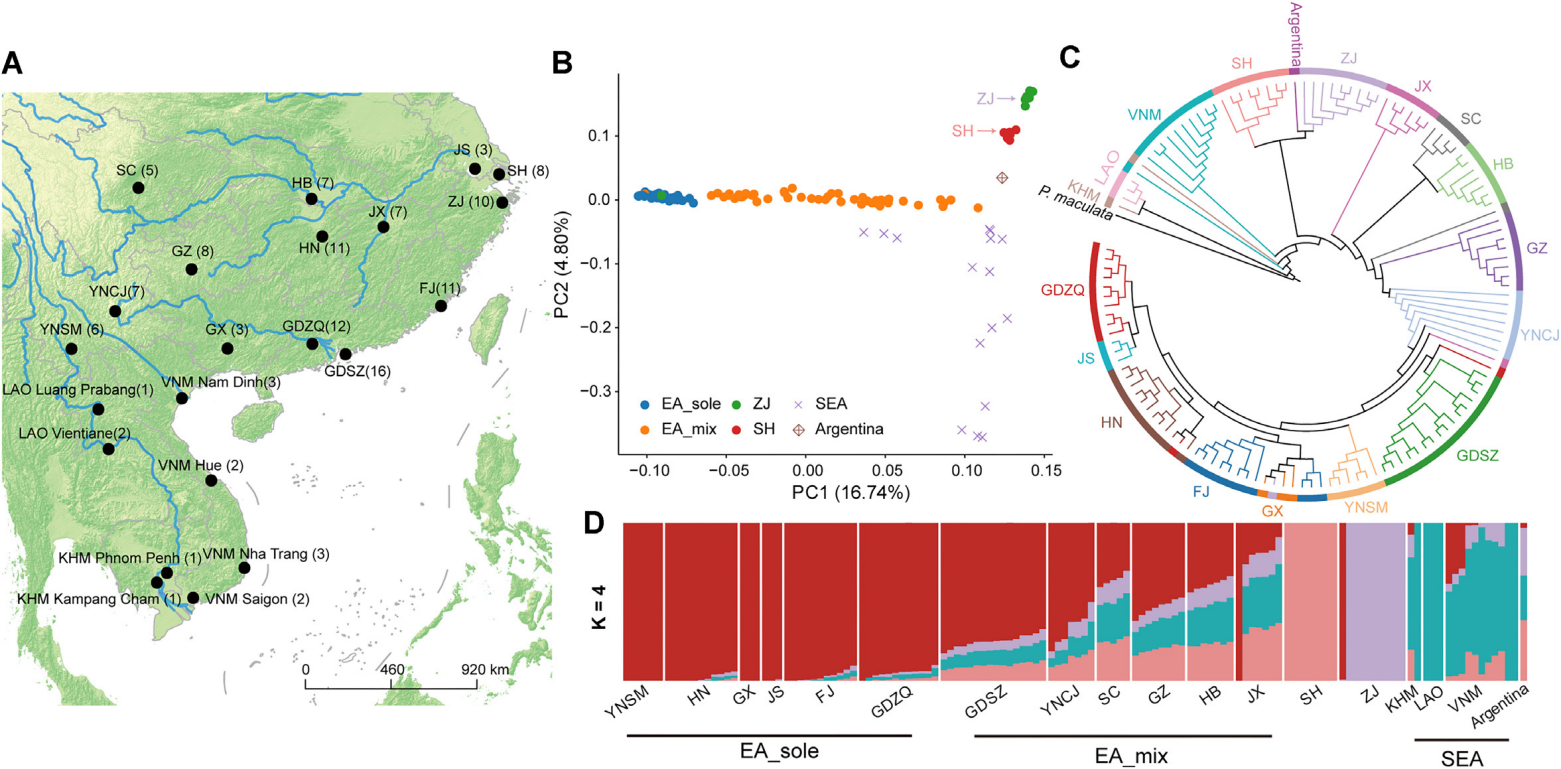
Sampling locations and population structure of *P. canaliculata*. (A) Geographic distribution of *P. canaliculata* samples. (B) PCA plot showing segregation of the *P. canaliculata* individuals. The proportions of the variance explained were 16.74% by PC1 and 4.80% by PC2. Each point is colored according to where the sample was collected. (C) ML phylogenetic tree of the *P. canaliculata* individuals with 1,000 nonparametric bootstrap replications. *Pomacea maculate* was selected as the outgroup. (D) Ancestry results from Admixture analysis under the best K = 4 model supported by an examination of cross-validation errors. Each color represents a different ancestry composition.

After quality control and filtration for genetic relatedness, 130 *P. canaliculata* genomes from East and Southeast Asia were retained for further analysis, with an additional genome from South America (Argentina). Using Pcan_SH as the reference genome, we identified a total of 13.55 million single nucleotide polymorphisms (SNPs) with an average 14.7× depth (Fig. 1A, Supplementary Fig. S5, Supplementary Tables S2–S3). Principal component analysis (PCA) revealed that East Asia (EA) and Southeast Asia (SEA) samples were divided into 2 distinct subclades in the 2-dimensional principal component (PC) plot, indicating a regional distribution pattern during the invasion. Samples from Shanghai (SH) and Zhejiang (ZJ) were grouped together in a subcluster of the EA populations; samples from Puer City, Yunnan (YNSM), Hunan (HN), Guangxi (GX), Jiangsu (JS), Fujian (FJ), and Zhaoqing City, Guangdong (GDZQ) were clustered together (EA_solo); and the remaining samples were scattered in a different cluster (EA_mix). Within the EA subclades, sampling locations did not discretely cluster along these PC axes; instead, we discovered that most EA populations, with the exception of SH and ZJ, maintained consistency with one another in PC2 but exhibited a continuous genetic structure in PC1 (Fig. 1B). Interestingly, there were no obvious subclusters reflected by most EA samples, and the resulting plots did not correspond to their geographic locations, possibly due to the multiple migrations and genetic interactions. Using the *P. maculate* genome as the outgroup, a maximum likelihood (ML) phylogenetic tree produced the same findings as the PCA. Samples from diverse geographical locations were classified into separate clades (Fig. 1C). Besides, SH and ZJ are near to the Argentina sample in the ML tree, suggesting a closer genetic affinity to the country of origin.

The population structure of *P. canaliculata* was further inferred by individual ancestry coefficients. We estimated 4 as the most likely number of ancestral populations based on the estimation of cross-validation (CV) error (Supplementary Fig. S6). Assuming K = 4, we found that the proportions of genetic components differed between EA and SEA populations. Interestingly, SH and ZJ samples shared their otherwise unique components, suggesting that there were limited genetic effects from other areas. East Asian and Southeast Asian components were present in 6 locations, Sichuan (SC), Shenzhen City, Guangdong (GDSZ), Yuxi City, Yunnan (YNCJ), Guizhou (GZ), Hubei (HB), and Jiangxi (JX), indicating multiple population interactions or invasions (EA_mix). Notably, the HN, GX, JS, FJ, and GDZQ populations (EA_solo) barely shared genetic components with the SEA population (Fig. 1D). These results corroborated previous studies using mitochondrial DNA (mtDNA) COI sequences [3] and supported the possibility of multiple invasions of Asia by showing varying degrees of migration and genetic interactions.

### Genomic diversity and genetic relationships

**Figure 2:**
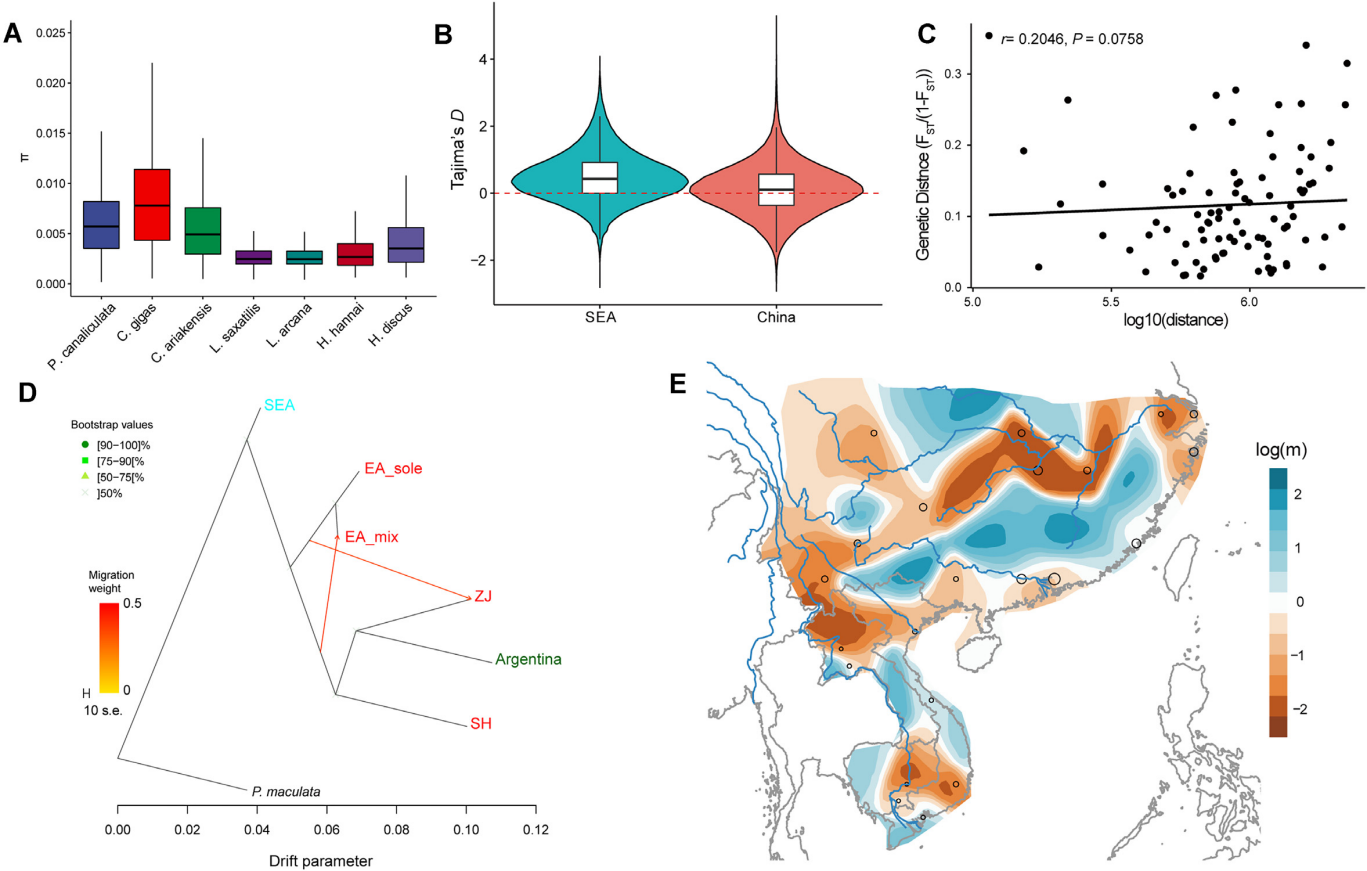
Genomic diversity and population migration among the *P. canaliculata* populations. (A) Estimates of genome-wide nucleotide diversity (π) in *P. canaliculata* and molluscan species with accessible whole-genome data are compared. (B) Tajima’s *D* calculated for each population. The violin plots show the kernel probability density of the data; the box represents the interquartile range, and the horizontal marker represents the median of the data. (C) Relationship between genetic distance (F_ST_/(1 − F_ST_)) and geographical distance for all sampled populations in East Asia (EA). The Spearman correlation coefficient (*ρ*) and the *P* value estimated using a Mantel test with 1,000 permutations are shown. (D) TreeMix-inferred population tree with 7 migration edges (*M* = 2). Migration events are indicated by arrows and are colored according to the migration weight. Bootstrap support is indicated for each of the nodes. (E) Effective migration (gene flow) surfaces estimated in EEMS for *P. canaliculata*. Color bars show the effective migration rate on a log_10_ scale relative to the average migration rate over the entire range. The darker blue indicates areas with stronger gene flow, whereas darker orange depicts areas with lower gene flow. The sizes of the black circles represent the number of sampled individuals in a given locality.

We estimated the genome-wide median nucleotide diversity (π) in populations of *P. canaliculata* and other molluscan species. The nucleotide diversity in *P. canaliculata* populations (range from 0.00427 to 0.0580; Supplementary Fig. S7) was comparable but significantly greater than previously published molluscan data, with the exception of another invasive species, *Crassostrea gigas* (Fig. 2A). Given the link between genetic diversity and ecological resilience [17], it stands to reason that *P. canaliculata* would have a higher level of genetic diversity than other mollusks. Due to the strong intrinsic link between linkage disequilibrium (LD) decay and genetic diversity, we then estimated the pairwise LD (*r*^2^) with all high-quality SNPs in *P. canaliculata* populations. As expected, the *r*^2^ value declined with the increasing physical distance between SNPs. The distance with *r*^2^ reaching half of its maximum value occurred at ∼30 bp across all snail populations (Supplementary Fig. S8). Genome-wide Tajima’s *D* estimates were positive for all populations, indicating an excess of intermediate-frequency polymorphism as a consequence of population contraction or balancing selection (Fig. 2B). Additionally, we found greater SNP differentiation among populations in the EA and SEA clades (range of F_ST_ = 0.0936–0.64382) than within the EA clades, with the exception of the SH and ZJ populations (range of F_ST_ = 0.0165–0.14833), suggesting a pattern of rapid radiation in the EA clades (Supplementary Table S4). It is noteworthy that no significant correlation was observed between genetic distance (F_ST_/(1 − F_ST_)) and geographical distance (great circle distance) in EA populations (*R* = 0.2046, *P* = 0.0758, Fig. 2C), indicating that human activity was involved during invasion events.

To explore the evolutionary relationships among populations and potential admixture events, we employed TreeMix and outgroup *f*3 to test for relatedness of different *P. canaliculata* populations. With the exceptions of ZJ and SH, all of the internal branch lengths in the EA clades were relatively short, and the TreeMix analysis detected frequent signals of gene flow among EA populations, a result that was consistent with populations that were rapidly spreading (Fig. 2D, Supplementary Figs. S9 and S10). Hybridization also likely occurred between SEA and EA populations. Taking *P. maculata* as an outgroup, the Argentina sample showed higher *f*3 values with EA populations than with SEA populations. ZJ and SH had a stronger affinity with Argentina. We calculated outgroup test in the form of *f*3 (VNM, X; *P. maculata*), when X belonged to EA populations, the target population Vietnam (VNM) had higher *f*3 values, indicating that it shared more genetic components with EA populations (Supplementary Fig. S11).

We then used estimated effective migrations surfaces (EEMS) analysis of EA populations to identify a distinct genetic barrier that runs roughly parallel to the Yangzi River Basin in China. It has been reported that *P. canaliculata* is predominantly found south of the Yangtze River Basin due to the ambient temperature. Founder events crossing the river and harsh environment during range expansion involved fewer individuals, leading to greater genetic drift between populations from northern and southern China. Moreover, the barriers in SEA populations coincided with 3 international boundaries (China–Laos, Laos–Vietnam, and Vietnam–Cambodia), further suggesting that human activity or cross-border trade possibly has been a major factor in the invasion of *P. canaliculata* (Fig. 2E and Supplementary Fig. S12).

### Genomic signatures of low-temperature adaptation

With the aid of human activity, populations of *P. canaliculata* have successfully invaded Asia within a relatively short time. The identification of genomic signatures that are consistently linked to invasion success has been made possible by these replicated invasion events. The most important factor driving the invasion of *P. canaliculata* is considered the environmental temperature, although many other variables, including the level of dissolved oxygen, the pH of the water, and soil moisture during dormancy, are associated with overwintering success [18]. Numerous studies have revealed that low temperature in winter is a limiting factor in the geographic expansion and successful establishment of apple snail populations [12, 19, 20]. Given the significant differences in temperature between East and Southeast Asia, as well as the different population structures inferred from the PCA, we used the BayPass software to conduct a genome-wide scan to identify genes involved in adaptation during invasion, with the Min Temperature of the Coldest Month (Bio06) selected as the primary environmental factor (see Materials and Methods). In total, 648 outlier SNPs with a Bayes factor (BF) greater than 20 were discovered, and 436 linked genes were annotated (Supplementary Fig. S13). We analyzed the Gene Ontology (GO) annotation of these genes (*P* < 0.05; see Materials and Methods) and found them to be clustered into 5 interacting networks that were linked to the functions of circadian sleep/wake cycle, associative chemosensory locomotory learning, circulatory circulation muscle contraction, negative action involved migration, and axonogenesis branching disc differentiation (Supplementary Fig. S14).

**Figure 3:**
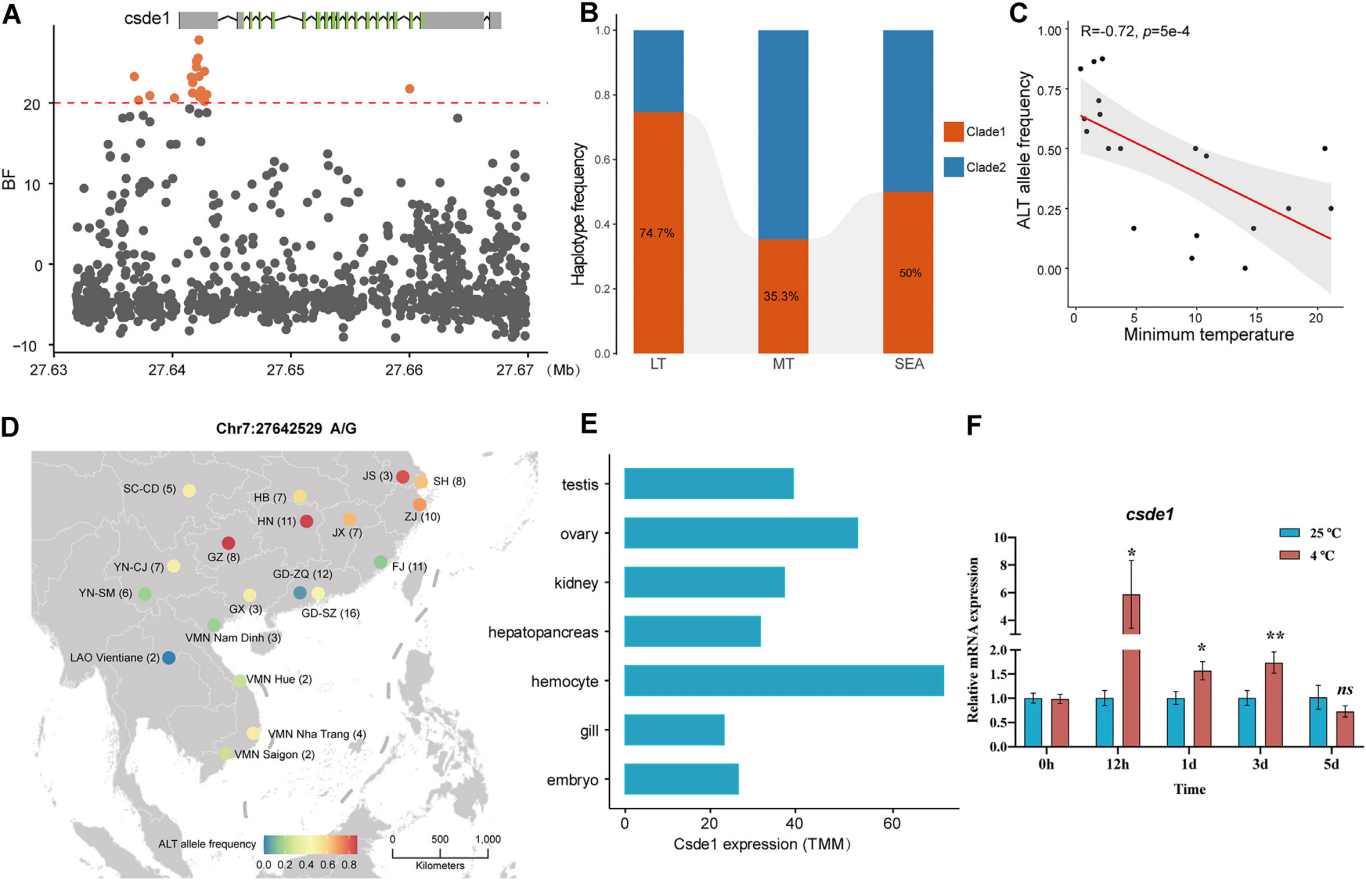
Genotype–environment association for the Min Temperature of the Coldest Month in different sampling locations on the *CSDE1* gene. (A) BF value in the *CSDE1* region. The horizontal red dashed line (BF > 20 dB) corresponds to the chosen significance level for genotype–climate association. (B) Haplotype frequency in the *CDSE1* 5′-UTR region for different types of *P. canaliculata* accessions. LT: individuals from East Asia where the minimum temperature is below 2°C; MT: individuals from East Asia, except for LT individuals; SEA: individuals from Southeast Asia. (C) Significant negative correlation between the alternate allele frequency of the SNP (Chr7: 27642529 A/G) and the Min Temperature of the Coldest Month in different sampling locations. (D) Alternate allele frequency of the SNP (Chr7: 27642529 A/G) in the EA and SEA populations. (E) Expression level of *CSDE1* in different tissues of *P. canaliculata*. (F) RT-qPCR validation for *CSDE1* performed on hemocyte tissue of *P. canaliculata*, with 4 replicates at 4°C and 25°C (*P < 0.05, **P < 0.01, ^****^P < 0.0001 by Welch’s *t*-test).

In particular, we identified a number of genes such as *TRHR* and *CSDE1* that covered outlier SNPs highly relevant to temperature (Supplementary Fig. S13 and Supplementary Table S5). As a member of the G protein–coupled receptor superfamily, *TRHR* encodes a central thyrotropin-releasing hormone (TRH) receptor. The TRH system is known to be involved in thermoregulation and glucose metabolism, 2 important adaptive systems functioning during cold exposure [21]. Animals with TRH deficiency exhibit impaired cold tolerance and glucose metabolism [22, 23]. In addition, the cold shock domain containing E1 (*CSDE1*) gene, also known as upstream of N-Ras (UNR), codes for an RNA-binding protein (RBP) that has five cold-shock domains (CSDs). The cold-shock protein plays an important role in stress adaptation and low temperature tolerance, functions that are well characterized in bacteria and plants [24, 25]. Notably, we discovered 8 SNPs at the 5′-UTR regions of the *CSDE1* gene that were highly relevant to temperature (Fig. 3A). The posttranscriptional regulation of *CSDE1* [26] may be affected by these outlier SNPs in the 5′-UTR regions, which would further contribute to the cold adaptation. The median-joining network analysis revealed 12 haplotypes were clustered into 2 clades, and samples from LT regions were predominantly enriched in clade 1 (Fig. 3B, Supplementary Fig. S15). Moreover, we observed that an alternative allele (Chr7: g. 27642529 A>G) with the highest BF value within the *CSDE1* gene was strongly positively correlated with temperature (*ρ* = 0.518, *P* = 0.023) (Fig. 3C, D). Furthermore, we found that *CSDE1* was highly expressed in several tissues of *P. canaliculata*, especially in the hemocytes, ovary, and testis (Fig. 3E). To further investigate the dynamic expression of *CSDE1* in response to exposure to cold, we also carried out a reverse transcription quantitative polymerase chain reaction (RT-qPCR) experiment. We found that within the first 24 hours of exposure to the cold, the expression of *CSDE1* in the hemocytes dramatically increased (Fig. 3F) and then rapidly declined throughout the following 4 days. These findings provided evidence for the potential role of *CSDE1* in the cold-shock response.

**Figure 4:**
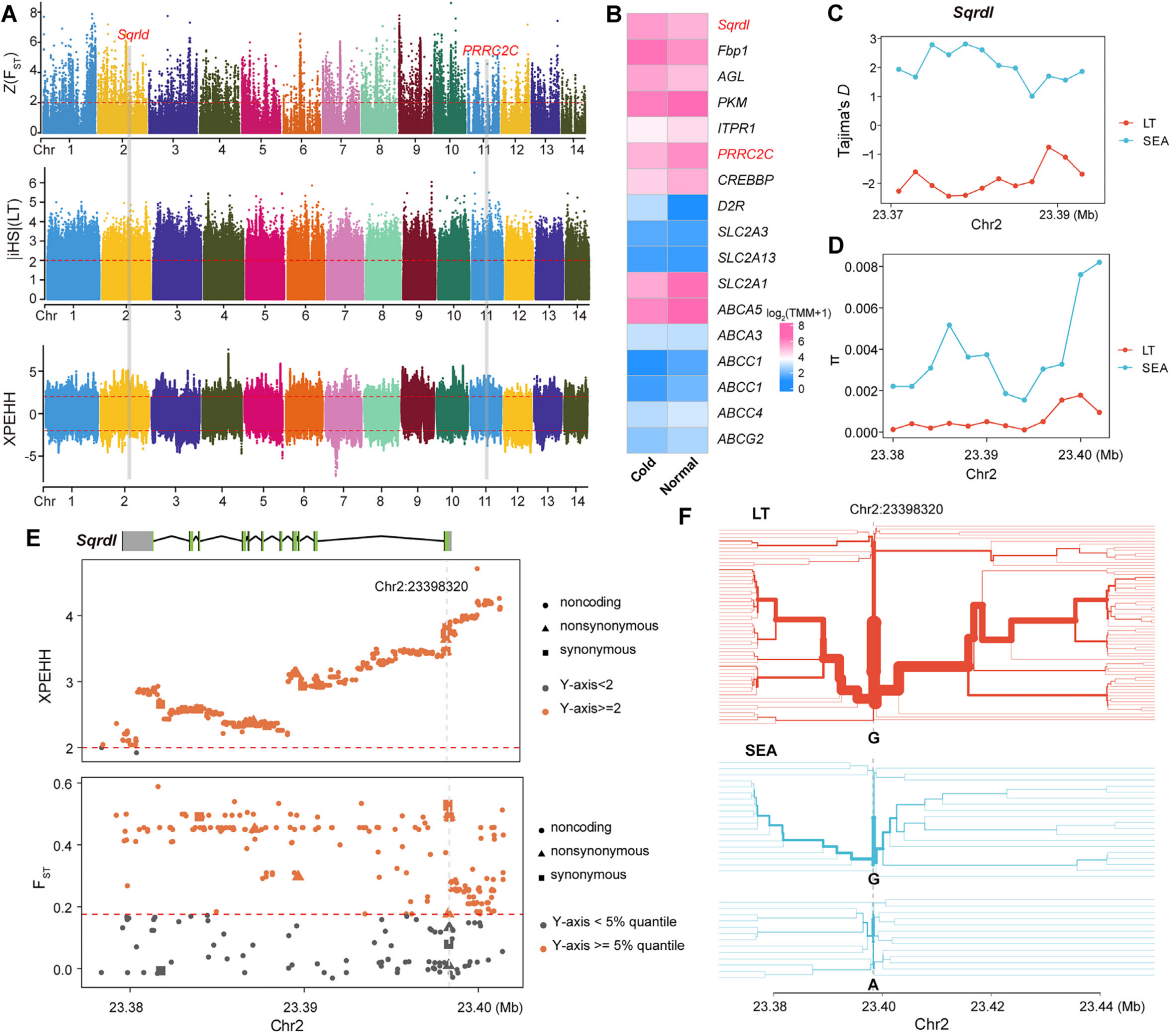
Positive selection scans for low-temperature adaptation in the LT populations of *P. canaliculata*. (A) Whole-genome scan with F_ST_, iHS, and XPEHH. F_ST_ is normalized as *z* scores for the *P. canaliculata* genome. The horizontal red dashed lines represent the empirical threshold for the selected regions. F_ST_: top 5% windows; iHS and XPEHH: 2. (B) Expression level of the positively selected genes under cold stress. Nucleotide diversity (C) and Tajima’s *D* statistic (D) in the *Sqrdl* gene for LT and SEA populations. (E) Multiple statistics indicating positive selection on the genomic region harboring the *Sqrdl* gene. The y-axis represents the normalized XPEHH (the first panel) and F_ST_ values (the second panel). Circles, triangles, and squares denote noncoding, nonsynonymous, and synonymous variants, respectively. (F) Haplotype bifurcation plots for LT and SEA haplotypes across chromosome 2 positions from 23.28 to 23.44 Mb. The colors of each plot reflect the location of sampling. Labels indicate nucleotides at the central position. LT: all individuals from East Asia where the minimum temperature is below 2°C; SEA: all individuals from Southeast Asia.

As BayPass is an environmental association analysis (EAA) for identifying subtle shifts in allele frequency associated with local adaptation [27], the programs have difficulty in detecting selective sweeps unique to 1 or few populations or sweeps concerning different haplotypes associated with the same gene. Therefore, we further investigated the genomic signature for different environments with standard genome-wide scan approaches. We performed selective sweep analyses (F_ST_, integrated haplotype scores [iHS], and cross-population extended haplotype homozygosity [XPEHH]; see Materials and Methods) to identify candidate genes involved in cold adaptation in the comparisons between low-temperature populations (LT, all individuals from EA where the minimum temperature is below 2°C) and high-temperature populations (HT, all individuals from the SEA population). Overall, we identified 750 nonredundant regions (total length = 7.04 Mb) that exhibited at least 2 extreme scores of Fst, iHS, or XPEHH, encompassing 754 genes (representing 3.33% of all coding genes) (Fig. 4A, Supplementary Table S6). Several genes bearing signals of positive selection in the LT population were associated with glycolysis (e.g., *Fbp1, AGL*, and *PKM*), in mediating the uptake of glucose (e.g., *Slc2A3, Slc2A13*, and *Slc2A1*), and in stress response (e.g., *ITPR1, PRRC2C, CREBBP*, and *D2R*). Functional analysis showed that these selected genes were significantly enriched for GO terms related to positive regulation of transporter activity (GO: 0032411, *P* = 3.51 × 10^−5^), regulation of skeletal muscle contraction (GO:0014819, *P* = 3.94 × 10^−5^), and regulation of calcium-mediated signaling (GO:0050848, *P* = 5.55 × 10^−5^) (Supplementary Table S7). It is notable that the *Sqrdl* gene encoding sulfide quinone oxidoreductase showed strong positive selection in the LT population supported by the elevated iHS, F_ST_, and XPEHH values (Fig. 4A, E). *Sqrdl* plays a key role in controlling H_2_S availability via oxidation for inhibiting mitochondrial respiration, thereby reducing energy during torpor or hibernation to respond to the cold stress [28]. A significantly lower Tajima’s *D* statistic and nucleotide diversity (π) were observed in the all LT individuals compared to the SEA populations (Fig. 4C, D), further supporting the hypothesis of positive selection in the LT population. Notably, 1 nonsynonymous variant (Chr2: g. 23398320) in the *Sqrdl* gene exhibited extreme XPEHH (normalized XPEHH = 3.80736) and F_ST_ (F_ST_ = 0.498969) values (Fig. 4E) and had a pronounced signature of natural selection (Fig. 4F). RNA sequencing (RNA-seq) [16] further supported *Sqrdl* being significantly upregulated under cold stress (foldchange = 1.4, *P*-adjust = 7.87e^−05^), pointing to a functional role of *Sqrdl* for cold adaptation (Fig. 4B).

Interestingly, we found 9 genes showing selective sweep signatures that were also identified in the BayPass analysis as being associated with temperature (Supplementary Table S8). In particular, the *PRRC2C* gene, which is involved in the formation of stress granules (SGs) [29], was focused on because strong selection signals were detected using all 3 of the above methods (Supplementary Fig. S16a). In addition, a significantly lower Tajima’s *D* statistic and nucleotide diversity (π) were observed in the LT population (Supplementary Fig. S16b, c), and significant differences between LT and SEA populations were observed in the extended haplotype homozygosity of the peak SNPs. These findings suggested that *PRRC2C* has undergone positive selection (Supplementary Fig. S16d, e). *PRRC2C* was significantly differentially expressed after exposure to cold according to the RNA-seq data (foldchange = 0.62, *P*-adjust = 6.92e^−09^, Fig. 4B, Supplementary Table S7). Remarkably, we observed that 1 variant located in the 5′-UTR (Chr11: g. 16598124) and 1 nonsynonymous variant (Chr11: g. 16572768) showed highly divergent frequencies between LT (98.47%) and SEA populations (53.12%; Supplementary Fig. S16a, f). The allele frequencies of these 2 *PRRC2C* gene variants were strongly positively correlated with temperature, suggesting that they may contribute to cold adaptation in the LT population (Supplementary Fig. S16g).

### Balancing selection contributed to the adaptive invasion

**Figure 5:**
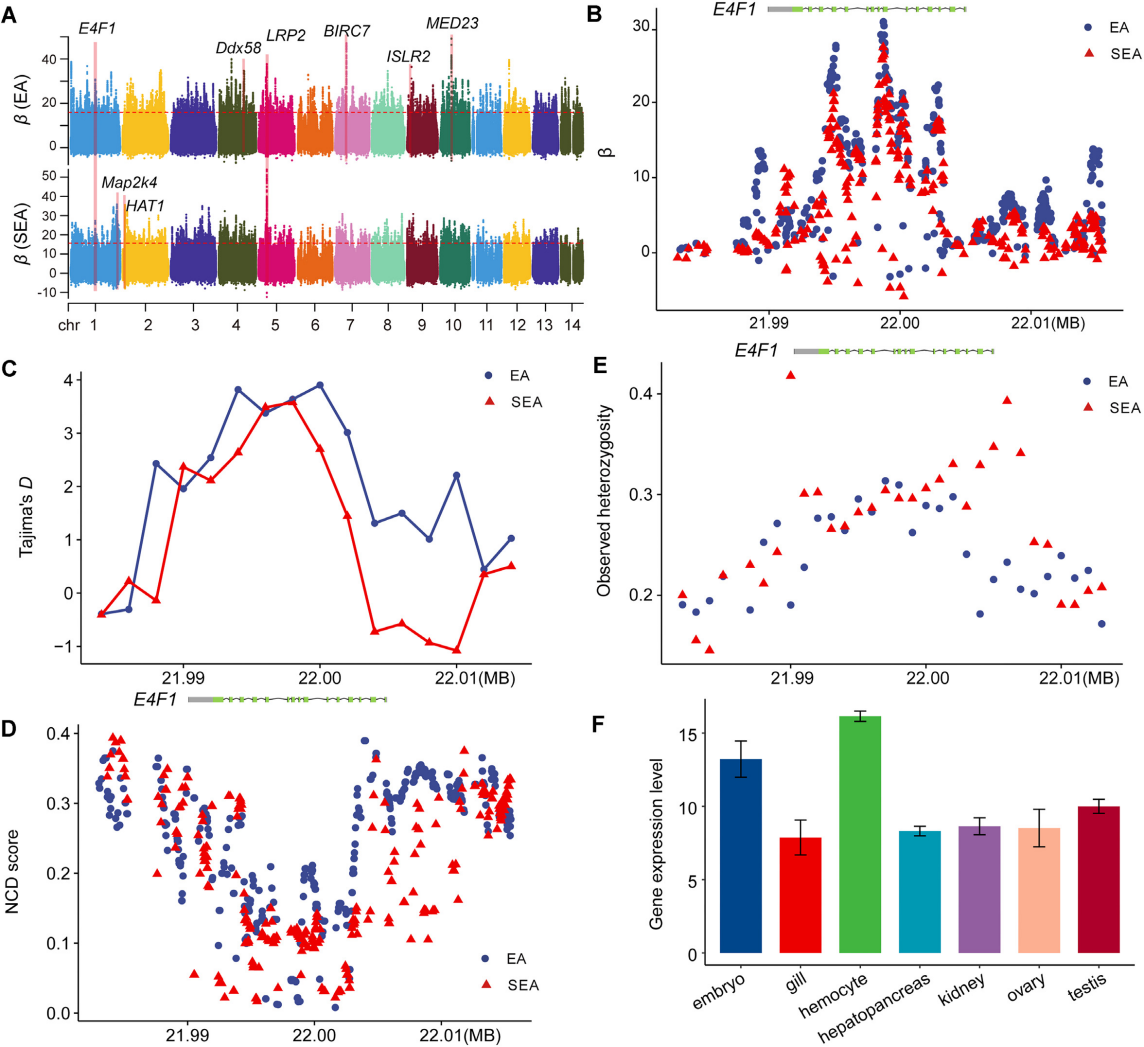
Balancing selection in the *P. canaliculata* population. (A) Regions of balancing selection detected in the EA and SEA populations based on *β* scores. The dashed line represents the significance level of the top 0.1% *β* score values. (B) Enlarged diagram of *β* statistics around the *E4F1* gene in the EA and SEA populations. Tajima’s *D* (C) and NCD (D) statistics around the *E4F1* gene in the EA and SEA population. (E) Diagram showing the heterozygous genotype frequency around the *E4F1* gene region, calculated as the mean of all variants in 1-kb non-overlapping windows. (F) Expression profile of the *E4F1* gene in different tissues of *P. canaliculata*.

When invasive species enter a new environment, population bottlenecks typically result in losses of genetic diversity. However, there are exceptions caused by other evolutionary processes that can facilitate invasion, including the maintenance of genetic diversity through balancing selection. Therefore, we searched for the genomic signatures of balancing selection using *β* scores and detected 1,000 regions covering 1,086 genes in the EA populations and 932 regions covering 1,027 genes in the SEA populations using top 0.5% value as the highest significance level (Fig. 5A, Supplementary Tables S9 and S10). The analysis revealed a high contribution from balancing selection. Notably, significant balancing selection signals were discovered in both the EA and SEA populations in a total of 235 genes, of which 199 genes showed differential expression levels in response to cold, heat, drought, or heavy metal stimulation (Supplementary Table S11). The overlap between balancing selection genes and differentially expressed genes was significantly higher than expected by chance (Fisher’s exact test, *P* < 2.2 × 10^−16^). Functional analysis showed that these balancing selecting genes in both the EA and SEA populations were highly enriched in the GO term related to response to radiation (GO:0009314, *P* = 0.000135), response to light stimulus (GO:0009416, *P* = 0.000436), larval lymph gland hemopoiesis (GO:0035167, *P* = 0.000912), and cellular response to organonitrogen compound (GO:0071417, *P* = 0.00130), which were probably associated with stress adaptation (*P* < 0.05; Supplementary Fig. S17). Besides, KEGG pathway enrichment analysis showed these overlap genes were generally enriched in pathways such as MAPK signaling pathway (map04010, *P* = 0.00765409) and cytokine–cytokine receptor interaction (map04060, *P* = 0.008744156) (Supplementary Fig. S18), which may be associated with stress responses and immune responses [30, 31].

Furthermore, genes exhibiting significant signals of balancing selection were found to be highly enriched in the GO terms associated with immune modulation in various body regions (*P* < 0.05), particularly in EA populations (Supplementary Fig. S19). Specifically, we observed significant signals in both the EA and SA populations for the *E4F1* gene (Pca0010750) (*β_EA_* = 30.73, *β_SEA_* = 27.31; Fig. 5b; Supplementary Table S10), a result that was validated by Tajima’s *D* and NCD statistics (Fig. 5C, D). The *E4F1* gene, a transcriptional regulator, has been reported to play an important role in maintaining animal innate immune [32]. In addition, several nonsynonymous SNPs within the *E4F1* gene exhibited increased heterozygosity, indicating a strong signal of balancing selection (Fig. 5E; Supplementary Fig. S21). The transcriptome data [33] also revealed that *E4F1* was preferentially expressed in the hemocytes, which are known to play essential roles in the internal defense mechanisms of mollusks [34] (Fig. 5F). We noticed that the *LRP2* gene showed signatures of balancing selection in both the EA and SEA populations (ranked as top 6 in EA and top 1 in SEA; Fig. 5A; Supplementary Table S11). Although specific mechanism studies are limited in mollusc, *LRP2* may be involved in development, pathogen recognition, and immune regulation processes. For example, insect defensin promotes the adsorption and infection of Japanese encephalitis virus through *LRP2* [35], suggesting a potential role for *LRP2* in host–pathogen interactions. Interestingly, we found that several genes exhibiting highly significant balancing selection signals were associated with immunity or stress tolerance. Theses genes include the *med23* [36], *BIRC7* [37], *ISLR2* [38], and *Ddx58* [39] in the EA populations (ranked among the top 10 genes, Fig. 5A; Supplementary Table S9), as well as the *Map2k4* [40] and *HAT1* [41] in the SEA populations (ranked among the top 3 genes, Fig. 5A; Supplementary Table S10). These genes were highly expressed in several tissues of *P. canaliculata*, especially in the gill, hemocytes, and hepatopancreas, which are crucial tissues of *P. canaliculata* relevant to detoxification and immunity (Supplementary Fig. S22). These findings illustrate that genes associated with immunity, detoxification, and stress tolerance, which were more likely to be subjected to balancing selection, contributed to the adaptive invasion of *P. canaliculata*.

## Discussion

The golden apple snail *P. canaliculata* has drawn considerable attention throughout the world due to its environmental invasiveness, wide range of stress adaptations, and rapid reproduction. We performed the first whole-genome analysis of 173 representative *P. canaliculata* individuals to comprehensively understand the genetic diversity and evolutionary history of this invasive species. Our findings confirmed the multiple origins and migrations of *P. canaliculata* using data at the whole-genome level that had already been discovered using mtDNA data [3]. *P. canaliculata* populations still possessed subpopulation genetic structure, indicating that they have experienced complex genetic interactions during the short period of invasion based on our whole-genome sequencing (WGS) data. Additionally, the genetic diversity of *P. canaliculata* was higher than that of other mollusks, and we found higher interpopulation genetic differentiation than intrapopulation genetic differentiation, pointing to a trend of rapid radiation throughout the Asian continent. Interestingly, several significant genetic barriers coincided with the Yangtze River Basin and international borders, implying that climate and human activity may have been the major factors influencing the dispersal of the invasive golden apple snail.

We identified a genetic barrier coinciding with climate, and numerous studies have revealed that low temperature in winter is a limiting factor in the geographic expansion and successful establishment of apple snail populations [12, 19, 20]. We selected the Min Temperature of the Coldest Month as the primary environmental factor and finally identified a set of candidate genes associated with temperature. The gene that stood out the most was *Csde1*, where certain haplotypes were more prevalent in samples from low-temperature zones. Intriguingly, a variant (Chr7: g. 27642529 A>G) in *Csde1* showed the highest alternative allele frequency in the majority of low-temperature regions but was absent in high-temperature regions (Guangzhou and Laos). This finding is consistent with a previous study that reported that individuals from these low-temperature regions had the highest survival rate and prolonged survival time regardless of the temperature acclimation treatment, whereas individuals from Guangzhou were the shortest lived [12]. Previous studies focused on the heat shock proteins (HSPs) in the invasive apple snail, which are supposed to play critical roles in how they adapt to harsh environments [13, 42, 43] and indicated that HSPs may be related to the thermal resistance of *P. canaliculata* [44]. We made the first effort to discover the *Csde1* associated with cold resistance in *P. canaliculata* and performed a transcriptional analysis and RT-qPCR validation to illustrate its dynamic pattern during cold exposure and biological functions; the result could be a potential and powerful genetic candidate for prevention and control of the invasive species.

We comprehensively analyzed and compared the genomic selection signatures of low- and high-temperature populations using multiple methods. The genome-wide scan identified a set of genes showing significant selective sweep signals. For example, *Sqrdl* had a pronounced signature of natural selection in low-temperature populations and was highly upregulated under cold stress based on RNA-seq data [16]. Besides, we proposed an approach in which genes with strong temperature association and significant selection signals in more than 2 selective sweep methods were defined as being more likely to be genes and alleles involved in cold adaptation. Eventually, 9 candidate genes were identified. The genes *pqn-25* and *PRRC2C* are reported to be linked to SGs [29, 45]. *Pka-C1* positively regulates cold stress [46] and plays a major role in providing cold adaptation and tolerance to freezing [47]. *Gyc32E* plays an important role in both cold and heat stress–induced pathways [48]. *Dopamine D2-like receptor* inhibits cold-initiated thermogenesis in brown adipose tissue [49]. Interestingly, RNA-seq analysis [16] revealed that *pqn-25, PRRC2C, Pka-C1*, and *Gyc32E* were significantly downregulated under cold stress. *Dopamine D2-like receptor* was the only gene that was highly upregulated, reflecting its negative regulation function in cold adaptation.

Adaptive evolution is one of the primary mechanisms that enable organisms to endure and flourish in new environments. *P. canaliculata*, which originated in South America and recently migrated into Asia, could be an excellent model for understanding how species rapidly adapt to new environments. We found 235 genes that indicated a high contribution from balancing selection, of which 199 genes showed differential expression in response to various stimuli. The proportion was significantly higher than expected by chance. We also found that many immune-related genes in both the EA and SEA populations had significant balancing selection signals. These immune-related genes could serve as an evolutionary basis for the continuous antagonistic coevolution between *P. canaliculata* and a wide range of pathogens in Asia. Balancing selection is a classic mechanism for maintaining variability in immune genes involved in host–pathogen interactions [50]. Overall, positive selection and balancing selection as important evolutionary forces are likely to have contributed to the rapid environmental adaptation of *P. canaliculata* populations in Asia.

## Materials and Methods

### Sample collection and sequencing

Individuals of *P. canaliculata* for genome assembly were collected from Shanghai, China. Using standard phenol/chloroform extraction, we extracted the genomic DNA of *P. canaliculata* from the foot tissue of a female individual. The integrity and concentration of genomic DNA (gDNA) were further assessed by gel electrophoresis and an Agilent Bioanalyzer 2100 (Agilent Technologies), respectively. Four paired-end libraries were constructed with insert sizes of 250 bp, 300 bp, 500 bp, and 2 kb and then sequenced on the next-generation sequencing (NGS) Illumina Hiseq X Ten platform (RRID:SCR_016385). To generate the ultra-long genomic reads, 20-kb genomic sequencing libraries were constructed and sequenced on the third-generation sequencing (TGS) PacBio SEQUEL platform (RRID:SCR_017989), yielding more than 30 Gb of subreads with an N50 length of 5.7 kb and the longest read of 150 kb. Ten grams of gDNA was also used for Hi-C library construction using a previously described method [51], followed by sequencing on the Illumina X Ten platform in the 150PE mode.

For RNA preparation and sequencing, ocular, skin, muscle, gonadal, intestinal, liver, kidney, blood, gallbladder, and air bladder tissues of *P. canaliculata* were combined, and total RNA was extracted from 50 mg of composite samples using the TRIZOL Reagent (Invitrogen). Size selection of 0–3 kb and 2–6 kb was performed using the BluePippin Size Selection System (Pacific Biosciences). SMRTbell Template libraries were constructed with complementary DNA (cDNA) products using a SMRTBell Template Prep Kit, then subjected to 1 or 2 cells on the PacBio SEQUE platform (Pacific Biosciences). A library with an insert length of 250 bp was also sequenced on Illumina HiSeq 2000 in the 150PE mode.

### Genome assembly of Pcan_SH and assessment

The long reads generated by the PacBio SEQUEL platform were assembled with FALCON (RRID:SCR_023199) [52] using a series of parameters. We found that the assembly size and contig N50 both increased with reducing length cutoff of self-corrected long reads used for assembly, while the assembled genome size and N50 length reached plateaus of ∼560 Mb and ∼280 kb, respectively, when the length cutoff was less than 6 kb. We further assembled the genome using PacBio long reads with CANU (RRID:SCR_015880) [53] and RACON (RRID:SCR_017642) [54], leading to contig N50 lengths of 196 kb and 550 kb, respectively. We then purged haplotigs and overlaps in the contigs assembled by FALCON based on the PacBio read depth using purge_dup software (RRID:SCR_021173). The final contigs were further polished by arrow [55] with TGS reads and pilon (RRID:SCR_014731) [56] with NGS reads. Chromosomal assembly of *P. canaliculata* was carried out using Hi-C data. Raw Hi-C reads were polished and filtered using hiclib as described previously [57]. Lachesis was applied to cluster the final contigs into 14 groups using the agglomerative hierarchical clustering method and was further used to order and orient the clustered contigs (Pcan_SH).

Core gene mapping ratios greater than 96% were obtained from both CEGMA (RRID:SCR_015055) [58] and BUSCO (RRID:SCR_015008) [59], validating the completeness of the assembled genome. By mapping NGS reads to the final genome, more than 98% of NGS short reads were mapped to the genome, and 96% were paired aligned, further confirming the correctness of the genome assembly.

### Genome annotation

Tandem repeats of the *P. canaliculata* genome were detected using Tandem Repeats Finder. Transposable elements (TEs) were identified using a combination of homology-based and *de novo* approaches. RepeatModeler (RRID:SCR_015027) was used initially to generate a *de novo* repeat library that was then combined with the known repetitive sequences (e.g., RepBase 17.01). The TEs in the *P. canaliculata* genome were further identified by mapping to the library using the software RepeatMasker (RRID:SCR_012954) [60]. Finally, a total of 132.96 Mb of the sequences were identified as TEs, comprising 22.79% of the genome.

Gene prediction was performed with *de novo*, homology-based, and sequencing-based methods to annotate the *P. canaliculata* genome. We used Augustus (RRID:SCR_008417) [61] to predict coding genes via *de novo* prediction. For homology-based prediction, protein sequences were downloaded from Ensembl [62] for closely related mollusk species, including *Aplysia alifornica, Biomphalaria glabrata, Crassostrea gigas, Lottia gigantea*, and *Mizuhopecten yessoensis*. These sequences were aligned against the *P. canaliculata* genome using TBLASTN software (RRID:SCR_011822) [63]. GeneWise (RRID:SCR_015054) [64] was then used to define gene models for the *P. canaliculata* genome. For the sequencing method, full-length transcriptomes from Iso-seq were first aligned to the genome using GMAP (RRID:SCR_008992) [65] software, providing reliable gene structures for the genome. In addition, NGS transcriptome short reads were also used to align the genome using the TopHat package [66], and the gene structure was predicted using cufflinks [67]. All gene models were then integrated by MAKER (RRID:SCR_005309) [68] to obtain a consensus gene set.

For functional annotation of protein-coding genes in *P. canaliculata*, all gene sequences were searched against NCBI nonredundant protein (nr), nonredundant nucleotide (nt), and Swissprot databases using local BLASTX and BLASTN programs [63] with an e-value of 1e^−5^. GO and KEGG pathway searches were then conducted on the resulting transcriptome using the software Blast2GO (RRID:SCR_005828) [69].

### Detection of chromosomal rearrangement between reference genomes

To identify chromosomal rearrangements between genomes of Pcan_SH and previously published Pcan_SZ, the 2 genomes were first aligned using MUMmer4 (v4.00beta2) [70]. Alignment of the genomes was performed using NUCmer (-c 1000), and then the alignment block filter was performed using a delta-filter with one-to-one alignment mode (-1 -i90 -l 10000). Finally, chromosomal rearrangements were called using the SyRI tool (RRID:SCR_023008) (v1.4) with default parameters [71], and Plotsr (v1.1.0) [72] was conducted to generates high-quality visualization of synteny and structural rearrangements.

### Whole-genome population resequencing

We sampled 173 wild *P. canaliculata* individuals from 17 geographic distribution areas in EA and SEA for genome resequencing. Among the samples, 157 were collected from 12 provinces of China, with the remaining 16 samples being obtained from SEA countries (11 from Vietnam, 3 from Laos, and 2 from Cambodia) (Supplementary Table S1). Genomic DNA was extracted from the foot tissue using the DNeasy Blood & Tissue Kits (QIAGEN). Two micrograms of gDNA from each individual was used to construct a sequencing library using a NEBNext Ultra DNA Library Prep Kit (NEB) following the manufacturer’s instructions. Paired-end sequencing libraries with an insert size of approximately 350 bp were sequenced on an Illumina NovaSeq 6000 platform (RRID:SCR_016387) at Novogene-Beijing. All samples were sequenced to a target coverage of 10×. In addition, we downloaded the resequencing data for an individual from Argentina (accession number: SRR8616636) and 1 *P. maculata* sample as an outgroup (accession number: SRR8616630) reported in a previous study [33].

### Variant calling, filtering, and annotation

We applied fastp (RRID:SCR_016962) [73] to filter the raw sequencing reads using the default parameters. The filtered reads were aligned to the new reference genome of *P. canaliculata* using BWA-MEM (RRID:SCR_022192) [74] with the -M parameter, and duplicates were marked using PicardTools MarkDuplicates (as part of GATK) [75]. Since whole-genome SNP and INDEL databases of *P. canaliculata* were not available to perform the Base Quality Score Recalibrator (BQSR), we performed BQSR of nonhuman genomic data following GATK. We performed an initial round of joint-call cohort genotyping using the GATK HaplotypeCaller in gGVCF mode and GATK GenotypeGVCFs in succession. We then filtered variants with low quality using GATK VariantFiltration based on the following criteria: QD < 2.0, FS > 60.0, MQ < 40.0, SOR > 3.0, MQRankSum < −12.5, ReadPosRankSum < −8.0, QUAL < 30.0 for SNPs and QD < 2.0, FS > 200.0, SOR > 10.0, MQRankSum < −12.5, ReadPosRankSum < −20.0, and QUAL < 30.0 for INDELs. The variants passing the hard filtration were used as a true-positive set of variant sites for BQSR with GATK BaseRecalibrator. We then repeated the joint-call cohort genotyping with the recalibrated BAM files and retained the variants if they met the above criteria.

Using VCFtools (RRID:SCR_001235) [76], we assigned the genotypes as missing if their quality scores were less than 10 and excluded 1 sample with a high rate of missing SNPs (>30% of sites with a missing genotype). We used the KING software [77] to calculate kinship coefficients between all pairwise combinations of samples. Forty samples exhibiting greater than third-degree relationships with others were removed, leaving a total of 130 samples for subsequent analysis. Variants with none, biallelic, >5% missing calls, and minor allele frequency (MAF) <0.01 were removed to reduce false positives. The SNPable with 75-mer parameter and mDust procedures were used to mask regions of low mappability, and sites within these were also removed. This yielded a total of ∼13.55 million variants for downstream analyses. Functional annotation of the retained variants was performed using the software ANNOVAR (RRID:SCR_012821) [78] with gene annotation for *P. canaliculata*.

### Population genetic analysis

We pruned variants for LD in PLINK (RRID:SCR_001757) [79] with parameters –indep-pairwise 50 5 0.1 and –maf 0.05, which retained 266,653 SNPs for analysis of population structure. PCA was conducted at the individual level using the smartpca from the EIGENSOFT program [80] with the pruned SNP datasets. An ML phylogenetic tree was constructed by RAxML software (RRID:SCR_006086) [81] with the GTRGAMMA model and 1,000 bootstrap replicates. *P. maculata* was used as the outgroup. Software Admixture (RRID:SCR_001263) [82] was used to infer population genetic structure. Ten independent replications were performed for each of the ancestral numbers (*K*) from 2 to 10. The optimal *K* was determined according to the position with the minimum value of the 5-fold cross-validation error.

VCFtools [76] was used to calculate the fixation index (F_ST_), nucleotide diversity (π), and Tajima’s *D* in 5-kb sliding, non-overlapping windows across each chromosome. Windows with fewer than 20 variants per 5-kb window were removed. Regression between the pairwise genetic distance (F_ST_/(1 − F_ST_)) and geographic distance was calculated using a Mantel test as implemented in the Ecodist package for R. The significance of correlations was determined based on 1,000 permutations. LD decay was estimated for each population using the PopLDdecay tool (RRID:SCR_022509) [83] that calculates the genotype correlation coefficient *R*^2^ for pairs of SNPs at a maximum distance of 5 kb. The LD decay was measured as the chromosomal distance at which the average pairwise correlation decreased to half its maximum value.

### Population splits and mixtures

TreeMix was applied to investigate the historical population relationships by estimating an ML population tree, the amount of genetic drift in each population, and the number of migration events (*m*) that best fitted the data [84]. *P. maculata* was used as a root. Variants with missing rate 1% or a minimum allele frequency <0.05 in all samples were filtered out for further TreeMix analysis. In addition, we pruned any SNPs that were in LD using PLINK (–indep-pairwise 50 5 0.2) and retained 195,777 variants. We first ran TreeMix 20 times for each value of *m* ranging from 1 to 10 (-global -k 500 -se -bootstrap -noss). The optimal *m* value (*m* = 2) was estimated using the OptM R package [85]. Then, a consensus ML tree including bootstrap node support was obtained by running TreeMix 100 times for zero (as a null model) and 7 migration events, followed by postprocessing using the BITE R package.

The outgroup *f3* statistics were also estimated to infer the genetic affinities between the SEA populations and all other populations of *P. canaliculata*. To compute outgroup *f3* statistics of the form *f3* (X, Y; *P. maculata*) where *P. maculata* was selected as the target population, we applied the qp3pop module in the ADMIXTOOLS software [86].

Spatial variation in gene flow was investigated using EEMS analysis using 130 individuals, 5,000,000 Markov chain Monte Carlo (MCMC) iterations, a burn-in of 1,000,000 iterations, and a thinning iteration of 9,999 for each run. Parameters with 400 demes were carried out and plotted using rEEMSplots as the recommendation [87]. The habitat polygon was obtained using the Google Maps API v3 Tool, and an individual genetic dissimilarity matrix was created using the bed2diffs function of EEMS.

### Detecting genomic signatures for low-temperature adaptation

The BayPass program was used to identify SNPs with frequencies that were significantly associated with low temperature. For the ecotype divergence test, we retrieved the environmental variable Bio06 (Min Temperature of Coldest Month) for 17 geographic populations through the raster package in R and scaled the results so that the mean = 0 and variance = 1 as recommended [88]. Capitalizing on the large number of available SNPs, we subsampled by retaining 1 SNP every 100 SNPs along the genome, dividing the full SNP dataset into 100 sub-datasets (each including ca. 135,544 SNPs). These sub-datasets were further analyzed in parallel using default options for the MCMC algorithm (except -npilot 15 -pilotlength 500 -burnin 2500). Three independent runs were performed for each dataset. We confirmed that the distance of covariance matrices (Ω) between replicates and between different sub-datasets was very low (fmd.dist < 1 as recommended in the BayPass manual), using the R function fmd.dist() included in BayPass. We also confirmed that all the obtained BF values across replicates had high correlations (*r* > 0.7). SNPs showing the median BF computed over the 3 runs greater than 20 dB were classified as outlier SNPs supported the significant association with low temperature.

Given that this study sought to characterize adaptation to climate, all individuals from Zhejiang, Shanghai, Jiangsu, Hubei, and Hunan provinces in EA, where the minimum temperature is below 2°C (LT), and SEA (HT) with high temperatures were selected for selection analysis. Two different haplotype-based methods (iHS, XP-EHH) and 1 allele frequency-based method (F_ST_) were used to detect genomic signatures of positive selection. The pairwise population differentiation coefficient (F_ST_) between the all LT populations and SEA populations was computed by VCFtools using a 10-kb sliding window with a step size of 5 kb [76]. We empirically selected the top 5% F_ST_ values as potential candidate regions under selection. After phasing the SNP dataset using SHAPEIT2 [89], we calculated the iHS and XP-EHH using Selscan [90] for each chromosome separately. The XP-EHH score was positive, reflecting the presence of extended haplotypes in the LT population. Using the norm module implemented in Selscan, the *P. canaliculata* genome was divided into non-overlapping 10-kb regions, and both the fraction of XP-EHH scores >2 and that of |iHS| >2 were computed. The top 5% of windows with the highest fraction of extreme scores were considered candidate selective regions. To reduce the false-positive regions in the detection, potential candidate regions defined by at least 2 of the abovementioned methods were considered the final candidate regions for selection.

### Detecting genomic signatures of balancing selection

Genomic scans for balancing selection (BS) were performed for the EA and SEA populations using the standardized *β* and NCD statistics. To reduce false positives, genotypes were marked as missing when the proportion of reads that uniquely mapped was below 80%. Further, we retained the genotype only when the depth of variants was at least one-third of the sample average read depth and no more than 2-fold of the read depth. Only SNPs with a MAF >0.05 and missing call rate <5% in each population were retained for balance selection. High *β* scores indicated an excess of SNPs at similar frequencies, while low NCD scores indicated a buildup of SNPs near a specified intermediate frequency, both of which are potential consequences of long-term BS. For standardized *β* scores, we applied the toolkit glactools [91] for file format conversion and ran BetaScan software [92] to calculate the *β* score to detect BS with the parameter “-fold -m 0.15” that refers to the minimum fold frequency of core SNPs. The conserved BS sites were identified as those SNPs with standardized *β* scores in the top 99.5th percentile in each population, and the sliding 10-kb windows with 2 or more such outlier SNPs were defined as the BS genomic regions. The NCD statistics measure the average difference between allele frequencies in a given region from a deviation point, while BetaScan measures *β* scores for individual SNPs. To facilitate comparison between the 2 statistics, a custom Python script was used to calculate a modified NCD statistic for each SNP in both SEA and EA populations with windows of 500 bp around every SNP and considering a target frequency of 0.5 [93]. Three additional statistics—namely, Tajima’s *D*, nucleotide diversity (π), and observed heterozygosity (*Ho*)—were applied to confirm the top signals.

### Functional enrichment analyses

Approximate gene annotations were obtained by assigning the candidate selective regions to their closest gene model in the *P. canaliculata* genome using BEDOPS [94]. GO enrichment tests were performed to detect functional groups using the clusterProfiler package [95] in R. An unadjusted *P* value <0.01 was assumed as the threshold for significant enrichment.

### Differential gene expression

We identified gene expression profile of *P. canaliculata* in 7 tissues, including embryos, gill, hemocytes, hepatopancreas, kidney, ovary and albumen gland, and testis with the public RNA-seq data [33] (BioProject PRJNA473031). Besides, RNA-seq data of *P. canaliculata* under different abiotic stress conditions were also analyzed, including heat, cold, heavy metal tolerance, and air exposure (BioProject PRJNA427478). Reads were downloaded from the SRA database [16] and trimmed off adapters and low-quantity bases with TrimGalore. Trimmed reads were then mapped to the *P. canaliculata* genome using HISAT2 (RRID:SCR_015530) [96], and the gene raw read count was obtained with featureCounts in Subread (RRID:SCR_009803) [97]. DESeq2 [98] in R was used to identify differentially expressed genes (DEGs). A gene with a fold change >1.2 (upregulated) or <0.83 (downregulated) and false discovery rate (FDR)-adjusted *P* < 0.05 was considered a DEG. The genes identified among the selection results were selected for plotting using R.

### Reverse transcription quantitative polymerase chain reaction validation of the *csde1* gene expression under cold tolerance

Snails with similar size were reared in freshwater at 25°C for at least 10 days for acclimation and then randomly divided into 2 groups with 3 replicates of 5 snails each. The control group was exposed to normal temperature (25°C), while the experimental group was exposed to 0°C for 5 days in an incubator. The hemocytes were then collected at 0 hours, 12 hours, 1 day, 3 days, and 5 days after exposure. Total RNA was extracted with TRIzol reagent (Takara Bio) and assessed using a Nanodrop 2000 spectrophotometer (Nanodrop Technologies). Reverse transcription quantitative polymerase chain reaction (RT-qPCR) was performed to further investigate the expression of the *csde1* gene in each sample in duplicate using SYBR qPCR Master Mix (Vazyme) in a 20-μL reaction volume. Primers for qPCR were designed with Primer Premier v5 with *β-actin* as the internal control (Supplementary Table S12). The relative expression levels of the *csde1* genes were calculated by the comparative cycle threshold (Ct) method (2 ^−ΔΔCt^) and subjected to statistical analysis with Prism v9.

## Supplementary Material

giae064_GIGA-D-23-00302_Original_Submission

giae064_GIGA-D-23-00302_Revision_1

giae064_GIGA-D-23-00302_Revision_2

giae064_Response_to_Reviewer_Comments_Original_Submission

giae064_Response_to_Reviewer_Comments_Revision_1

giae064_Reviewer_1_Report_Original_Submission Jacob Tennessen -- 10/30/2023 Reviewed

giae064_Reviewer_1_Report_Revision_1 Jacob Tennessen -- 2/19/2024 Reviewed

giae064_Reviewer_1_Report_Revision_2 Jacob Tennessen -- 7/1/2024 Reviewed

giae064_Reviewer_2_Report_Original_Submission Conghui Liu -- 11/13/2023 Reviewed

giae064_Reviewer_2_Report_Revision_1 Conghui Liu -- 2/26/2024 Reviewed

giae064_Supplemental_Files

## Data Availability

The genome sequence data for *P. canaliculata* are deposited in NCBI under SRA accession number PRJNA951867. The assembly and annotation files are available under the NCBI accession PRJNA951865. The whole genome resequencing data for *P. canaliculata* can be accessed with accession number PRJNA951872 in NCBI. All additional supporting data are available in the *GigaScience* repository, GigaDB [99].

## Additional Files

**Supplementary Fig. S1**. Circos diagram illustrating the features of the chromosome-level *Pomacea canaliculata* genome. Chr: chromosome. The outer circle represents the chromosome length of *P. canaliculata*, with units in Mb. Tracks (outer to inner circles) depict the following: (a) the length of contigs (alternating dark and light colors); (b) GC content (window size of 200 kb); the sequencing depth of the third-generation (c) and the next-generation (d) sequencing data; (e) gene density (gene number per 200 kb window); (f) transposable element density (window size of 200 kb); (g) tandem repeat density (window size of 200 kb); and distribution of segmental (h) and tandem duplication gene (i).

**Supplementary Fig. S2**. Hi-C interaction heatmaps for genome assemblies of *P. canaliculata*. (a) Hi-C interaction heatmap for Pcan_SH genome assembly. The linear sequence of the reference genome assembly is represented on both axes, and the diagonal shows 3D proximity of interacting pairs. No off-diagonal interactions are visible. (b) Hi-C interaction heatmap for Pcan_SZ genome assembly. The chromosome names in Pcan_SZ are ordered based on the Pcan_SH chromosome order using the genome synteny analysis. A number of off-diagonal interactions, marked with the black arrows, remain visible. These may arise from either missing links between scaffolds of the same chromosome or from misassembly.

**Supplementary Fig. S3**. Visualization of chromosome synteny and rearrangement between the 2 assemblies of *P. canaliculata*. Horizontal blue line represents Pcan_SH, and the orange line represents Pcan_SZ.

**Supplementary Fig. S4**. Hi-C validation for translocation and inversions between 2 genome assemblies of *P. canaliculata*. (a) Detailed view of intrachromosomal translocation on chromosome 14. (b) Detailed view of the inversion spanning the regions from approximately 39 to 45 Mbp on chromosome 1. (c) Detailed view of the inversion spanning the regions from approximately 6.0 to 20 Mbp on chromosome 8. The translocation and inversions are supported by the high-density contacts pointed by the red square in Hi-C heatmaps generated from Pcan_SZ Hi-C reads aligned to the Pcan_SZ genome (bottom), while no corresponding contract is found in the Pcan_SH Hi-C heatmaps (middle). These results indicate accurate genome assembly of Pcan_SH.

**Supplementary Fig. S5**. SNP density plot chromosome-wise representing number of SNPs within a 0.1-Mb window size. The horizontal axis shows the chromosome length (Mb), and the different color depicts SNP density.

**Supplementary Fig. S6**. Evaluation for the population structure inferred with Admixture. (a) Plot of ADMIXTURE cross-validation error from K = 2 through K = 10. We chose K = 4 as the best K due to the value that minimizes the error. (b) Evaluation of the admixture proportions under the best K = 4 model inferred by using the evalAdmix program. The upper diagonal shows the correlation of residuals between individuals and the lower the mean correlation within populations.

**Supplementary Fig. S7**. Nucleotide diversity (π) estimated for each population of *P. canaliculata*. Populations with samples fewer than 3 were removed.

**Supplementary Fig. S8**. Linkage disequilibrium decay estimated for different populations of *P. canaliculata*. Populations with samples fewer than 3 were removed.

**Supplementary Fig. S9**. Determination of the optimal number of migration edges between 0 and 15 in TreeMix using the (a) log-likelihood values and percentage of variance explained and (b) the second-order rate of change in the log-likelihood (Evanno) method.

**Supplementary Fig. S10**. TreeMix-inferred population tree and Residual fits for different migration events. (a) TreeMix consensus tree and bootstraps representing the relationships among populations for no migration edge (M0). Residual fit of the observed versus the predicted squared allele frequency difference, expressed as the number of SE of the deviation for M0 (b) and M7 (c). SE values are represented by colors according to the palette on the right. Residuals above zero indicate populations that are more closely related to each other in the data than in the best-fit tree and have potentially undergone admixture. Negative residuals represent populations that are less closely related in the data than represented in the best-fit tree.

**Supplementary Fig. S11**. Outgroup *f*3 statistics between the Argentina (a), VNM (b), LAO (c), KHM (d), and all other populations of *P. canaliculata*. Outgroup *f*3 statistics in the form *f*3 (X, Y; *P. maculata*) where *P. maculata* was selected as the target population.

**Supplementary Fig. S12**. EEMS posterior probability of effective migration (*m*) estimated in EEMS.

**Supplementary Fig. S13**. Genome-wide association with the Min Temperature of Coldest Month covariable using BayPass. The horizontal red dashed line (BF >20 dB) corresponds to the chosen significance level for genotype–climate association.

**Supplementary Fig. S14**. Emapplot of the GO function enrichment with the selected genes associated with the Min Temperature of Coldest Month covariable. The GO category was grouped using ClusterProfiler R package.

**Supplementary Fig. S15**. Haplotype network of the 5′-UTR in *CSDE1* gene. We used median-joint network analysis to construct the haplotype network of the 5′-UTR in *CSDE1* gene among all *P. canaliculata* accessions. The haplotypes that were present in a single accession were excluded. The node size and the parallel lines on branches represent the relative haplotype frequency and the number of mutations, respectively. The different colored portions in each node represent the proportion of different *P. canaliculata* accessions within each haplotype.

**Supplementary Fig. S16**. Signatures of positive selection in LT population and genotype–environment association on the *PRRC2C* gene. (a) Multiple statistics representing genotype–environment association and positive selection on the genomic region harboring the *PRRC2C* gene. The y-axis presents the Bayles factor value (the first panel) and the normalized XPEHH (the second panel) and F_ST_ values (the third panel). Circles, triangles, and squares denote variants under noncoding, UTR, and exonic regions, respectively. Nucleotide diversity (b) and Tajima’s *D* statistic (c) in the PRRC2C gene for LT and SEA populations. Haplotype bifurcation plots for LT and SEA haplotypes with the peak SNPs of Chr11: g. 16572768 (d) and Chr11: g. 16598124 (e). Colors of each plot reflect the location of sampling. Labels indicate nucleotides at central position. (f) Alternate allele frequency of the SNPs (Chr11: g. 16572768 and Chr11: g. 16598124). (g) The significantly positive correlation between the alternate allele frequency of the SNPs (Chr11: g. 16572768 and Chr11: g. 16598124) and Min Temperature of Coldest Month in different sampling locations.

**Supplementary Fig. S17**. Bar plot of the GO function enrichment with the balancing selected genes in both EA and SEA populations; the top 10 GO terms are showed in here. The GO category was grouped using ClusterProfiler R package.

**Supplementary Fig. S18**. Diagram plot of the KEGG pathway enrichment with the balancing selected genes in both EA and SEA populations. The pathway category was grouped using ClusterProfiler R package.

**Supplementary Fig. S19**. Emapplot of the GO function enrichment with the balancing selected genes in the EA population. The GO category was grouped using ClusterProfiler R package.

**Supplementary Fig. S20**. Emapplot of the GO function enrichment with the balancing selected genes in the SEA population. The GO category was grouped using ClusterProfiler R package.

**Supplementary Fig. S21**. Diagram showing the nonsynonymous SNPs and heterozygous genotype frequency in the *E4F1* gene region. The functions of SNPs were defined and predicted by Annovar.

**Supplementary Fig. S22**. Expression profile of the balancing selected genes in different adult tissues of *P. canaliculata*. The sequencing data were downloaded in NCBI under accession PRJNA427478.

**Supplementary Table S1**. Summary statistics of assembly and annotation for the *Pomacea canaliculata* genomes.

**Supplementary Table S2**. Geographical distribution and resequenced data information of *P. canaliculata*. BIO1, BIO5, and BIO6 for 17 geographic populations were retrieved through the raster package in R. EnvO is a community ontology for the concise, controlled description of environments.

**Supplementary Table S3**. Summary statistics of sequencing information for *P. canaliculata* accessions.

**Supplementary Table S4**. Pairwise FST values between population of *P. canaliculata*. The different color depicts the ancestral populations inferred by Admixture. Sequencing locations with samples fewer than 3 were removed.

**Supplementary Table S5**. Top 20 candidate windows associated with the Min Temperature of Coldest Month covariable using BayPass.

**Supplementary Table S6**. Annotation of putative selected genes in LT populations identified by at least 2 selective sweep analyses (Fst, iHS, or XPEHH).

**Supplementary Table S7**. Top20 GO enrichment of the positively selected genes in LT population identified with at least 2 methods of FST, iHS, or XP-EHH.

**Supplementary Table S8**. List of protein-coding genes identified by both bayPass results and selective sweep analyses.

**Supplementary Table S9**. Top 50 candidate genomic regions and annotated genes that detected balancing selecetion in the EA population.

**Supplementary Table S10**. Top 50 candidate genomic regions and annotated genes that detected balancing selection in the SEA population.

**Supplementary Table S11**. List of protein-coding genes exhibiting significant balancing selection signals in both EA and SEA popuations.

**Supplementary Table S12**. Primers used in this study.

## Abbreviations

BF: Bayes factor; BLAST: Basic Local Alignment Search Tool; bp: base pairs; BQSR: Base Quality Score Recalibrator; BS: balancing selection; BUSCO: Benchmarking Universal Single-Copy Orthologs; cDNA: complementary DNA; COI: cytochrome oxidase subunit 1; CSD: cold-shock domain; CV: cross-validation; EA: East Asia; EAA: environmental association analysis; EEMS: estimated effective migrations surfaces; gDNA: genomic DNA; GO: Gene Ontology; HSP: heat shock protein; HT: high-temperature populations; iHS: integrated haplotype scores; KEGG: Kyoto Encyclopedia of Genes and Genomes; LD: linkage disequilibrium; LT: low-temperature populations; MCMC: Markov chain Monte Carlo; ML: maximum likelihood; mtDNA: mitochondrial DNA; NCBI: National Center for Biotechnology Information; NGS: next-generation sequencing; PC: principal component; PCA: principal component analysis; RBP: RNA-binding protein; RNA-seq: RNA sequencing; RT-qPCR: reverse transcription quantitative polymerase chain reaction; SEA: Southeast Asia; SG: stress granule; SH: Shanghai; SNP: single nucleotide polymorphism; TE: transposable element; TGS: third-generation sequencing; TRH: thyrotropin-releasing hormone; UNR: upstream of N-Ras; XP-EHH: cross-population extended haplotype homozygosity; ZJ: Zhejiang.
