# Supplementary material for: Whole-genome sequencing of the invasive golden apple snail Pomacea canaliculata from Asia reveals rapid expansion and adaptive evolution
Source: Gigascience. 2024 Sep 23;13:giae064. doi: 10.1093/gigascience/giae064 (PMC11417965; doi:10.1093/gigascience/giae064)
Supplement: giae064_GIGA-D-23-00302_Revision_2 [file giae064_giga-d-23-00302_revision_2.pdf]

GigaScience

Whole-genome sequencing of the invasive golden apple snail Pomacea canaliculata from Asia reveals rapid expansion and adaptive evolution

--Manuscript Draft--

**Manuscript Number:**	GIGA-D-23-00302R2
**Full Title:**	Whole-genome sequencing of the invasive golden apple snail Pomacea canaliculata from Asia reveals rapid expansion and adaptive evolution
**Article Type:**	Research
**Funding Information:**	Key Technologies Research and Development Program (2016YFC1200503) — Professor Wei Hu<br>Key Technologies Research and Development Program (2021YFC2300800, 2021YFC2300802, 2021YFC2300803) — Dr. Yan Lu
**Abstract:**	Pomacea canaliculata, an invasive species native to South America, is recognized for its broad geographic distribution and adaptability to a variety of ecological conditions. The details concerning the evolution and adaptation of P. canaliculate remain unclear due to a lack of whole-genome re-sequencing data. We examined 173 P. canaliculata genomes representing 17 geographic populations in East and Southeast Asia. Interestingly, P. canaliculata showed a higher level of genetic diversity than other mollusks, and our analysis suggested that the dispersal of P. canaliculata could have been driven by climate changes and human activities. Notably, we identified a set of genes associated with low temperature adaptation, including Csde1, a cold shock protein coding gene. Further RNA-seq analysis and RT-qPCR experiments demonstrated the gene's dynamic pattern and biological functions during cold exposure. Moreover, both positive selection and balancing selection are likely to have contributed to the rapid environmental adaptation of P. canaliculata populations. In particular, genes associated with energy metabolism and stress response were undergoing positive selection, while a large number of immune-related genes such as Fucolectin showed strong signatures of balancing selection. Our study has advanced our understanding of the evolution of P. canaliculata and has provided a valuable resource concerning an invasive species.
**Corresponding Author:**	Wei Hu<br>Fudan University School of Life Sciences<br>Shanghai, Shanghai CHINA
**Corresponding Author Secondary Information:**	
**Corresponding Author's Institution:**	Fudan University School of Life Sciences
**Corresponding Author's Secondary Institution:**	
**First Author:**	Yan Lu
**First Author Secondary Information:**	
**Order of Authors:**	Yan Lu
	Fang Luo
	An Zhou
	Cun Yi
	Hao Chen
	Jian Li
	Yunhai Guo
	Yuxiang Xie

Powered by Editorial Manager® and ProduXion Manager® from Aries Systems Corporation

	Wei Zhang
	Datao Lin
	Yaming Yang
	Zhongdao Wu
	Yi Zhang
	Shuhua Xu
	Wei Hu
**Order of Authors Secondary Information:**	
**Response to Reviewers:**	Point-to-point response to the reviewers' comments<br><br>Response to the comments of Reviewer #1<br>Reviewer #1: The authors have made admirable improvements to the manuscript, in particular the comparisons between Pcan_SH and Pcan_SZ. However, there is still a fatal flaw in the balancing selection analysis. The authors state that an orthofinder search was employed to find duplicated genes. But this does not address the issue I raised earlier. My concern is that there are hidden paralogs that have not been assembled into to genome, which would probably be missed by orthofinder, especially if the duplications are recent and unique to this species. The fact remains that Figure 5e is is just biologically impossible, and this will be readily apparent to any reader with a basic understanding of population genetics. You simply can't have a set of biallelic SNPs with heterozygosity of 1 in a large number of diploid samples. And Figure 5e is just the gene they happen to highlight; they report a similar pattern in several genes. The only explanation is misaligned reads. Their balancing selection analysis is confounded by these artifactual genotypes. It is unacceptable to publish this balancing selection analysis without first correcting or excluding these artifacts. The authors took steps to remove SNPs in low-mappability regions or with lots of missing data, which is fine, but that doesn't get at the main problem. Hardy-Weinberg filtering is required. Even a very stringent Hardy-Weinberg filter, allowing for minor deviations due to population structure and selection, would still remove the egregious cases of SNPs showing heterozygosity > 0.8 among over 100 samples. I cannot approve the manuscript as is.<br>Response: Thank you very much for your insightful suggestion and instruction. We agreed that we could not fully exclude the possibility of the hidden paralogs that have not been assembled into to genome, indeed, which could probably be missed by orthofinder. Accordingly, we carefully reconsidered this issue and agree with you that the SNPs filtering based on the unique mapping ratio indeed cannot solve the problem. In particular, suppose the duplications are recent and the genome duplication occurred, we might have missed assembly of these regions accurately. A set of biallelic SNPs with heterozygosity of 1 might be the result of misaligned reads.<br>Therefore, we applied some more stringent criteria to address this problem. In particular, we treated the genotypes as missing data when the proportion of reads that uniquely mapped was below 80%. Furthermore, following the method recommended by Heng Li (https://github.com/lh3/psmc), we retained the genotype only when the depth of variants was at least one-third of the average read depth and no more than two-fold of the average read depth. By applying the sequence depth, we can filter out the SNPs located in duplicated regions if their sequence depth is greater than two-fold of the average read depth. Similarly, we can filter out the SNPs located in depletion regions if their sequence depth is less than one-third of the sample average depth. We also have incorporated these updated methods into our manuscript with the aforementioned information. Eventually, we have carefully examined the results the SNPs showing strong balancing selection do not exhibit extremely high observed heterozygosity, as the reviewer anticipated. In particular, 99.9% of the SNPs in our reported data are in heterozygosity < 0.75, which we believe are much closer to the expectation as the reviewer mentioned. The candidate genes list has partially changed and we have updated the results section (the lines 377-405) as well as the Table S9-S11.<br>We appreciate your persistence in pointing out this issue. We hope that these refinements address the concerns raised regarding the balancing selection analysis. Thank you again for helping us improve our manuscript.

Powered by Editorial Manager® and ProduXion Manager® from Aries Systems Corporation

Response to the comments of Reviewer #2
Reviewer #2: The authors have made new analysis and answered the questions, although the questions about sample information and gene function are in my opinion not perfect.
Response: Thank you again for your feedback on our manuscript. We appreciate your acknowledgment of the improvements we've made in response to your suggestions. We have carefully considered your comments regarding the sample information and gene function sections, and we are committed to making further improvements based on your feedback to ensure the manuscript meets the highest standards.

The environmental information of the sample is crucial for population research. Not similar to other naturally distributed species, the apple snail was considered an economic species for a long time and was artificially transported and spread. Simultaneously, through online transactions and express delivery networks, this dissemination has been accelerated. Therefore, discovering a snail at a location does not necessarily mean that it has existed here for a long time and has adapted to the environment. It is very important to determine whether the snails in the sampling area are stable and colonized populations. The author accurately listed the longitude and latitude of the sample locations, but after verification, it was found that these locations were located by the municipal governments of each city. So, the table in the answer is manipulated.
Response: We apologize for the sample's geographical location issue. We carefully checked the records during the sample collection and now we can clarify that all samples were collected in rivers, rice fields, and ponds, NOT in Municipal Government buildings which were incorrectly marked by one of the authors who was not aware of the difference between the exact sample location and the rough place representing a province or a city. The reason was that the author assumed the coordinates for a sample were just a label to reflect the information of a province or a city because our comparative analyses were at a scale of a province or a city. Please find the revised table S2.
In addition, the sample location information was obtained from the original record in the National Parasitic Resource Center (https://www.tdrc.org.cn/, Chen et al., Establishment and application of the National Parasitic Resource Center (NPRC) in China, Adv Parasitol. 2020:110:373-400), which is a first-level platform under the Basic Condition Platform Center of the Ministry of Science and Technology of China. We just took the ORIGINAL sample information from NPRC (www.portal.tdrc.org.cn). The samples in one site were selected from close or surrounding places to ensure the temperature remained consistent within a certain range. It is a routine sample collection strategy not only in population genomics analysis to investigate the genetic variation at a population level rather than the individual level but also in the P. canaliculata populations study in responses to cold acclimation (Qin Z et al., Survivorship of geographic P. canaliculata populations in responses to cold acclimation. Ecol Evol. 2020;10 8:3715-26). We hope the revised table S2 with the location information will address the reviewer’s concern.

Unfortunately, this sampling location information does not help explain the samples in some areas, making them appear more suspicious. For example, JS, SH, and ZJ are geographically very close, but in the structure diagram, they display almost completely different patterns.
Response: Thank you for your comments. Through population genetics methods, we found no significant positive correlation between the genetic distance and geographic distance of P. canaliculata in China. This finding is consistent with the results of our PCA and structure analysis. It is an important result of our study that the spread of P. canaliculata within China is still largely influenced by human activities, resulting in extensive gene flow events.
Specifically, although JS, SH, and ZJ are geographically very close, they exhibit almost completely different patterns in the structure diagram. This further supports our conclusion. Previous literature has documented multiple introduction and invasion events of P. canaliculata in China, explaining why the genetic distances between JS, SH, and ZJ populations are vast despite their geographic proximity. These populations likely originated from different introduction and invasion events, leading to significant genetic differences.
In summary, we believe these results not only reflect the complex history of the spread

Powered by Editorial Manager® and ProduXion Manager® from Aries Systems Corporation

	and dispersion of P. canaliculata but also highlight the critical role of human activities in its distribution.<br><br>Another issue is gene function. We have noticed that the author did not use the genome and genes from NCBI Refseq. But rather reassembled and annotated a new version of the genome. In the method of functional prediction, the author mentions "using local BLASTX and BLASTN programs with an e-value of 1e-5". Using only e-value without filtering identity and coverage, these results are very rough. Partial alignments can easily lead to incorrect annotation results. For the genes screened by the author, their functions need further confirmation and more scientific methods should be used. Quantity RT-PCR mainly verifies the FPKM/TPM of the transcriptome. And it hardly helps with confirming the functionality.<br>Response: Thank you for your comments. We appreciate your attention to detail and your valuable feedback regarding our approach to gene function prediction and validation.<br>In fact, we used the re-assembled genome and gene annotation that sampled from the Shanghai city for our population analysis. Through rigorous genome quality assessment methods, we established the suitability of this genome and annotation for our population genomics study. Given that genomic resources for mollusks are relatively sparse, Pcan_SH contributes to the broader set of published mollusk genomes. Furthermore, the observed chromosomal structural variations between the Pcan_SH and Pcan_SZ genomes underscore the significance of utilizing a new reference genome for our research.<br>Regarding functional prediction method employing local BLASTX and BLASTN programs with an e-value threshold of 1e-5, we acknowledge your concerns about relying solely on e-values without considering identity and coverage thresholds. However, it's worth noting that this parameter is commonly used in various research studies for gene annotation, as demonstrated in recent publications such as "Chromosome-level genome assembly and population genomic resource to accelerate orphan crop lablab breeding" (Nature Communications, 2023) and "A chromosome-level genome assembly of radish (Raphanus sativus L.) reveals insights into genome adaptation and differential bolting regulation" (Plant Biotechnology Journal, 2023). We believe this parameter is appropriate for gene annotation, due to its widespread acceptance in the scientific community. In our original manuscript, we incorporated additional methods for gene functional annotation to address the limitations of relying solely on e-values from BLAST software, although we neglected to include this information in our original method section. We employed InterProScan for protein domain annotation, and Kyoto Encyclopedia of Genes and Genomes (KEGG) pathway and Gene Ontology (GO) annotations were updated using the eggNOG v5.0 database with specific parameters ("--evalue 0.001 --score 60 --pident 40 --query_cover 20 --subject_cover 20"). These additional methods provide complementary information and enhance the robustness of our gene function predictions.<br>For the genes we highlighted by positive selection or balancing selection methods, we conducted thorough gene annotation validation by integrating multiple software tools such as BLAST, eggNOG, and InterProScan. For instance, for the gene CSDE1, we identified high sequence identity with known CSDE1 proteins in databases such as SwissProt and eggNOG, as well as the presence of a 'Cold-shock' DNA-binding domain predicted by InterProScan. Furthermore, quantitative RT-PCR confirmed a significant increase in CSDE1 expression in response to cold exposure, supporting its potential role in the cold-shock response. These findings provide valuable insights into the potential function of CSDE1 in cold response. Actually, both CRISPR-Cas9 and RNAi are reliable experimental approaches for examining and validating the gene functions. None of these techniques, however, can be used successfully on the apple snails so far. In this work, we identified a set of putative genes with the goal of offering hints regarding their potential functions for future biological investigations, as well as to provide a valuable resource concerning the invasive species.<br>Thank you once again for your valuable input and we look forward to incorporating your suggestions to improve our manuscript.
**Additional Information:**	
**Question**	**Response**
Are you submitting this manuscript to a special series or article collection?	No

**Experimental design and statistics**<br><br>Full details of the experimental design and statistical methods used should be given in the Methods section, as detailed in our Minimum Standards Reporting Checklist. Information essential to interpreting the data presented should be made available in the figure legends.<br><br>Have you included all the information requested in your manuscript?	Yes
**Resources**<br><br>A description of all resources used, including antibodies, cell lines, animals and software tools, with enough information to allow them to be uniquely identified, should be included in the Methods section. Authors are strongly encouraged to cite Research Resource Identifiers (RRIDs) for antibodies, model organisms and tools, where possible.<br><br>Have you included the information requested as detailed in our Minimum Standards Reporting Checklist?	Yes
**Availability of data and materials**<br><br>All datasets and code on which the conclusions of the paper rely must be either included in your submission or deposited in publicly available repositories (where available and ethically appropriate), referencing such data using a unique identifier in the references and in the “Availability of Data and Materials” section of your manuscript.<br><br>Have you have met the above requirement as detailed in our Minimum Standards Reporting Checklist?	Yes

Powered by Editorial Manager® and ProduXion Manager® from Aries Systems Corporation

Manuscript
Click here to access/download;Manuscript;Manuscript PC_Giga Aug7.docx

Whole-genome sequencing of the invasive golden apple snail *Pomacea canaliculata* from Asia reveals rapid expansion and adaptive evolution

Yan Lu[1,2§*], Fang Luo[1§], An Zhou[1,2], Cun Yi[1,3], Hao Chen[4], Jian Li[5], Yunhai Guo[6], Yuxiang Xie[1,3], Wei Zhang[1,3], Datao Lin[7], Yaming Yang[8], Zhongdao Wu[7], Yi Zhang[6], Shuhua Xu[1,2], Wei Hu[1,3,9*]

[1]State Key Laboratory of Genetic Engineering, Collaborative Innovation Center of Genetics and Development, School of Life Sciences, Fudan University, Shanghai 200438, China

[2]Center for Evolutionary Biology, Ministry of Education Key Laboratory of Contemporary Anthropology, Fudan University, Shanghai 200438, China

[3]Joint Research Laboratory of Genetics and Ecology on Parasite-host Interaction, Chinese Center for Disease Control and Prevention & Fudan University, Shanghai 200438, China

[4]Key Laboratory of Computational Biology, Shanghai Institute of Nutrition and Health, University of Chinese Academy of Sciences, Chinese Academy of Sciences, Shanghai 200031, China

[5]China Basic Medical College, Guangxi Traditional Chinese Medical University, Nanning 530005, China

[6]National Institute of Parasitic Diseases, Chinese Center for Disease Control and Prevention (Chinese Center for Tropical Diseases Research); NHC Key Laboratory of Parasite and Vector Biology; WHO Collaborating Centre for Tropical Diseases; National Center for International Research on Tropical Diseases, Shanghai 200025, China

[7]Zhongshan School of Medicine, Sun Yat-sen University, Guangzhou 510080, China

[8]Yunnan Institute of Parasitic Diseases, Pu'er 665000, Yunnan, China.

[9]College of Life Sciences, Inner Mongolia University, Hohhot 010070, China

---

*Correspondence: huw@fudan.edu.cn (H.W.), lueyan@fudan.edu.cn (Y.L.)

§These authors contributed equally to this work

Yan Lu [0000-0001-6907-0443]; Fang Luo; An Zhou; Cun Yi; Hao Chen; Jian Li [0000000159070925];

Yunhai Guo [0000-0001-6355-3727]; Yuxiang Xie; Wei Zhang; Datao Lin; Yaming Yang [0000-0003-4599-0397]; Zhongdao Wu [0000-0001-5879-9757]; Yi Zhang [0000-0002-3169-1823]; Shuhua Xu [0000-0002-1975-1002]; Wei Hu [0000-0002-4432-5400].

Abstract

Pomacea canaliculata, an invasive species native to South America, is recognized for its broad geographic distribution and adaptability to a variety of ecological conditions. The details concerning the evolution and adaptation of *P. canaliculate* remain unclear due to a lack of whole-genome re-sequencing data. We examined 173 *P. canaliculata* genomes representing 17 geographic populations in East and Southeast Asia. Interestingly, *P. canaliculata* showed a higher level of genetic diversity than other mollusks, and our analysis suggested that the dispersal of *P. canaliculata* could have been driven by climate changes and human activities. Notably, we identified a set of genes associated with low temperature adaptation, including *Csde1*, a cold shock protein coding gene. Further RNA-seq analysis and RT-qPCR experiments demonstrated the gene's dynamic pattern and biological functions during cold exposure. Moreover, both positive selection and balancing selection are likely to have contributed to the rapid environmental adaptation of *P. canaliculata* populations. In particular, genes associated with energy metabolism and stress response were undergoing positive selection, while a large number of immune-related genes showed strong signatures of balancing selection. Our study has advanced our understanding of the evolution of *P. canaliculata* and has provided a valuable resource concerning an invasive species.

Introduction

Pomacea canaliculata (NCBI:txid400727), commonly known as the golden apple snail, is a species of freshwater snail that originated in South America. As an invasive species, it was recently introduced to Asia as a commercial venture where it has become a serious pest of aquatic crops and rice[1]. *P. canaliculata* is listed among the 100 World's Worst Invasive Species[2]. This species has become a widely distributed agricultural and environmental pest in southern China since its introduction in the 1980s[3]. *P. canaliculata* stands out among mollusks due to its wide geographic range and its ability to survive in a variety of ecological conditions. At present, rapid growth and expansion with high population densities have disturbed the local ecological balance and caused significant losses in many countries[4]. *P. canaliculata* is also a severe threat to human health in a number of areas, as it serves as a vector for a number of parasites that cause human diseases[5]. The snail acts as an intermediate host for the pathogen *Angiostrongylus cantonensis* that can infect humans and cause potentially fatal eosinophilic meningitis[6, 7].

P. canaliculata is thought to have experienced multiple origins based on the genetic study of mitochondrial cytochrome oxidase subunit 1 (COI) gene sequences [3, 8]. It has established natural populations in most of southern China, but none in the northern China[3]. Geographical barriers are an important factor governing distribution patterns of native species. Human factors, however, were also likely to have been drivers of its invasion. *P.*

canaliculata is highly adaptable, with tolerance to a variety of ecological environments as well as pathogen invasion. The recent successful range expansion of *P. canaliculata* provides a convenient system for studying the genetic diversity and the signature of rapid microevolution, particularly genetic mechanisms related to rapid local adaptation to novel environmental conditions in a short period of time. In addition, environmental factors such as temperature and pathogen load have influenced the distribution range of *P. canaliculata[9]*. Temperature may be a key environmental factor restricting the migration of *P. canaliculata[10]*. The ability to survive at low temperature constitutes a critical factor for successful range expansion of *P. canaliculata* in temperate East Asia as well as tropical Southeast Asia[11]. It has been suggested that low temperature in winter is a limiting factor in the geographic expansion and successful establishment of apple snail populations[12]. Previous study has shown that the expression of glycerol kinase (*GK*), heat shock protein 70 (*HSP70*), Na+/K+-ATPase (*NKA*), and glycerol-3-phosphate dehydrogenase (*GPDH*) genes is related to the cold hardiness of *P. canaliculate*[13, 14]. Transcriptome sequencing revealed that candidate cold-resistance genes were related to glucose metabolism pathway. The lncRNA of *P. canaliculata* could participate in cold acclimation by regulating the expression of E3 ubiquitin protein ligase, 26S proteasome non-ATPase dependent regulation subunit, glutathione S-transferase, sodium/glucose cotransporter and cytochrome *P450*[15]. However, the genetic mechanism of

low temperature adaptation in *P. canaliculata* has not yet been investigated based on a large scale of whole-genome sequencing data, particularly at the population genetic level.

Despite the increasing biological and economic impacts of this invasive species, little is known about the evolutionary processes that underlies the geographic range expansion and adaptive evolution of invasiveness of *P. canaliculata*. In this study, we assembled a chromosomal-level reference genome from an adult female *P. canaliculata* that was collected from Shanghai, China, and we investigated the population structure, demographic history, genetic diversity, and local adaptation of *P. canaliculata* by sequencing and analyzing 173 whole genomes covering most of the current range of distribution in Asia. Our study revealed that *P. canaliculata* populations in Asia have undergone multiple episodes of rapid expansion that may have been driven by human factors. Furthermore, we identified a set of genes that may be involved in the adaptive invasion, particularly concerning adaptation to low temperatures. Additionally, balancing selection is likely to have contributed to the rapid environmental adaptation of *P. canaliculata* populations in Asia. Our findings provide insights into the genomic mechanisms of this invasive species that underlie the rapid local adaptation to novel ecological environments.

Results

A New reference genome for *P. canaliculata*

We assembled the *P. canaliculata* genome collected from Shanghai city,

China by incorporating high coverage of PacBio CLR and high-throughput chromatin conformation capture (Hi-C) technologies. The PacBio reads were *de novo* assembled into contigs, followed by polishing with both PacBio and Illumina reads. This resulted in an assembly of 2235 contigs with a N50 length of 1.16 Mb (supplementary table 1). A total of 434 million Hi-C read pairs were generated to scaffold the assembled contigs. Finally, we obtained a *P. canaliculata* reference genome (Pcan_SH) with scaffolds N50 of 31.4 Mb and genome length of 440.8 Mb. Notably, 432.4 Mb (98.11%) of sequence was anchored to 14 pseudochromosomes, which is similar with the published genome of *P. canaliculata* (Pcan_SZ, NCBI Accession: GCF_003073045)[16] (supplementary table s1, supplementary fig. S1). 24,832 protein-coding genes were predicted, with over 91.81% of these genes being functionally annotated using the public databases (supplementary table s1).

Genome comparative analysis between the *P. canaliculata* genomes (Pcan_SH and Pcan_SZ) revealed intriguing insights. Hi-C interaction heatmaps for the Pcan_SH assembly displayed minimal inter-chromosomal interactions, contrasting with noticeable off-diagonal interactions in Pcan_SZ (supplementary fig. S2). Alignment of the genomes of Pcan_SH and Pcan_SZ showed good collinearity between the two reference genomes (supplementary fig. S3). Despite the high collinearity, we identified a total of 95 inversion, 1242 translocations events. These chromosomal rearrangements were further supported by the high-density contacts in Hi-C heatmaps generated from

Pcan_SZ Hi-C reads aligned to the Pcan_SZ genome, while no off-diagonal interactions were visible in Pcan_SH (supplementary fig. S4). These results suggested a more precise and accurate assembly process for Pcan_SH genome.

Population structure and demographic history

After quality control and filtration for genetic relatedness, 130 *P. canaliculata* genomes from East and Southeast Asia were retained for further analysis, with an additional genome from South America (Argentina). Using Pcan_SH as reference genome, we identified a total of 13.55 million SNPs with an average 14.7 × depth (fig. 1a, supplementary fig. S5, table S2-S3). Principal component analysis (PCA) revealed that East Asia (EA) and Southeast Asia (SEA) samples were divided into two distinct subclades in the two-dimensional PC plot, indicating a regional distribution pattern during the invasion. Samples from Shanghai (SH) and Zhejiang (ZJ) were grouped together in a sub-cluster of the EA populations, samples from YNSM, HN, GX, JS, FJ and GDZQ were clustered together (EA_solo), while the remaining samples were scattered in a different cluster (EA_mix). Within the EA subclades, sampling locations did not discretely cluster along these PC axes, instead, we discovered that most EA populations, with the exception of SH and ZJ, maintained consistency with one another in PC2 but exhibited a continuous genetic structure in PC1 (fig. 1b). Interestingly, there were no obvious sub-clusters reflected by most EA samples and the resulting plots did not correspond to their geographic locations, possibly

due to the multiple migrations and genetic interactions. Using the *P. maculate* genome as the outgroup, a maximum likelihood (ML) phylogenetic tree produced the same findings as the PCA. Samples from diverse geographical locations were classified into separate clades (fig. 1c). Besides, SH and ZJ are near to the Argentina sample in ML tree, suggesting a closer genetic affinity to the country of origin.

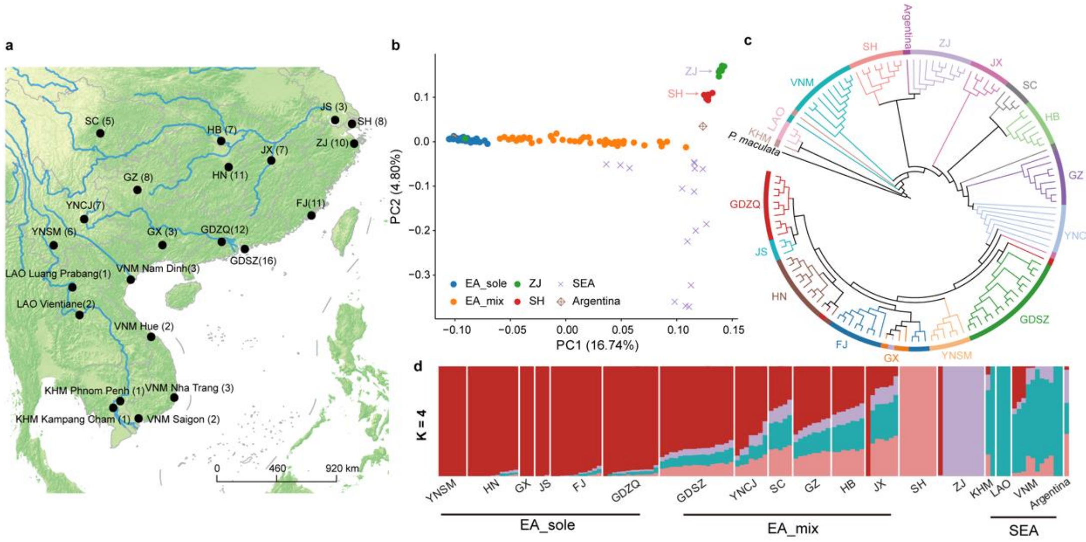

FIG 1. Sampling locations and population structure of *P. canaliculata*. a, Geographic distribution of *P. canaliculata* samples. **b,** Principal component analysis (PCA) plot showing segregation of the *P. canaliculata* individuals. The proportions of the variance explained were 16.74% by PC1 and 4.80% by PC2. Each point is colored according to where the sample was collected. **c,** Maximum likelihood (ML) phylogenetic tree of the *P. canaliculata* individuals with 1000 nonparametric bootstrap replications. *Pomacea maculate* was selected as the outgroup. **d,** Ancestry results from Admixture analysis under the best K = 4 model supported by an examination of cross-validation errors. Each color represents a different ancestry composition.

The population structure of *P. canaliculata* was further inferred by individual ancestry coefficients. We estimated 4 as the most likely number of ancestral populations based on the estimation of cross-validation (CV) error (supplementary fig. S6). Assuming K = 4, we found that the proportions of genetic components differed between EA and SEA populations. Interestingly, SH and ZJ samples shared their otherwise unique components, suggesting that there were limited genetic effects from other areas. East Asian and Southeast Asian components were present in six locations, SC, GDSZ, YNCJ, GZ, HB, and JX, indicating multiple population interactions or invasions (EA_mix). Notably, the HN, GX, JS, FJ, and GDZQ populations (EA_solo) barely shared genetic components with the SEA population (fig. 1d). These results corroborated previous studies using mtDNA COI sequences[3] and supported the possibility of multiple invasions of Asia by showing varying degrees of migration and genetic interactions.

Genomic diversity and genetic relationships

We estimated the genome-wide median nucleotide diversity (π) in populations of *P. canaliculata* and other molluscan species. The nucleotide diversity in *P. canaliculata* populations (range from 0.00427 to 0.0580; supplementary fig. S7) was comparable but significantly greater than previously published molluscan data, with the exception of another invasive species, *Crassostrea gigas* (fig. 2a). Given the link between genetic diversity and ecological resilience[17], it stands to reason that *P. canaliculata* would have a

higher level of genetic diversity than other mollusks. Due to the strong intrinsic link between linkage disequilibrium (LD) decay and genetic diversity, we then estimated the pairwise LD (r^2) with all high-quality SNPs in *P. canaliculata* populations. As expected, the r^2 value declined with the increasing physical distance between SNPs. The distance with r^2 reaching half of its maximum value occurred at ~30 bp across all snail populations (supplementary fig. S8). Genome-wide Tajima's *D* estimates were positive for all populations, indicating an excess of intermediate-frequency polymorphism as a consequence of population contraction or balancing selection (fig. 2b). Additionally, we found greater SNP differentiation among populations in the EA and SEA clades (range of F_{ST} = 0.0936–0.64382) than within the EA clades, with the exception of the SH and ZJ populations (range of F_{ST} = 0.0165–0.14833), suggesting a pattern of rapid radiation in the EA clades (supplementary table S4). It is noteworthy that no significant correlation was observed between genetic distance (F_{ST}/(1-F_{ST})) and geographical distance (great circle distance) in EA populations (R = 0.2046, P = 0.0758, fig. 2c), indicating that human activity was involved during invasion events.

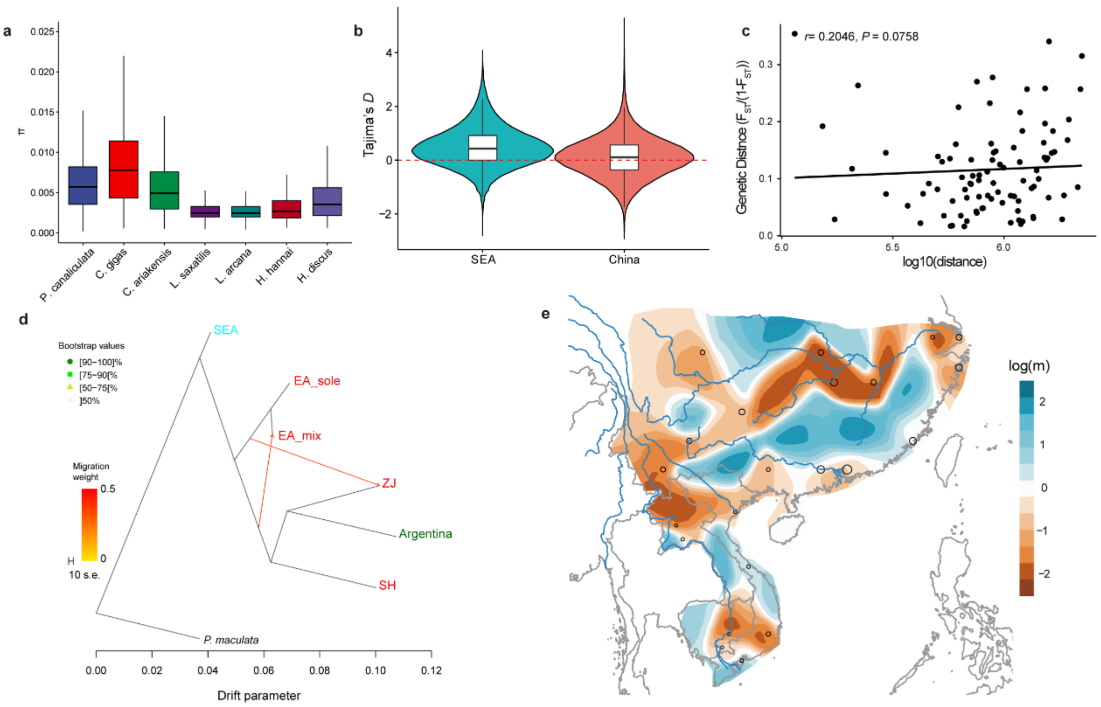

FIG 2. Genomic diversity and population migration among the *P. canaliculata* populations. a, Estimates of genome-wide nucleotide diversity (π) in *P. canaliculata* and molluscan species with accessible whole-genome data are compared. **b,** Tajima's *D* calculated for each population. The violin plots show the kernel probability density of the data; the box represents the interquartile range, and the horizontal marker represents the median of the data. **c,** Relationship between genetic distance ($F_{ST}/(1-F_{ST})$) and geographical distance for all sampled populations in East Asia (EA). The Spearman's correlation coefficient (ρ) and the P value estimated using a Mantel test with 1000 permutations are shown. **d,** TreeMix-inferred population tree with seven migration edges (*M*=2). Migration events are indicated by arrows and are colored according to the migration weight. Bootstrap support is indicated for each of the nodes. **e,** Effective migration (gene flow) surfaces estimated in EEMS for *P. canaliculata*. Color bars show the effective migration rate on a $\log_{10}$ scale relative to the average migration rate over the entire range. The darker blue indicates areas with stronger

gene flow, whereas darker orange depicts areas with lower gene flow. The sizes of the black circles represent the number of sampled individuals in a given locality.

To explore the evolutionary relationships among populations and potential admixture events, we employed TreeMix and outgroup *f*3 to test for relatedness of different *P. canaliculata* populations. With the exceptions of ZJ and SH, all of the internal branch lengths in the EA clades were relatively short, and the TreeMix analysis detected frequent signals of gene flow among EA populations, a result that was consistent with populations that were rapidly spreading (fig. 2d, supplementary fig. S9 and fig. S10). Hybridization also likely occurred between SEA and EA populations. Taking *P. maculata* as an outgroup, the Argentina sample showed higher *f*3 values with EA populations than with SEA populations. ZJ and SH had a stronger affinity with Argentina. When X belonged to EA populations, the target population VNM had higher *f*3 values, indicating that it shared more genetic components with EA populations (supplementary fig. S11).

We then used EEMS analysis of EA populations to identify a distinct genetic barrier that runs roughly parallel to the Yangzi River Basin in China. It has been reported that *P. canaliculata* is predominantly found south of the Yangzi River Basin due to the ambient temperature. Founder events crossing the river and harsh environment during range expansion involved fewer individuals, leading to greater genetic drift between populations from northern and southern China. Moreover, the barriers in SEA populations coincided with three international

boundaries (China-Laos, Laos-Vietnam, and Vietnam-Cambodia), further suggested that human activity or cross-border trade possibly have been major factors in the invasion of *P. canaliculata* (fig. 2e and supplementary fig. S12).

Genomic signatures of low-temperature adaptation

With the aid of human activity, populations of *P. canaliculata* have successfully invaded Asia within a relatively short time. The identification of genomic signatures that are consistently linked to invasion success has been made possible by these replicated invasion events. The most important factor driving the invasion of *P. canaliculata* is considered to be the environmental temperature, although many other variables, including the level of dissolved oxygen, the pH of the water, and soil moisture during dormancy, are associated with overwintering success[18]. Numerous studies have revealed that low temperature in winter is a limiting factor in the geographic expansion and successful establishment of apple snail populations[12, 19, 20]. Given the significant differences in temperature between East and Southeast Asia, as well as the different population structures inferred from the PCA, we used the BayPass software to conduct a genome-wide scan to identify genes involved in adaptation during invasion, with the Min Temperature of the Coldest Month (Bio06) selected as the primary environmental factor (See Methods). In total, 648 outlier SNPs with a Bayes Factor (BF) greater than 20 were discovered, and 436 linked genes were annotated (Supplementary fig. S13). We analyzed the gene ontology (GO) annotation of these genes (*P*-value < 0.05; See

Methods) and found them to be clustered into five interacting networks that were linked to the functions of circadian sleep/wake cycle, associative chemosensory locomotory learning, circulatory circulation muscle contraction, negative action involved migration, and axonogenesis branching disc differentiation (supplementary fig. S14).

In particular, we identified a number of genes such as *TRHR* and *CSDE1* that covered outlier SNPs highly relevant to temperature (Supplementary Fig. S13 and table S5). As a member of the G protein-coupled seven-transmembrane domain receptor superfamily, *TRHR* encodes a central thyrotropin-releasing hormone (TRH) receptor. The TRH system is known to be involved in thermoregulation and glucose metabolism, two important adaptive systems functioning during cold exposure[21]. Animals with TRH deficiency exhibit impaired cold tolerance and glucose metabolism[22, 23]. In addition, the Cold Shock Domain Containing E1 (*CSDE1*) gene, also known as Upstream of N-Ras (UNR), codes for an RNA-binding protein (RBP) that has five cold-shock domains (CSDs). The cold-shock protein plays an important role in stress adaptation and low temperature tolerance, functions that are well characterized in bacteria and plants[24, 25]. Notably, we discovered eight SNPs at the 5'-UTR regions of the *CSDE1* gene that were highly relevant to temperature (fig. 3a). The post transcriptional regulation of *CSDE1[26]* may be affected by these outlier SNPs in the 5'-UTR regions, which would further contribute to the cold adaptation. The median-joining network analysis revealed twelve haplotypes

were clustered into two clades and samples from LT regions are predominantly enriched in clade 1 (fig. 3b, supplementary fig. S15). Moreover, we observed that an alternative allele (Chr7: g. 27642529 A>G) with the highest BF value within the *CSDE1* gene was strongly positively correlated with temperature (ρ = 0.518, P = 0.023) (fig. 3c, d). Furthermore, we found that *CSDE1* was highly expressed in several tissues of *P. canaliculata,* especially in the hemocytes, ovary, and testis (fig. 3e). To further investigate the dynamic expression of *CSDE1* in response to exposure to cold, we also carried out a RT-qPCR experiment. We found that within the first 24 hours of exposure to the cold, the expression of *CSDE1* in the hemocytes dramatically increased (fig. 3f) and then rapidly declined throughout the following 4 days. These findings provided evidence for the potential role of *CSDE1* in the cold-shock response.

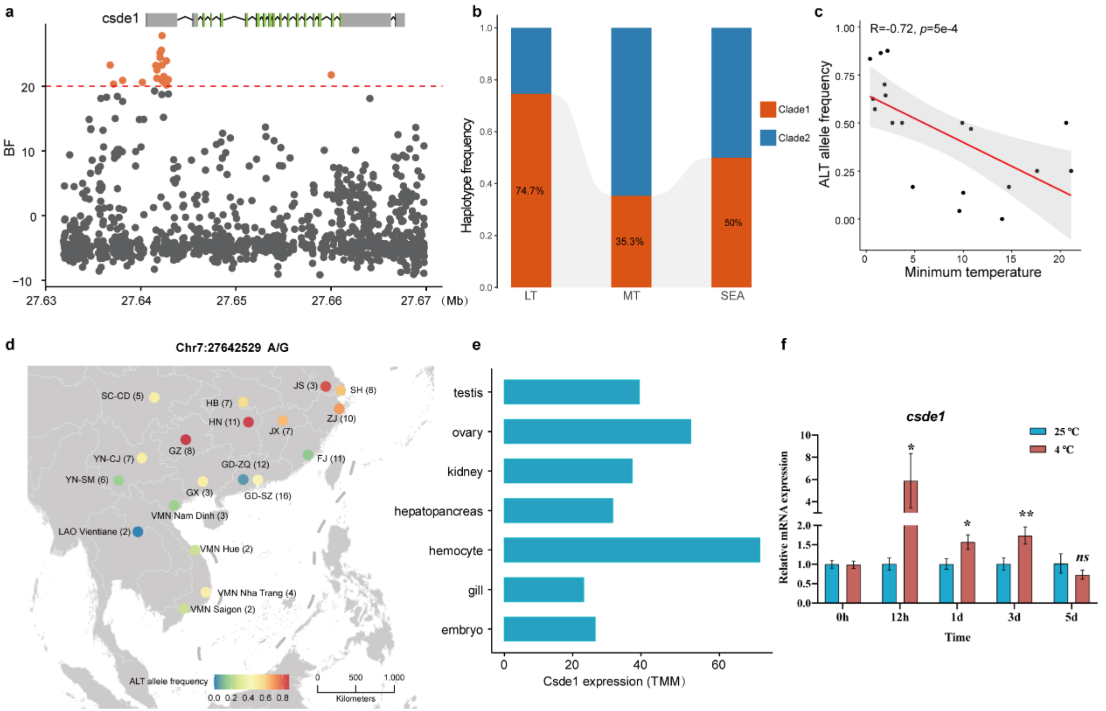

FIG 3. Genotype-environment association for the Min Temperature of the Coldest Month

in different sampling locations on the *CSDE1* gene. a, Bayes factor (BF) value in the *CSDE1* region. The horizontal red dashed line (BF > 20 dB) corresponds to the chosen significance level for genotype-climate association. **b,** Haplotype frequency in the *CDSE1* 5'-UTR region for different types of *Pomacea. canaliculata* accessions, LT: individuals from East Asia where the minimum temperature is below 2 °C; MT: individuals from East Asia, except for LT individuals; SEA: individuals from Southeast Asia. **c,** Significant negative correlation between the alternate allele frequency of the SNP (Chr7: 27642529 A/G) and the Min Temperature of the Coldest Month in different sampling locations. **d,** Alternate allele frequency of the SNP (Chr7: 27642529 A/G) in the EA and SEA populations. **e,** Expression level of *CSDE1* in different tissues of *P. canaliculata*. **f,** RT-qPCR validation for *CSDE1* performed on hemocyte tissue of *P. canaliculata*, with four replicates at 4°C and 25°C (* $P < 0.05$, ** $P < 0.01$, **** $P < 0.0001$ by Welch's *t*-test).

As BayPass is an environmental association analysis (EAA) for identifying subtle shifts in allele frequency associated with local adaptation[27], the programs have difficulty in detecting selective sweeps unique to one or few populations or sweeps concerning different haplotypes associated with the same gene. Therefore, we further investigated the genomic signature for different environments with standard genome-wide scan approaches. We performed selective sweep analyses (F_{ST}, iHS, and XPEHH, see Methods) to identify candidate genes involved in cold adaptation in the comparisons between Low Temperatures populations (LT, all individuals from EA where the minimum temperature is below 2°C) and High Temperatures populations (HT, all individuals from the SEA population). Overall, we identified 750 non-

redundant regions (total length = 7.04 Mb) that exhibited at least two extreme scores of Fst, iHS, or XPEHH, encompassing 754 genes (representing 3.33% of all coding genes) (Fig. 4a, supplementary table S6). Several genes bearing signals of positive selection in the LT population were associated with glycolysis (e.g., *Fbp1*, *AGL*, and *PKM*), in mediating the uptake of glucose (e.g., *Slc2A3*, *Slc2A13*, and *Slc2A1*), and in stress response (e.g., *ITPR1*, *PRRC2C*, *CREBBP*, and *D2R*). Functional analysis showed that these selected genes were significantly enriched for GO terms related to positive regulation of transporter activity (GO: 0032411, *p*-value=3.51 × 10^{-5}), regulation of skeletal muscle contraction (GO:0014819, *p*-value=3.94 × 10^{-5}), and regulation of calcium-mediated signaling (GO:0050848, *P*-value=5.55 × 10^{-5}) (supplementary table S7). It is notable that the *Sqrdl* gene encoding sulfide quinone oxidoreductase showed strong positive selection in the LT population supported by the elevated iHS, F_{ST}, and XPEHH values (fig. 4a, e). *Sqrdl* plays a key role in controlling H_2S availability via oxidation for inhibiting mitochondrial respiration, thereby reducing energy during torpor or hibernation to respond to the cold stress[28]. A significantly lower Tajima's *D* statistic and nucleotide diversity (π) were observed in the all LT individuals compared to the SEA populations (fig. 4c, d), further supporting the hypothesis of positive selection in the LT population. Notably, one nonsynonymous variant (Chr2: g. 23398320) in the *Sqrdl* gene exhibited extreme XPEHH (normalized XPEHH = 3.80736) and F_{ST} (F_{ST} = 0.498969) values (fig. 4e) and had a pronounced signature of

natural selection (fig. 4f). RNA-Seq[16] further supported *Sqrdl* being significantly upregulated under cold stress (foldchange=1.4, *p*-adjust= 7.87e^{-05}), pointing to a functional role of *Sqrdl* for cold adaptation (fig. 4b).

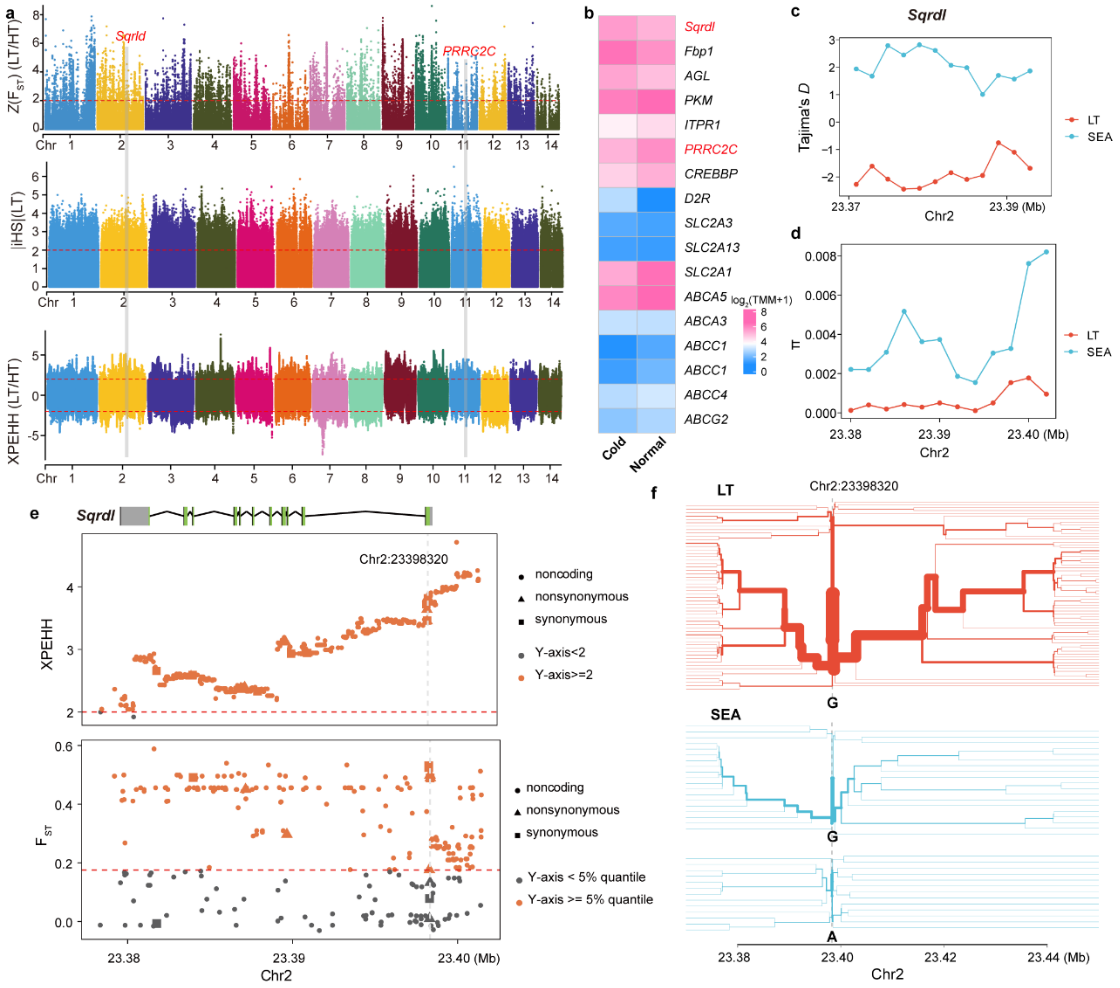

FIG 4. Positive selection scans for low-temperature adaptation in the LT populations of *P. canaliculata*. a, Whole genome scan with F_{ST}, iHS, and XPEHH. F_{ST} is normalized as *z* scores for the *P. canaliculata* genome. The horizontal red dashed lines represent the empirical threshold for the selected regions. F_{ST}: top 5% windows; iHS and XPEHH: 2. **b,** Expression level of the positively selected genes under cold stress. Nucleotide diversity (**c**) and Tajima's *D* statistic (**d**) in the *Sqrdl* gene for LT and SEA populations. **e,** Multiple statistics indicating positive

selection on the genomic region harboring the *Sqrdl* gene. The y-axis represents the normalized XPEHH (the first panel) and F_{ST} values (the second panel). Circles, triangles, and squares denote non-coding, non-synonymous, and synonymous variants, respectively. **f,** Haplotype bifurcation plots for LT and SEA haplotypes across Chromosome 2 positions from 23.28 to 23.44 Mb. The colors of each plot reflect the location of sampling. Labels indicate nucleotides at the central position. LT: all individuals from East Asia where the minimum temperature is below 2°C; SEA: all individuals from Southeast Asia.

Interestingly, we found that nine genes showing selective sweep signatures that were also identified in the BayPass analysis as being associated with temperature (supplementary table S8). In particular, the *PRRC2C* gene, which is involved in the formation of stress granules (SGs)[29], was focused on because strong selection signals were detected using all three of the above methods (supplementary fig. S16a). In addition, a significantly lower Tajima's *D* statistic and nucleotide diversity (π) were observed in the LT population (supplementary fig. S16b and S16c), and significant differences between LT and SEA populations were observed in the extended haplotype homozygosity of the peak SNPs. These findings suggested that *PRRC2C* has undergone positive selection (supplementary fig. S16d, and S16e). *PRRC2C* was significantly differentially expressed after exposure to cold according to the RNA-Seq data (foldchange=0.62, *p*-adjust=, $6.92e^{-09}$, fig. 4b, supplementary Table S7). Remarkably, we observed that one variant located in the 5'-UTR (Chr11: g. 16598124) and one nonsynonymous variant (Chr11: g. 16572768)

showed highly divergent frequencies between LT (98.47%) and SEA populations (53.12%; supplementary fig. S16a, S16f). The allele frequencies of these two *PRRC2C* gene variants were strongly positively correlated with temperature, suggesting that they may contribute to cold adaptation in the LT population (supplementary fig. S16g).

Balancing selection contributed to the adaptive invasion

When invasive species enter a new environment, population bottlenecks typically result in losses of genetic diversity. However, there are exceptions caused by other evolutionary processes that can facilitate invasion, including the maintenance of genetic diversity through balancing selection. Therefore, we searched for the genomic signatures of balancing selection using β scores and detected 1000 regions covering 1086 genes in the EA populations and 932 regions covering 1027 genes in the SEA populations using top 0.5% value as the highest significance level (fig. 5a, supplementary Table S9 and S10). The analysis revealed a high contribution from balancing selection. Notably, significant balancing selection signals were discovered in both the EA and SEA populations in a total of 235 genes, of which 199 genes showed differential expression levels in response to cold, heat, drought, or heavy metal stimulation (supplementary table S11). The overlap between balancing selection genes and differentially expressed genes was significantly higher than expected by chance (Fisher's exact test, $P < 2.2 \times 10^{-16}$). Functional analysis showed that these balancing selecting genes in both the EA and SEA populations were

highly enriched in the GO term related to response to radiation (GO:0009314, p-value= 0.000135), response to light stimulus (GO:0009416, p-value= 0.000436), larval lymph gland hemopoiesis (GO:0035167, p-value= 0.000912) and cellular response to organonitrogen compound (GO:0071417, p-value= 0.00130), which were probably associated with stress adaptation ($P < 0.05$; supplementary fig. S17). Besides, kyoto encyclopedia of genes and genomes (KEGG) pathway enrichment analysis showed these overlap genes were generally enriched in pathways such as: MAPK signaling pathway (map04010, p-value = 0.00765409) and Cytokine-cytokine receptor interaction (map04060, p-value = 0.008744156) (supplementary fig. S18), which may be associated with stress responses and immune responses [30, 31].

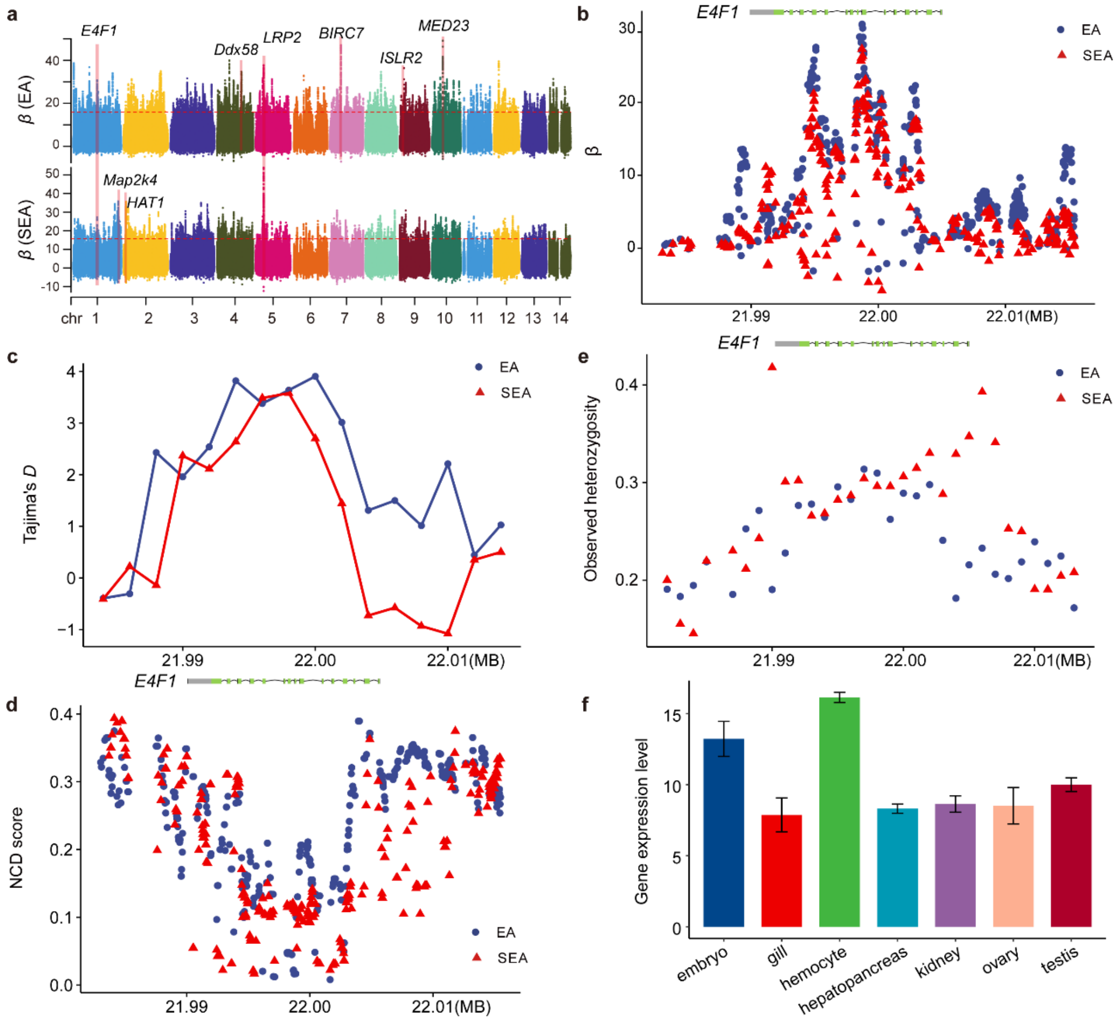

FIG 5. Balancing selection in the *P. canaliculata* population. a, Regions of balancing selection detected in the EA and SEA populations based on *β* scores. The dashed line represents the significance level of the top 0.1% *β* score values. **b,** Enlarged diagram of *β* statistics around the *E4F1* gene in the EA and SEA populations. Tajima's *D* (**c**) and NCD (**d**) statistics around the *E4F1* gene in the EA and SEA population. **e,** Diagram showing the heterozygous genotype frequency around the *E4F1* gene region, calculated as the mean of all variants in 1 kb non-overlapping windows. **f,** Expression profile of the *E4F1* gene in different tissues of *P. canaliculata* .

Furthermore, genes exhibiting significant signals of balancing selection

were found to be highly enriched in the Gene Ontology (GO) terms associated with immune modulation in various body regions(*P* < 0.05), particularly in EA populations (supplementary fig. S19). Specifically, we observed significant signals in both the EA and SA populations for the *E4F1* gene (Pca0010750), (β_{EA} = 30.73, β_{SEA} = 27.31 ; fig. 5b; supplementary table S10), a result that was validated by the Tajima's *D* and NCD statistics (fig. 5c, d). The *E4F1* gene, a transcriptional regulator, has been reported to play an important role in maintaining animal innate immune[32]. In addition, several nonsynonymous SNPs within the *E4F1* gene exhibited increased heterozygosity, indicating a strong signal of balancing selection (fig. 5e; supplementary fig S21). The transcriptome data[33] also revealed that *E4F1* was preferentially expressed in the hemocytes, which are known to play essential roles in the internal defense mechanisms of mollusks[34] (fig. 5f).We noticed that the *LRP2* genes showed signatures of balancing selection in both the EA and SEA populations (ranked as top 6 in EA and top1 in SEA; fig 5a; supplementary table S11). Although specific mechanism studies are limited in mollusc, *LRP2* may be involved in development, pathogen recognition and immune regulation processes. For example, insect defensin promotes the adsorption and infection of JEV virus through *LRP2*[35], suggesting a potential role for *LRP2* in host-pathogen interactions. Interestingly, we found that several genes exhibiting highly significant balancing selection signals were associated with immunity or stress tolerance. Theses genes includs the *med23*[36], *BIRC7*[37], *ISLR2[38]*, and

Ddx58[39] in the EA populations (ranked among the top 10 genes, fig5a; supplementary table S9), as well as the *Map2k4*[40], *HAT1* [41] in the SEA populations (ranked among the top 3 genes, fig5a; supplementary table S10). These genes were highly expressed in several tissues of *P. canaliculata,* especially in the gill, hemocytes and hepatopancreas, which are crucial tissuses of *P. canaliculata* relevant to detoxification and immunity (supplementary fig. S22). These findings illustrates that genes associated with immunity, detoxification, and stress tolerance, which were more likely to be subjected to balancing selection, contributed to the adaptive invasion of *P. canaliculata*.

Discussion

The golden apple snail *P. canaliculata* has drawn considerable attention throughout the world due to its environmental invasiveness, wide range of stress adaptations, and rapid reproduction. We performed the first whole-genome analysis of 173 representative *P. canaliculata* individuals to comprehensively understand the genetic diversity and evolutionary history of this invasive species. Our findings confirmed the multiple origins and migrations of *P. canaliculata* using data at the whole-genome level that had already been discovered using MT-DNA data[3]. *P. canaliculata* populations still possessed sub-population genetic structure, indicating that they have experienced complex genetic interactions during the short period of invasion based on our WGS data. Additionally, the genetic diversity of *P. canaliculata* was higher than

that of other mollusks, and we found higher interpopulation genetic differentiation than intrapopulation genetic differentiation, pointing to a trend of rapid radiation throughout the Asian continent. Interestingly, several significant genetic barriers coincided with the Yangzi River Basin and international borders, implying that climate and human activity may have been the major factors influencing the dispersal of the invasive golden apple snail.

We identified a genetic barrier coinciding with climate, and numerous studies have revealed that low temperature in winter is a limiting factor in the geographic expansion and successful establishment of apple snail populations[12, 19, 20]. We selected the Min Temperature of the Coldest Month as the primary environmental factor and finally identified a set of candidate genes associated with temperature. The gene that stood out the most was *Csde1*, where certain haplotypes were more prevalent in samples from low-temperature zones. Intriguingly, a variant (Chr7: g. 27642529 A>G) in *Csde1* showed the highest alternative allele frequency in the majority of low-temperature regions but was absent in high-temperature regions (Guangzhou and Laos). This finding is consistent with a previous study that reported that individuals from these low-temperature regions had the highest survival rate and prolonged survival time regardless of the temperature acclimation treatment, whereas individuals from Guangzhou were the shortest-lived[12]. Previous studies focused on the heat shock proteins (HSPs) in the invasive apple snail, which are supposed to play critical roles in how they adapt to harsh

environments[13, 42, 43] and indicated that HSPs may be related to the thermal resistance of *P. canaliculata[44]*. We made the first effort to discover the *Csde1* associated with cold resistance in *P. canaliculata* and performed a transcriptional analysis and RT-qPCR validation to illustrate its dynamic pattern during cold exposure and biological functions; the result could be a potential and powerful genetic candidate for prevention and control of the invasive species.

We comprehensively analyzed and compared the genomic selection signatures of low- and high-temperature populations using multiple methods. The genome-wide scan identified a set of genes showing significant selective sweep signals. For example, *Sqrdl* had a pronounced signature of natural selection in low-temperature populations and was highly upregulated under cold stress based on RNA-seq data[16]. Besides, we proposed an approach in which genes with strong temperature association and significant selection signals in more than two selective sweep methods were defined as being more likely to be genes and alleles involved in cold adaptation. Eventually, nine candidate genes were identified. The genes *pqn-25* and *PRRC2C* are reported to be linked to SGs[29, 45]. *Pka-C1* positively regulates cold stress[46] and plays a major role in providing cold adaptation and tolerance to freezing[47]. *Gyc32E* plays an important role in both cold and heat stress-induced pathways [48]. *Dopamine D2-like receptor* inhibits cold-initiated thermogenesis in brown adipose tissue[49]. Interestingly, RNA-seq analysis[16] revealed that *pqn-25,*

PRRC2C, Pka-C1, and *Gyc32E* were significantly downregulated under cold stress. *Dopamine D2-like receptor* was the only gene that was highly upregulated, reflecting its negative regulation function in cold adaptation.

Adaptive evolution is one of the primary mechanisms that enable organisms to endure and flourish in new environments. *P. canaliculata*, which originated in South America and recently migrated into Asia, could be an excellent model for understanding how species rapidly adapt to new environments. We found 235 genes that indicated a high contribution from balancing selection, of which 199 genes showed differential expression in response to various stimuli. The proportion was significantly higher than expected by chance. We also found that many immune-related genes in both the EA and SEA populations had significant balancing selection signals. These immune-related genes could serve as an evolutionary basis for the continuous antagonistic co-evolution between *P. canaliculata* and a wide range of pathogens in Asia. Balancing selection is a classic mechanism for maintaining variability in immune genes involved in host–pathogen interactions[50]. Overall, positive selection and balancing selection as important evolutionary forces are likely to have contributed to the rapid environmental adaptation of *P. canaliculata* populations in Asia.

Materials and Methods

Sample collection and sequencing

Individuals of *P. canaliculata* for genome assembly were collected from

Shanghai city, China. Using standard phenol/chloroform extraction, we extracted the genomic DNA of *P. canaliculata* from the foot tissue of a female individual. The integrity and concentration of gDNA were further assessed by gel electrophoresis and an Agilent Bioanalyzer 2100 (Agilent Technologies), respectively. Four paired-end libraries were constructed with insert sizes of 250 base pairs (bp), 300 bp, 500 bp, and 2 kb and then sequenced on the next-generation sequencing (NGS) Illumina Hiseq X Ten platform (RRID:SCR_016385). To generate the ultra-long genomic reads, 20 kb genomic sequencing libraries were constructed and sequenced on the third-generation sequencing (TGS) PacBio SEQUEL platform (RRID:SCR_017989), yielding more than 30 Gb of subreads with an N50 length of 5.7 kb and the longest read of 150 kb. Ten grams of gDNA was also used for Hi-C library construction using a previously described method[51], followed by sequencing on the Illumina X Ten platform in 150PE mode.

For RNA preparation and sequencing, ocular, skin, muscle, gonadal, intestinal, liver, kidney, blood, gall, and air bladder tissues of *P. canaliculata* were combined, and total RNA was extracted from 50 mg of composite samples using the TRIZOL Reagent (Invitrogen). Size selection of 0–3 kb and 2–6 kb was performed using the BluePippin Size Selection System (Pacific Biosciences of California). SMRTbell Template libraries were constructed with cDNA products using a SMRTBell Template Prep Kit, then subjected to one or two cells on the PacBio SEQUE platform (Pacific Biosciences of California). A

library with an insert length of 250 bp was also sequenced on Illumina HiSeq 2000 in the 150PE mode (Illumina Inc.).

Genome assembly of Pcan_SH and assessment

The long reads generated by the PacBio SEQUEL platform were assembled with FALCON (RRID:SCR_023199) [52] using a series of parameters. We found that the assembly size and contig N50 both increased with reducing length cut-off of self-corrected long reads used for assembly, while the assembled genome size and N50 length reached plateaus of ~560 Mb and ~280 kb, respectively, when the length cut-off was less than 6 kb. We further assembled the genome using PacBio long reads with CANU (RRID:SCR_015880) [53] and RACON (RRID:SCR_017642) [54], leading to contig N50 lengths of 196 kb and 550 kb, respectively. We then purged haplotigs and overlaps in the contigs assembled by FALCON based on the PacBio read depth using purge_dup software (RRID:SCR_021173). The final contigs were further polished by arrow[55] with TGS reads and pilon (RRID:SCR_014731) [56] with NGS reads. Chromosomal assembly of *P. canaliculata* was carried out using Hi-C data. Raw Hi-C reads were polished and filtered using hiclib as described previously[57]. Lachesis was applied to cluster the final contigs into 14 groups using the agglomerative hierarchical clustering method and was further used to order and orient the clustered contigs (Pcan_SH).

Core gene mapping ratios greater than 96% were obtained from both CEGMA

(RRID:SCR_015055) [58] and BUSCO (RRID:SCR_015008) [59], validating the completeness of the assembled genome. By mapping NGS reads to the finale genome, more than 98% of NGS short reads were mapped to the genome, and 96% were paired aligned, further confirming the correctness of the genome assembly.

Genome annotation

Tandem repeats of the *P. canaliculata* genome were detected using Tandem Repeats Finder. Transposable elements (TEs) were identified using a combination of homology-based and *de novo* approaches. RepeatModeler (RRID:SCR_015027) was used initially to generate a *de novo* repeat library that was then combined with the known repetitive sequences (e.g., RepBase 17.01). The TEs in the *P. canaliculata* genome were further identified by mapping to the library using the software RepeatMasker (RRID:SCR_012954) [60]. Finally, a total of 132.96 Mb of the sequences were identified as TEs, comprising 22.79% of the genome.

Gene prediction was performed with *de novo*, homology-based, and sequencing-based methods to annotate the *P. canaliculata* genome. We used Augustus (RRID:SCR_008417) [61] to predict coding genes via *de novo* prediction. For homology-based prediction, protein sequences were downloaded from Ensembl[62] for closely related mollusk species, including *Aplysia alifornica*, *Biomphalaria glabrata*, *Crassostrea gigas*, *Lottia gigantea*, and *Mizuhopecten yessoensis.* These sequences were aligned against the *P. canaliculata* genome

using TBLASTN software (RRID:SCR_011822) [63]. GeneWise (RRID:SCR_015054) [64] was then used to define gene models for the *P. canaliculata* genome. For the sequencing method, full-length transcriptomes from Iso-seq were first aligned to the genome using GMAP (RRID:SCR_008992) [65] software, providing reliable gene structures for the genome. In addition, NGS transcriptome short reads were also used to align the genome using the TopHat package[66], and the gene structure was predicted using cufflinks[67]. All gene models were then integrated by MAKER (RRID:SCR_005309) [68] to obtain a consensus gene set.

For functional annotation of protein-coding genes in *P. canaliculata*, all gene sequences were searched against NCBI non-redundant protein (nr), non-redundant nucleotide (nt), and Swissprot databases using local BLASTX and BLASTN programs[63] with an e-value of $1e^{-5}$. Gene ontology (GO) and Kyoto Encyclopedia of Genes and Genomes (KEGG) pathway searches were then conducted on the resulting transcriptome using the software Blast2GO (RRID:SCR_005828) [69].

Detection of chromosomal rearrangement between reference genomes

To identity chromosomal rearrangements between genomes of Pcan_SH and previously published Pcan_SZ, the two genomes were first aligned using MUMmer4 (v4.00beta2)[70]. Alignment of the genomes was performed using

NUCmer (-c 1000), and then the alignment block filter was performed using a delta-filter with one-to-one alignment mode (-1 -i90 -l 10000). Finally, chromosomal rearrangements were called using the SyRI tool (RRID:SCR_023008) (v1.4) with default parameters[71], and Plotsr (v1.1.0)[72] were conducted to generates high-quality visualization of synteny and structural rearrangements.

Whole-genome population resequencing

We sampled 173 wild *P. canaliculata* individuals from 17 geographic distribution areas in EA and SEA for genome resequencing. Among the samples, 157 were collected from 12 provinces of China, with the remaining 16 samples being obtained from SEA countries (11 from Vietnam, 3 from Laos, and 2 from Cambodia) (supplementary table S1). Genomic DNA was extracted from the foot tissue using DNeasy Blood & Tissue Kits (QIAGEN). Two micrograms of gDNA from each individual was used to construct a sequencing library using a NEBNext Ultra DNA Library Prep Kit (NEB) following the manufacturer's instructions. Paired-end sequencing libraries with an insert size of approximately 350 bp were sequenced on an Illumina NovaSeq 6000 platform (RRID:SCR_016387) at Novogene-Beijing. All samples were sequenced to a target coverage of 10×. In addition, we downloaded the resequencing data for an individual from Argentina (Accession number: SRR8616636) and one *P. maculata* sample as an outgroup (Accession number: SRR8616630) reported in a previous study[33].

Variant calling, filtering, and annotation

We applied fastp (RRID:SCR_016962) [73] to filter the raw sequencing reads using the default parameters. The filtered reads were aligned to the new reference genome of *P. canaliculata* using BWA-MEM (RRID:SCR_022192) [74] with the -M parameter, and duplicates were marked using PicardTools MarkDuplicates (as part of GATK)[75]. Since whole genome SNP and INDEL databases of *P. canaliculata* were not available to perform the Base Quality Score Recalibrator (BQSR), we performed BQSR of non-human genomic data following GATK. We performed an initial round of joint-call cohort genotyping using the GATK HaplotypeCaller in gGVCF mode and GATK GenotypeGVCFs in succession. We then filtered variants with low quality using GATK VariantFiltration based on the following criteria: QD < 2.0, FS > 60.0, MQ < 40.0, SOR > 3.0, MQRankSum < −12.5, ReadPosRankSum < −8.0, QUAL < 30.0 for SNPs; and QD < 2.0, FS > 200.0, SOR > 10.0, MQRankSum < −12.5, ReadPosRankSum < −20.0, QUAL < 30.0 for INDELs. The variants passing the hard filtration were used as a true positive set of variant sites for BQSR with GATK BaseRecalibrator. We then repeated the joint-call cohort genotyping with the recalibrated BAM files and retained the variants if they met the above criteria.

Using VCFtools (RRID:SCR_001235) [76], we assigned the genotypes as missing if their quality scores (GQ) were less than 10 and excluded one sample with a high rate of missing SNPs (>30% of sites with a missing genotype). We

used the KING software[77] to calculate kinship coefficients between all pairwise combinations of samples. Forty samples that exhibiting greater than third-degree relationships with others were removed, leaving a total of 130 samples for subsequent analysis. Variants with none bi-allelic, > 5% missing calls, and MAF < 0.01 were removed to reduce false positives. The SNPable with 75-mer parameter and mDust procedures were used to mask regions of low mappability, and sites within these were also removed. This yielded a total of ~13.55 million variants for downstream analyses. Functional annotation of the retained variants was performed using the software ANNOVAR (RRID:SCR_012821) [78] with gene annotation for *P. canaliculata*.

Population genetic analysis

We pruned variants for LD in PLINK (RRID:SCR_001757) [79] with parameters --indep-pairwise 50 5 0.1 and --maf 0.05, which retained 266,653 SNPs for analysis of population structure. PCA was conducted at the individual level using the smartpca from the EIGENSOFT program[80] with the pruned SNP datasets. An ML phylogenetic tree was constructed by RAxML software (RRID:SCR_006086) [81] with the GTRGAMMA model and 1000 bootstrap replicates. *Pomacea maculata* was used as the outgroup. Software ADMIXTURE (RRID:SCR_001263) [82] was used to infer population genetic structure. Ten independent replications were performed for each of the ancestral numbers (K) from 2 to 10. The optimal K was determined according to the position with the minimum value of the five-fold cross-validation error.

VCFtools[76] was used to calculate the fixation index (F_{ST}), nucleotide diversity (π), and Tajima's *D* in 5-kb sliding, non-overlapping windows across each chromosome. Windows with fewer than 20 variants per 5-kb window were removed. Regression between the pairwise genetic distance ($F_{ST}/(1 - F_{ST})$) and geographic distance was calculated using a Mantel test as implemented in the Ecodist package for R. The significance of correlations was determined based on 1000 permutations. LD decay was estimated for each population using the PopLDdecay tool (RRID:SCR_022509) [83] that calculates the genotype correlation coefficient R^2 for pairs of SNPs at a maximum distance of 5 kb. The LD decay was measured as the chromosomal distance at which the average pairwise correlation decreased to half its maximum value.

Population splits and mixtures

TreeMix was applied to investigate the historical population relationships by estimating an ML population tree, the amount of genetic drift in each population, and the number of migration events (*m*) that best fitted the data[84]. *Pomacea maculata* was used as a root. Variants with missing rate > 1% or a minimum allele frequency < 0.05 in all samples were filtered out for further TreeMix analysis. In addition, we pruned any SNPs that were in LD using PLINK (--indep-pairwise 50 5 0.2) and retained 195,777 variants. We first ran TreeMix 20 times for each value of *m* ranging from 1 to 10 (-global -k 500 -se -bootstrap -noss). The optimal *m* value (*m*=2) was estimated using the OptM R package[85]. Then, a consensus ML tree including bootstrap node support was obtained by

running TreeMix 100 times for zero (as a null model) and seven migration events, followed by post-processing using the BITE R package.

The outgroup *f3* statistics were also estimated to infer the genetic affinities between the SEA populations and all other populations of *P. canaliculata*. To compute outgroup *f3* statistics of the form *f3* (X, Y; *P. maculata*) where *P. maculata* was selected as the target population, we applied the qp3pop module in the ADMIXTOOLS software[86].

Spatial variation in gene flow was investigated using Estimated Effective Migrations Surfaces (EEMS) analysis using 130 individuals, 5,000,000 MCMC (Markov Chain Monte Carlo) iterations, a burn-in of 1,000,000 iterations, and a thinning iteration of 9999 for each run. Parameters with 400 demes were carried out and plotted using rEEMSplots as the recommendation[87]. The habitat polygon was obtained using the Google Maps API v3 Tool and an individual genetic dissimilarity matrix was created using the bed2diffs function of EEMS.

Detecting genomic signatures for low-temperature adaptation

The BayPass program was used to identify SNPs with frequencies that were significantly associated with low temperature. For the ecotype divergence test, we retrieved the environmental variable Bio06 (Min Temperature of Coldest Month) for 17 geographic populations through the raster package in R and scaled the results so that the mean = 0 and variance = 1 as recommended[88]. Capitalizing on the large number of available SNPs, we sub-sampled by retaining one SNP every 100 SNPs along the genome, dividing the full SNP

dataset into 100 sub-datasets (each including ca. 135,544 SNPs). These sub-datasets were further analyzed in parallel using default options for the MCMC algorithm (except -npilot 15 -pilotlength 500 -burnin 2500). Three independent runs were performed for each dataset. We confirmed that the distance of covariance matrices (Ω) between replicates and between different sub-datasets was very low (fmd.dist < 1 as recommended in the BayPass manual), using the R function fmd.dist() included in BayPass. We also confirmed that all the obtained BF values across replicates had high correlations ($r > 0.7$). SNPs showing the median BF computed over the three runs greater than 20 dB were classified as outlier SNPs supported the significant association with low temperature.

Given that this study sought to characterize adaptation to climate, all individuals from Zhejiang, Shanghai, Jiangsu, Hubei, and Hunan provinces in EA, where the minimum temperature is below 2°C (Low Temperature sub-population, LT), and SEA (High Temperature sub-population, SEA) with high temperatures were selected for selection analysis. Two different haplotype-based methods (iHS, XP-EHH) and one allele frequency-based method (F_{ST}) were used to detect genomic signatures of positive selection. The pairwise population differentiation coefficient (F_{ST}) between the all LT populations and SEA populations was computed by VCFtools using a 10-kb sliding window with a step size of 5 kb[76]. We empirically selected the top 5% F_{ST} values as potential candidate regions under selection. After phasing the SNP dataset using SHAPEIT2[89], we

calculated the integrated haplotype scores (iHS) and cross-population extended haplotype homozygosity (XP-EHH) using Selscan[90] for each chromosome separately. The XP-EHH score was positive, reflecting the presence of extended haplotypes in the LT population. Using the norm module implemented in Selscan, the *P. canaliculata* genome was divided into non-overlapping 10-kb regions, and both the fraction of XP-EHH scores > 2 and that of |iHS| > 2 were computed. The top 5% of windows with the highest fraction of extreme scores were considered as candidate selective regions. To reduce the false-positive regions in the detection, potential candidate regions defined by at least two of the above-mentioned methods were considered as the final candidate regions for selection.

Detecting genomic signatures of balancing selection

Genomic scans for balancing selection (BS) were performed for the EA and SEA populations using the standardized β and NCD statistics. To reduce false positives, genotypes were marked as missing when the proportion of reads that uniquely mapped was below 80%. Further, we retained the genotype only when the depth of variants is at least one-third of the sample average read depth and no more than two fold of the read depth. Only SNPs with a MAF > 0.05 and missing call rate < 5% in each population were retained for balance selection. High β scores indicated an excess of SNPs at similar frequencies, while low NCD scores indicated a build-up of SNPs near a specified intermediate frequency, both of which are potential consequences of long-term BS. For

standardized β scores, we applied the toolkit glactools[91] for file format conversion and ran BetaScan software[92] to calculate the β score to detect BS with the parameter "-fold -m 0.15" that refers to the minimum fold frequency of core SNPs. The conserved BS sites were identified as those SNPs with standardized β scores in the top 99.5th percentile in each population, and the sliding 10-kb windows with two or more such outlier SNPs were defined as the BS genomic regions. The NCD statistics measure the average difference between allele frequencies in a given region from a deviation point, while BetaScan measures β scores for individual SNPs. To facilitate comparison between the two statistics, a custom Python script was used to calculate a modified NCD statistic for each SNP in both SEA and EA populations with windows of 500 bp around every SNP and considering a target frequency of 0.5[93]. Three additional statistics, namely Tajima's *D*, nucleotide diversity (π), and observed heterozygosity (*Ho*), were applied to confirm the top signals.

Functional enrichment analyses

Approximate gene annotations were obtained by assigning the candidate selective regions to their closest gene model in the *P. canaliculata* genome using BEDOPS[94]. GO enrichment tests were performed to detect functional groups using the clusterProfiler package[95] in R. An unadjusted *P*-value < 0.01 was assumed as the threshold for significant enrichment.

Differential gene expression

We identified gene expression profile of *P. canaliculata* in seven tissues

including embryos, gill, hemocytes, hepatopancreas, kidney, ovary and albumen gland, and testis with the public RNA-Seq data[33] (BioProject PRJNA473031). Besides, RNA-Seq data of *P. canaliculata* under different abiotic stress conditions were also analyzed, including heat, cold, heavy metal tolerance and air exposure (BioProject PRJNA427478). Reads were downloaded from the SRA database[16] and trimmed off adapters and low-quantity bases with TrimGalore. Trimmed reads were then mapped to the *P. canaliculata* genome using HISAT2 (RRID:SCR_015530)[96], and the gene raw read count was obtained with featureCounts in Subread (RRID:SCR_009803) [97]. DESeq2[98] in R was used to identify DEGs. A gene with a fold change > 1.2 (upregulated) or < 0.83 (downregulated) and FDR adjusted *P*-value < 0.05 was considered to be a DEG. The genes identified among the selection results were selected for plotting using R.

RT-qPCR validation of the *csde1* gene expression under cold tolerance

Snails with similar size were reared in freshwater at 25°C for at least 10 days for acclimation and then randomly divided into two groups with three replicates of five snails each. The control group was exposed to normal temperature (25 °C), while the experimental group was exposed to 0 °C for 5 d in an incubator. The hemocytes were then collected at 0 h, 12 h, 1 d, 3 d, 5 d after exposure. Total RNA was extracted with TRIzol@ reagent (Takara Bio Inc.) and assessed using a Nanodrop 2000 spectrophotometer (Nanodrop

Technologies Inc.). Reverse transcription quantitative PCR (RT-qPCR) was performed to further investigate the expression of the *csde1* gene in each sample in duplicate using SYBR qPCR Master Mix (Vazyme) in a 20-μL reaction volume. Primers for qPCR were designed with Primer Premier v5 with *β-actin* as the internal control (supplementary table S12). The relative expression levels of the *csde1* genes were calculated by the comparative cycle threshold (Ct) method ($2^{-\Delta\Delta Ct}$) and subjected to statistical analysis with Prism v9.

Acknowledgments

This research was supported by the National Key Research and Development Program of China (No. 2016YFC1200503, No. 2021YFC2300800, 2021YFC2300802, 2021YFC2300803).

Author Contributions

H.W. and L.Y. designed, supervised the research. L.Y. and L.F. wrote the original manuscript. L.Y. and X.S. revised the manuscript. L.Y., L.F., Z.A. and C.H. performed the data analysis and curation. G.Y.H, Z.Y., L.J., W.Z.D., Z.W., L.D.T., and Y.Y.M. collected the samples. L.Y., L.F., L.J. and X.Y.X. prepared the materials for sequencing. Y.C. performed the laboratory experiments. All of the authors critically reviewed and proved the final manuscript.

Declaration of interests

The authors declare that there is no competing interest existing.

Data availability

The genome sequence data for *P. canaliculata* are deposited in NCBI under

SRA accession number PRJNA951867. The assembly and annotation files are available under the NCBI accession PRJNA951865. The whole genome re-sequencing data for *P. canaliculata* can be accessed with the accession number PRJNA951872 in NCBI. All additional supporting data are available in the *GigaScience* repository, GigaDB [99].

References

1. Cowie RH. Apple snails (Ampullariidae) as agricultural pests: Their biology, impacts and management. CABI Publishing, Wallingford; 2002.
2. S L, M B, S B and M DP. 100 of the World's Worst Invasive Alien Species A selection from the Global Invasive Species Database. The Invasive Species Specialist Group (ISSG) a specialist group of the Species Survival Commission (SSC) of the World Conservation Union (IUCN). 2000.
3. Yang QQ, Liu SW, He C and Yu XP. Distribution and the origin of invasive apple snails, Pomacea canaliculata and P. maculata (Gastropoda: Ampullariidae) in China. Sci Rep. 2018;8 1:1185. doi:10.1038/s41598-017-19000-7.
4. Yang TB, Wu ZD and Lun ZR. The apple snail Pomacea canaliculata, a novel vector of the rat lungworm, Angiostrongylus cantonensis: its introduction, spread, and control in China. Hawaii J Med Public Health. 2013;72 6 Suppl 2:23-5.
5. Tesana S, Srisawangwong T, Sithithaworn P and Laha T. Angiostrongylus cantonensis: experimental study on the susceptibility of apple snails, Pomacea canaliculata compared to Pila polita. Exp Parasitol. 2008;118 4:531-5. doi:10.1016/j.exppara.2007.11.007.
6. Kim JR, Hayes KA, Yeung NW and Cowie RH. Correction: Diverse Gastropod Hosts of Angiostrongylus cantonensis, the Rat Lungworm, Globally and with a Focus on the Hawaiian Islands. PLoS One. 2018;13 2:e0193556. doi:10.1371/journal.pone.0193556.
7. Kim JR, Hayes KA, Yeung NW and Cowie RH. Diverse gastropod hosts of Angiostrongylus cantonensis, the rat lungworm, globally and with a focus on the Hawaiian Islands. PLoS One. 2014;9 5:e94969. doi:10.1371/journal.pone.0094969.
8. Hayes KA, Joshi RC, Thiengo SC and Cowie RH. Out of South America: Multiple Origins of Non-Native Apple Snails in Asia. Diversity and Distributions. 2008;14 4:701-12.
9. Byers JE, McDowell WG, Dodd SR, Haynie RS, Pintor LM and Wilde SB. Climate and pH predict the potential range of the invasive apple snail (Pomacea insularum) in the southeastern United States. PLoS One. 2013;8 2:e56812. doi:10.1371/journal.pone.0056812.
10. Gilioli G, Pasquali S, Martin PR, Carlsson N and Mariani L. A temperature-dependent physiologically based model for the invasive apple snail Pomacea canaliculata. Int J Biometeorol. 2017;61 11:1899-911. doi:10.1007/s00484-017-1376-3.
11. Yoshida K, Matsukura K, Cazzaniga NJ and Wada T. Tolerance to low temperature and

desiccation in two invasive apple snails, Pomacea Canaliculata and P. Maculata (Caenogastropoda: Ampullariidae), collected in their original distribution area (northern and central Argentina). Journal of Molluscan Studies. 2014;80:62–6.
12. Qin Z, Wu RS, Zhang J, Deng ZX, Zhang CX and Guo J. Survivorship of geographic Pomacea canaliculata populations in responses to cold acclimation. Ecol Evol. 2020;10 8:3715-26. doi:10.1002/ece3.6162.
13. Song HM, Mu XD, Gu DE, Luo D, Yang YX, Xu M, et al. Molecular characteristics of the HSP70 gene and its differential expression in female and male golden apple snails (Pomacea canaliculata) under temperature stimulation. Cell Stress Chaperones. 2014;19 4:579-89. doi:10.1007/s12192-013-0485-0.
14. Liu GF, Yang QQ, Lin HF and Xu XP. Differential gene expression in Pomacea canaliculata (Mollusca: Gastropoda) under low temperature condition. Journal of Molluscan Studies. 2018;84 4:397–403.
15. Xiao Q, Lin Y, Li H, Chen Y, Wei W, Li P, et al. Transcriptome sequencing reveals the differentially expressed lncRNAs and mRNAs in response to cold acclimation and cold stress in Pomacea canaliculata. BMC Genomics. 2022;23 1:382. doi:10.1186/s12864-022-08622-5.
16. Liu C, Zhang Y, Ren Y, Wang H, Li S, Jiang F, et al. The genome of the golden apple snail *Pomacea canaliculata* provides insight into stress tolerance and invasive adaptation. GigaScience. 2018;7 9:giy101. doi:10.1093/gigascience/giy101.
17. Hughes AR and Stachowicz JJ. Genetic diversity enhances the resistance of a seagrass ecosystem to disturbance. Proc Natl Acad Sci U S A. 2004;101 24:8998-9002. doi:10.1073/pnas.0402642101.
18. Ito K. Environmental factors influencing overwintering success of the golden apple snail, Pomacea canaliculata (Gastropoda: Ampullariidae), in the northernmost population of Japan. App Ent Zool. 2002;37:655–61.
19. Matsukura K, Tsumuki H, Izumi Y and Wada T. Changes in chemical components in the freshwater apple snail, Pomacea canaliculata (Gastropoda: Ampullariidae), in relation to the development of its cold hardiness. Cryobiology. 2008;56 2:131-7. doi:10.1016/j.cryobiol.2007.12.001.
20. Matsukura K, Tsumuki H, Izumi Y and Wada T. Physiological response to low temperature in the freshwater apple snail, Pomacea canaliculata (Gastropoda: Ampullariidae). J Exp Biol. 2009;212 Pt 16:2558-63. doi:10.1242/jeb.031500.
21. Zhang Z, Machado F, Zhao L, Heinen CA, Foppen E, Ackermans MT, et al. Administration of Thyrotropin-Releasing Hormone in the Hypothalamic Paraventricular Nucleus of Male Rats Mimics the Metabolic Cold Defense Response. Neuroendocrinology. 2018;107 3:267-79. doi:10.1159/000492785.
22. Nillni EA, Xie W, Mulcahy L, Sanchez VC and Wetsel WC. Deficiencies in pro-thyrotropin-releasing hormone processing and abnormalities in thermoregulation in Cpefat/fat mice. J Biol Chem. 2002;277 50:48587-95. doi:10.1074/jbc.M206702200.
23. Yamada M, Saga Y, Shibusawa N, Hirato J, Murakami M, Iwasaki T, et al. Tertiary hypothyroidism and hyperglycemia in mice with targeted disruption of the thyrotropin-releasing hormone gene. Proc Natl Acad Sci U S A. 1997;94 20:10862-7. doi:10.1073/pnas.94.20.10862.
24. Yamanaka K, Fang L and Inouye M. The CspA family in Escherichia coli: multiple gene duplication for stress adaptation. Mol Microbiol. 1998;27 2:247-55. doi:10.1046/j.1365-

2958.1998.00683.x.

25. Karlson D and Imai R. Conservation of the cold shock domain protein family in plants. Plant Physiol. 2003;131 1:12-5. doi:10.1104/pp.014472.

26. Wilkie GS, Dickson KS and Gray NK. Regulation of mRNA translation by 5'- and 3'-UTR-binding factors. Trends Biochem Sci. 2003;28 4:182-8. doi:10.1016/S0968-0004(03)00051-3.

27. Forester BR, Lasky JR, Wagner HH and Urban DL. Comparing methods for detecting multilocus adaptation with multivariate genotype-environment associations. Mol Ecol. 2018;27 9:2215-33. doi:10.1111/mec.14584.

28. Jensen BS and Fago A. Sulfide metabolism and the mechanism of torpor. J Exp Biol. 2021;224 17 doi:10.1242/jeb.215764.

29. Youn JY, Dunham WH, Hong SJ, Knight JDR, Bashkurov M, Chen GI, et al. High-Density Proximity Mapping Reveals the Subcellular Organization of mRNA-Associated Granules and Bodies. Mol Cell. 2018;69 3:517-32 e11. doi:10.1016/j.molcel.2017.12.020.

30. Zhang W and Liu HT. MAPK signal pathways in the regulation of cell proliferation in mammalian cells. Cell Res. 2002;12 1:9-18. doi:10.1038/sj.cr.7290105.

31. Spangler JB, Moraga I, Mendoza JL and Garcia KC. Insights into cytokine-receptor interactions from cytokine engineering. Annu Rev Immunol. 2015;33:139-67. doi:10.1146/annurev-immunol-032713-120211.

32. Zhou H, Huang Y, Jia C, Pang Y, Liu L, Xu Y, et al. NF-kappaB factors cooperate with Su(Hw)/E4F1 to balance Drosophila/human immune responses via modulating dynamic expression of miR-210. Nucleic Acids Res. 2024; doi:10.1093/nar/gkae394.

33. Sun J, Mu H, Ip JCH, Li R, Xu T, Accorsi A, et al. Signatures of Divergence, Invasiveness, and Terrestrialization Revealed by Four Apple Snail Genomes. Molecular Biology and Evolution. 2019;36 7:1507-20. doi:10.1093/molbev/msz084.

34. Al-Khalaifah H. Cellular and humoral immune response between snail hosts and their parasites. Front Immunol. 2022;13:981314. doi:10.3389/fimmu.2022.981314.

35. Liu K, Xiao C, Xi S, Hameed M, Wahaab A, Shao D, et al. Mosquito Defensins Enhance Japanese Encephalitis Virus Infection by Facilitating Virus Adsorption and Entry within the Mosquito. J Virol. 2020;94 21 doi:10.1128/JVI.01164-20.

36. Sun Y, Zhu X, Chen X, Liu H, Xu Y, Chu Y, et al. The mediator subunit Med23 contributes to controlling T-cell activation and prevents autoimmunity. Nat Commun. 2014;5:5225. doi:10.1038/ncomms6225.

37. Jiang FJ, Yue X, Zhang SJ, Yu JJ, Wang R, Liu BZ, et al. Heritability of resistance-related gene expression traits and their correlation with body size of clam Meretrix petechialis. Journal of Oceanology and Limnology 2019;38:571–8.

38. Dolan J, Walshe K, Alsbury S, Hokamp K, O'Keeffe S, Okafuji T, et al. The extracellular leucine-rich repeat superfamily; a comparative survey and analysis of evolutionary relationships and expression patterns. BMC Genomics. 2007;8:320. doi:10.1186/1471-2164-8-320.

39. Saco A, Rey-Campos M, Novoa B and Figueras A. Transcriptomic Response of Mussel Gills After a Vibrio splendidus Infection Demonstrates Their Role in the Immune Response. Front Immunol. 2020;11:615580. doi:10.3389/fimmu.2020.615580.

40. Guo Z, Kang S, Wu Q, Wang S, Crickmore N, Zhou X, et al. The regulation landscape of MAPK signaling cascade for thwarting Bacillus thuringiensis infection in an insect host. PLoS Pathog. 2021;17 9:e1009917. doi:10.1371/journal.ppat.1009917.

41. Tscherner M, Zwolanek F, Jenull S, Sedlazeck FJ, Petryshyn A, Frohner IE, et al. The Candida albicans Histone Acetyltransferase Hat1 Regulates Stress Resistance and Virulence via Distinct Chromatin Assembly Pathways. PLoS Pathog. 2015;11 10:e1005218. doi:10.1371/journal.ppat.1005218.
42. Gao Y, Li JN, Pu JJ, Tao KX, Zhao XX and Yang QQ. Genome-wide identification and characterization of the HSP gene superfamily in apple snails (Gastropoda: Ampullariidae) and expression analysis under temperature stress. Int J Biol Macromol. 2022;222 Pt B:2545-55. doi:10.1016/j.ijbiomac.2022.10.038.
43. Giraud-Billoud M, Vega IA, Tosi ME, Abud MA, Calderon ML and Castro-Vazquez A. Antioxidant and molecular chaperone defences during estivation and arousal in the South American apple snail Pomacea canaliculata. J Exp Biol. 2013;216 Pt 4:614-22. doi:10.1242/jeb.075655.
44. Xu Y, Zheng G, Dong S, Liu G and Yu X. Molecular cloning, characterization and expression analysis of HSP60, HSP70 and HSP90 in the golden apple snail, Pomacea canaliculata. Fish Shellfish Immunol. 2014;41 2:643-53. doi:10.1016/j.fsi.2014.10.013.
45. Riemschoss K, Arndt V, Bolognesi B, von Eisenhart-Rothe P, Liu S, Buravlova O, et al. Fibril-induced glutamine-/asparagine-rich prions recruit stress granule proteins in mammalian cells. Life Sci Alliance. 2019;2 4 doi:10.26508/lsa.201800280.
46. Liu F, Xiao Y, Ji XL, Zhang KQ and Zou CG. The cAMP-PKA pathway-mediated fat mobilization is required for cold tolerance in C. elegans. Sci Rep. 2017;7 1:638. doi:10.1038/s41598-017-00630-w.
47. Aguilera J, Randez-Gil F and Prieto JA. Cold response in Saccharomyces cerevisiae: new functions for old mechanisms. FEMS Microbiol Rev. 2007;31 3:327-41. doi:10.1111/j.1574-6976.2007.00066.x.
48. Bakakina YS, Kolesneva EV, Sodel DL, Dubovskaya LV and ID V. Low and High Temperatures Enhance Guanylyl Cyclase Activity in Arabidopsis Seedlings. J Plant Physiol Pathol 2014;2 4 doi:doi:10.4172/2329-955X.1000132.
49. Ootsuka Y, Heidbreder CA, Hagan JJ and Blessing WW. Dopamine D2 receptor stimulation inhibits cold-initiated thermogenesis in brown adipose tissue in conscious rats. Neuroscience. 2007;147 1:127-35. doi:10.1016/j.neuroscience.2007.04.015.
50. Minias P and Vinkler M. Selection Balancing at Innate Immune Genes: Adaptive Polymorphism Maintenance in Toll-Like Receptors. Mol Biol Evol. 2022;39 5 doi:10.1093/molbev/msac102.
51. Dudchenko O, Batra SS, Omer AD, Nyquist SK, Hoeger M, Durand NC, et al. *De novo* assembly of the *Aedes aegypti* genome using Hi-C yields chromosome-length scaffolds. Science. 2017;356 6333:92-5. doi:10.1126/science.aal3327.
52. Chin CS, Peluso P, Sedlazeck FJ, Nattestad M, Concepcion GT, Clum A, et al. Phased diploid genome assembly with single-molecule real-time sequencing. Nat Methods. 2016;13 12:1050-4. doi:10.1038/nmeth.4035.
53. Koren S, Walenz BP, Berlin K, Miller JR, Bergman NH and Phillippy AM. Canu: scalable and accurate long-read assembly via adaptive k-mer weighting and repeat separation. Genome Research. 2017;27 5:722-36. doi:10.1101/gr.215087.116.
54. Vaser R, Sovic I, Nagarajan N and Sikic M. Fast and accurate *de novo* genome assembly from long uncorrected reads. Genome Research. 2017;27 5:gr.214270.116.
55. Chin C-S, Alexander DH, Marks P, Klammer AA, Drake J, Heiner C, et al. Nonhybrid, finished

microbial genome assemblies from long-read SMRT sequencing data. Nature Methods. 2013;10 6:563-9. doi:10.1038/nmeth.2474.

56. Walker BJ, Abeel T, Shea T, Priest M, Abouelliel A, Sakthikumar S, et al. Pilon: An Integrated Tool for Comprehensive Microbial Variant Detection and Genome Assembly Improvement. PLOS ONE. 2014;9 11:e112963. doi:10.1371/journal.pone.0112963.

57. Burton JN, Adey A, Patwardhan RP, Qiu R, Kitzman JO and Shendure J. Chromosome-scale scaffolding of *de novo* genome assemblies based on chromatin interactions. Nature Biotechnology. 2013;31 12:1119-25. doi:10.1038/nbt.2727.

58. Parra G, Bradnam K and Korf I. CEGMA: a pipeline to accurately annotate core genes in eukaryotic genomes. Bioinformatics. 2007;23 9:1061.

59. Simão FA, Waterhouse RM, Ioannidis P, Kriventseva EV and Zdobnov EM. BUSCO: assessing genome assembly and annotation completeness with single-copy orthologs. Bioinformatics. 2015;31 19:3210.

60. Tarailo-Graovac M and Chen N. Using RepeatMasker to Identify Repetitive Elements in Genomic Sequences. Current Protocols in Bioinformatics. 2009;25 1:4.10.1-4..4. doi:https://doi.org/10.1002/0471250953.bi0410s25.

61. Stanke M, Keller O, Gunduz I, Hayes A, Waack S and Morgenstern B. AUGUSTUS: *ab initio* prediction of alternative transcripts. Nucleic Acids Research. 2006;34 Web Server issue:435-9.

62. Flicek P, Amode MR, Barrell D, Beal K, Billis K, Brent S, et al. Ensembl 2014. Nucleic Acids Research. 2014;42 Database issue:D749-D55.

63. Lobo I. Basic Local Alignment Search Tool (BLAST). Journal of Molecular Biology. 2008;215 3:403-10.

64. Birney E, Clamp M and Durbin R. GeneWise and Genomewise. Genome Research. 2004;14 5:988.

65. Wu TD and Watanabe CK. GMAP: a genomic mapping and alignment program for mRNA and EST sequences. Bioinformatics. 2005;21 9:1859.

66. Trapnell C, Pachter L and Salzberg SL. TopHat: discovering splice junctions with RNA-Seq. Bioinformatics. 2009;25 9:1105-11.

67. Ghosh S and Chan CKK. Analysis of RNA-Seq Data Using TopHat and Cufflinks. Methods in Molecular Biology. 2016;1374:339.

68. Campbell MS, Holt C, Moore B and Yandell M. Genome Annotation and Curation Using MAKER and MAKER-P. Current Protocols in Bioinformatics. 2014;48:4.11.1.

69. Conesa A, Götz S, Garcíagómez JM, Terol J, Talón M and Robles M. Blast2GO: a universal tool for annotation, visualization and analysis in functional genomics research. Bioinformatics. 2005;21 18:3674.

70. Marçais G, Delcher AL, Phillippy AM, Coston R, Salzberg SL and Zimin A. MUMmer4: A fast and versatile genome alignment system. PLOS Computational Biology. 2018;14 1:e1005944. doi:10.1371/journal.pcbi.1005944.

71. Goel M, Sun H, Jiao W-B and Schneeberger K. SyRI: finding genomic rearrangements and local sequence differences from whole-genome assemblies. Genome Biology. 2019;20 1:277. doi:10.1186/s13059-019-1911-0.

72. Goel M and Schneeberger K. plotsr: visualizing structural similarities and rearrangements between multiple genomes. Bioinformatics. 2022;38 10:2922-6. doi:10.1093/bioinformatics/btac196.

73. Chen S, Zhou Y, Chen Y and Gu J. fastp: an ultra-fast all-in-one FASTQ preprocessor. Bioinformatics. 2018;34 17:i884-i90. doi:10.1093/bioinformatics/bty560.

74. Li H and Durbin R. Fast and accurate long-read alignment with Burrows-Wheeler transform. Bioinformatics. 2010;26 5:589-95. doi:10.1093/bioinformatics/btp698.

75. McKenna A, Hanna M, Banks E, Sivachenko A, Cibulskis K, Kernytsky A, et al. The Genome Analysis Toolkit: a MapReduce framework for analyzing next-generation DNA sequencing data. Genome Res. 2010;20 9:1297-303. doi:10.1101/gr.107524.110.

76. Danecek P, Auton A, Abecasis G, Albers CA, Banks E, DePristo MA, et al. The variant call format and VCFtools. Bioinformatics. 2011;27 15:2156-8. doi:10.1093/bioinformatics/btr330.

77. Manichaikul A, Mychaleckyj JC, Rich SS, Daly K, Sale M and Chen W-M. Robust relationship inference in genome-wide association studies. Bioinformatics. 2010;26 22:2867-73. doi:10.1093/bioinformatics/btq559.

78. Wang K, Li M and Hakonarson H. ANNOVAR: functional annotation of genetic variants from high-throughput sequencing data. Nucleic acids research. 2010;38 16:e164-e. doi:10.1093/nar/gkq603.

79. Purcell S, Neale B, Todd-Brown K, Thomas L, Ferreira MA, Bender D, et al. PLINK: a tool set for whole-genome association and population-based linkage analyses. Am J Hum Genet. 2007;81 3:559-75. doi:10.1086/519795.

80. Patterson N, Price AL and Reich D. Population Structure and Eigenanalysis. PLOS Genetics. 2006;2 12:e190. doi:10.1371/journal.pgen.0020190.

81. Stamatakis A. RAxML version 8: a tool for phylogenetic analysis and post-analysis of large phylogenies. Bioinformatics. 2014;30 9:1312-3. doi:10.1093/bioinformatics/btu033.

82. Alexander DH, Novembre J and Lange K. Fast model-based estimation of ancestry in unrelated individuals. Genome Research. 2009;19 9:1655-64. doi:10.1101/gr.094052.109.

83. Zhang C, Dong S-S, Xu J-Y, He W-M and Yang T-L. PopLDdecay: a fast and effective tool for linkage disequilibrium decay analysis based on variant call format files. Bioinformatics. 2019;35 10:1786-8. doi:10.1093/bioinformatics/bty875.

84. Pickrell JK and Pritchard JK. Inference of Population Splits and Mixtures from Genome-Wide Allele Frequency Data. PLOS Genetics. 2012;8 11:e1002967. doi:10.1371/journal.pgen.1002967.

85. Fitak RR. OptM: estimating the optimal number of migration edges on population trees using Treemix. Biol Methods Protoc. 2021;6 1:bpab017. doi:10.1093/biomethods/bpab017.

86. Patterson N, Moorjani P, Luo Y, Mallick S, Rohland N, Zhan Y, et al. Ancient Admixture in Human History. Genetics. 2012;192 3:1065-93. doi:10.1534/genetics.112.145037.

87. Petkova D, Novembre J and Stephens M. Visualizing spatial population structure with estimated effective migration surfaces. Nature Genetics. 2016;48 1:94-100. doi:10.1038/ng.3464.

88. Gautier M. Genome-Wide Scan for Adaptive Divergence and Association with Population-Specific Covariates. Genetics. 2015;201 4:1555-79. doi:10.1534/genetics.115.181453.

89. Delaneau O, Zagury J-F, Robinson MR, Marchini JL and Dermitzakis ET. Accurate, scalable and integrative haplotype estimation. Nature Communications. 2019;10 1:5436. doi:10.1038/s41467-019-13225-y.

90. Szpiech ZA. selscan 2.0: scanning for sweeps in unphased data. bioRxiv. 2021:2021.10.22.465497. doi:10.1101/2021.10.22.465497. Bioinformatics. 2024. 2;40(1):btae006. doi: 10.1093/bioinformatics/btae006.

91. Renaud G. glactools: a command-line toolset for the management of genotype likelihoods and allele counts. Bioinformatics. 2018;34 8:1398-400. doi:10.1093/bioinformatics/btx749.
92. Siewert KM and Voight BF. BetaScan2: Standardized Statistics to Detect Balancing Selection Utilizing Substitution Data. Genome Biology and Evolution. 2020;12 2:3873-7. doi:10.1093/gbe/evaa013.
93. Stern DB and Lee CE. Evolutionary origins of genomic adaptations in an invasive copepod. Nature Ecology & Evolution. 2020;4 8:1084-94. doi:10.1038/s41559-020-1201-y.
94. Neph S, Kuehn MS, Reynolds AP, Haugen E, Thurman RE, Johnson AK, et al. BEDOPS: high-performance genomic feature operations. Bioinformatics. 2012;28 14:1919-20. doi:10.1093/bioinformatics/bts277.
95. Yu G, Wang LG, Han Y and He QY. clusterProfiler: an R package for comparing biological themes among gene clusters. Omics. 2012;16 5:284-7. doi:10.1089/omi.2011.0118.
96. Kim D, Langmead B and Salzberg SL. HISAT: a fast spliced aligner with low memory requirements. Nature Methods. 2015;12 4:357-60. doi:10.1038/nmeth.3317.
97. Liao Y, Smyth GK and Shi W. featureCounts: an efficient general purpose program for assigning sequence reads to genomic features. Bioinformatics. 2014;30 7:923-30. doi:10.1093/bioinformatics/btt656.
98. Love MI, Huber W and Anders S. Moderated estimation of fold change and dispersion for RNA-seq data with DESeq2. Genome Biology. 2014;15 12:550. doi:10.1186/s13059-014-0550-8.
99. Lu Y, Luo F, Zhou A, Yi C, Chen H, Li J, et al. Supporting data for "Whole-genome sequencing of the invasive golden apple snail Pomacea canaliculata from Asia reveals rapid expansion and adaptive evolution" GigaScience Database. 2024. https://doi.org/10.5524/102564

Figure 1

Click here to access/download;Figure;figure 1.tif

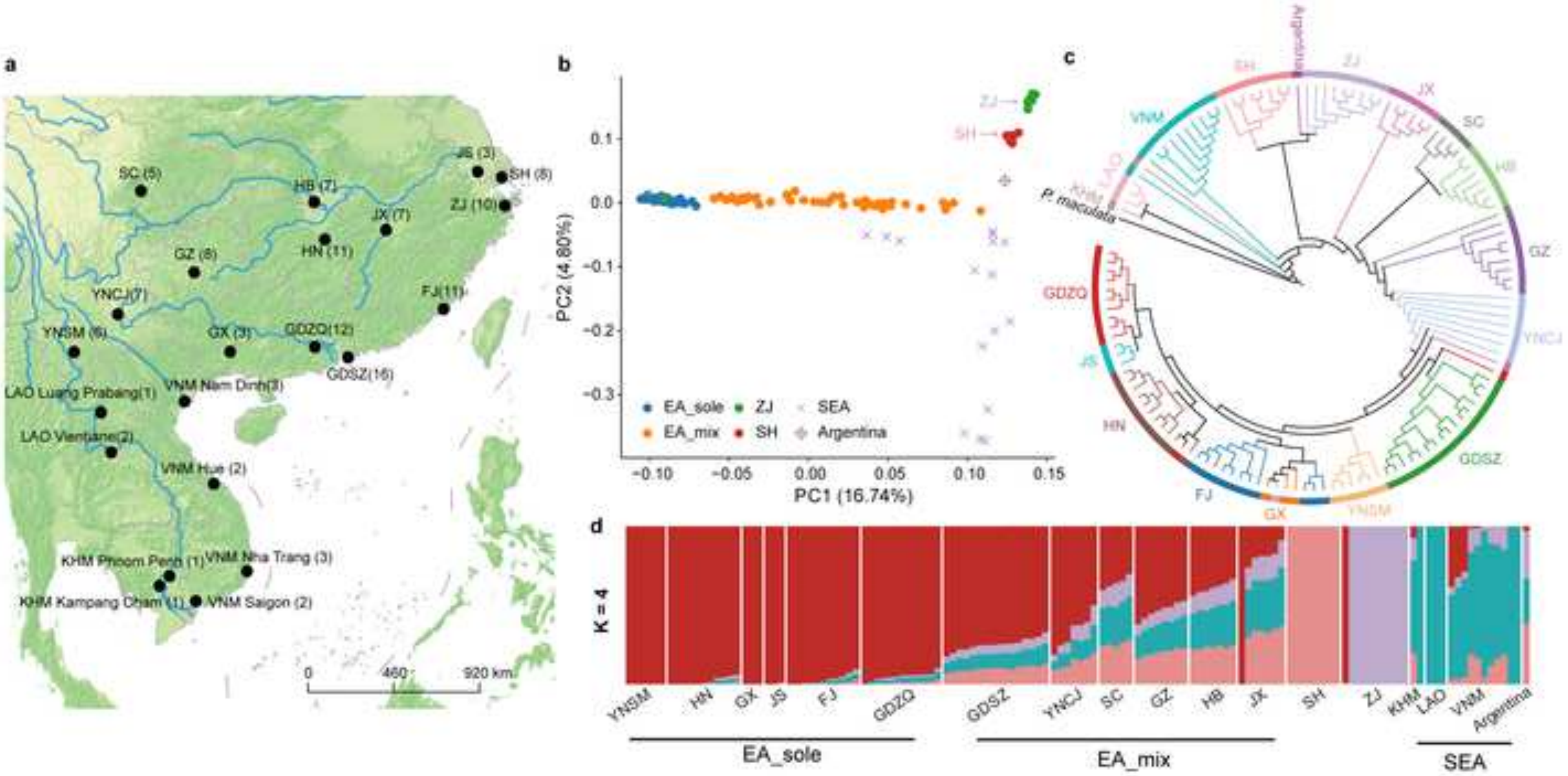

Figure 2

Click here to access/download;Figure;figure 2.tif

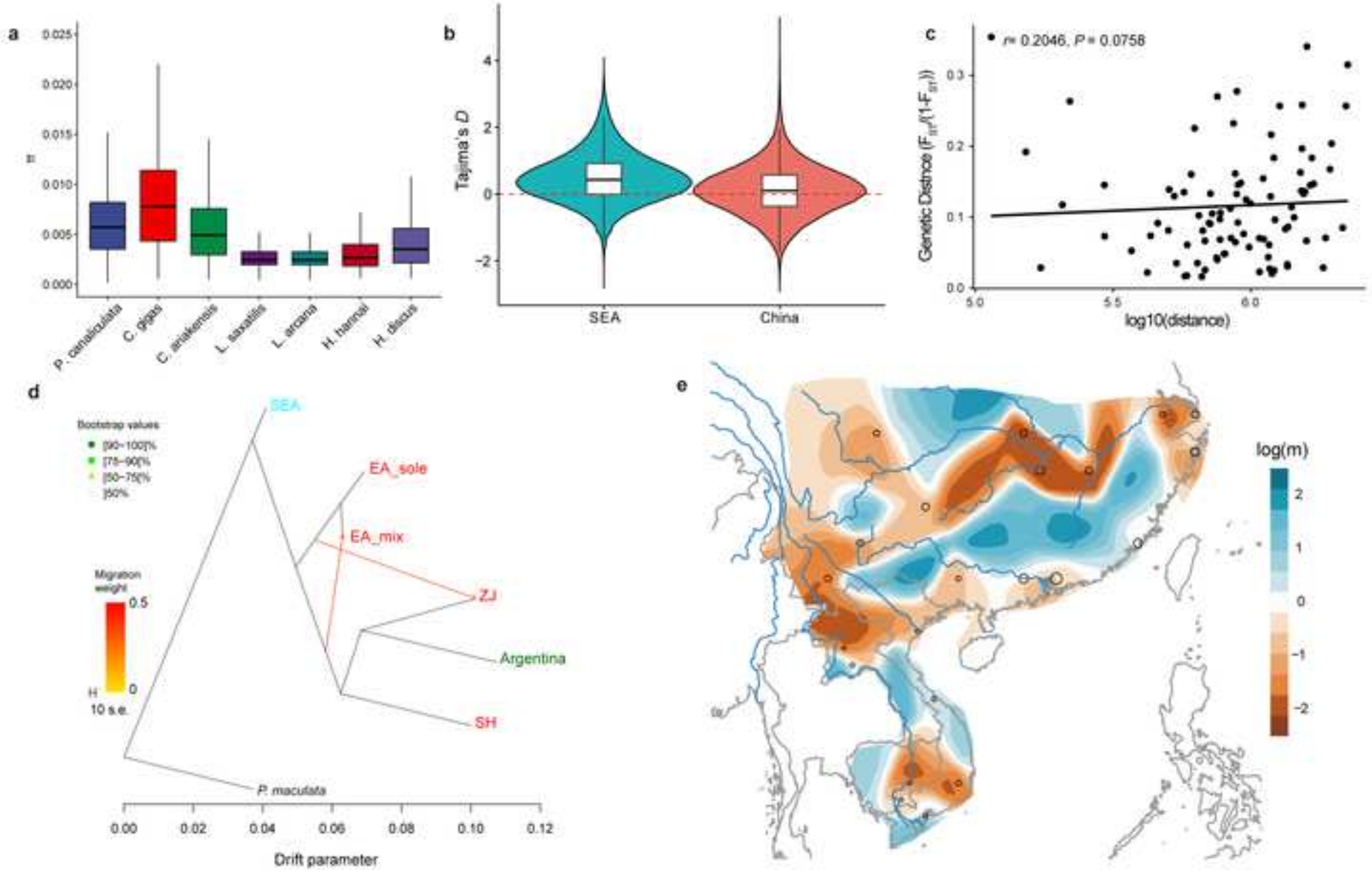

Figure 3

Click here to access/download;Figure;figure 3.tif

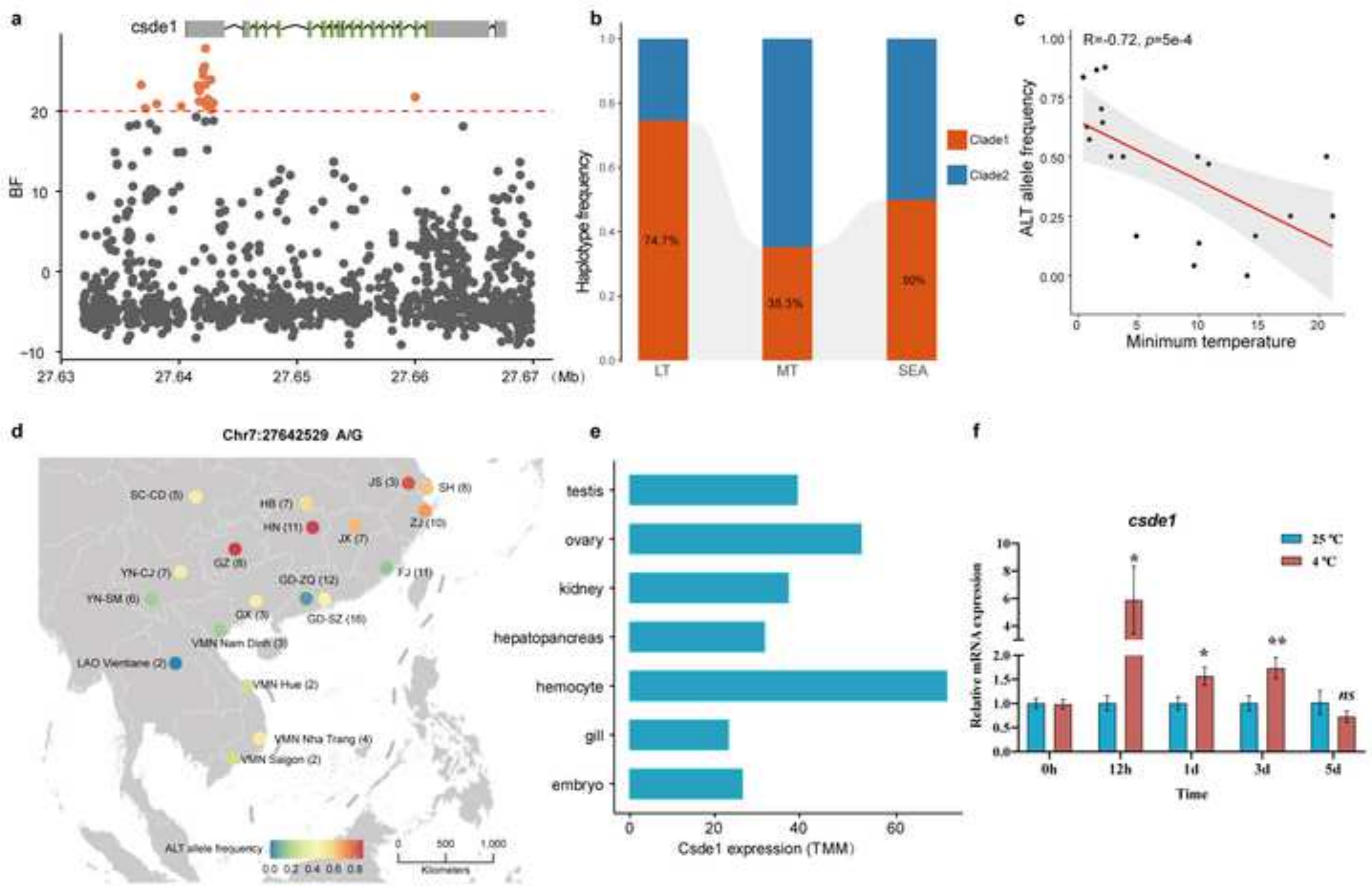

Figure 4

Click here to access/download;Figure;figure 4.tif

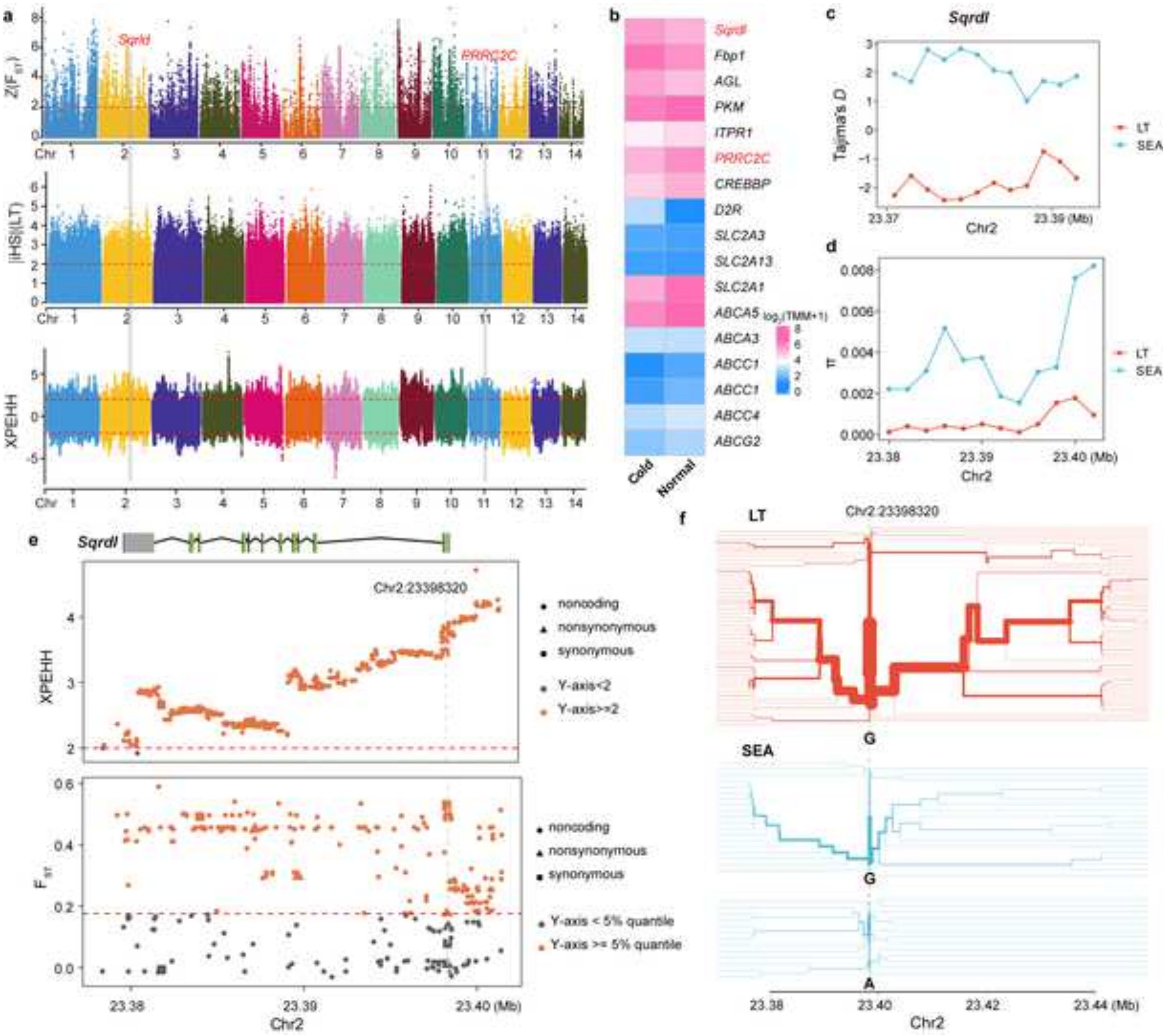

Click here to access/download;Figure;Figure5.tif

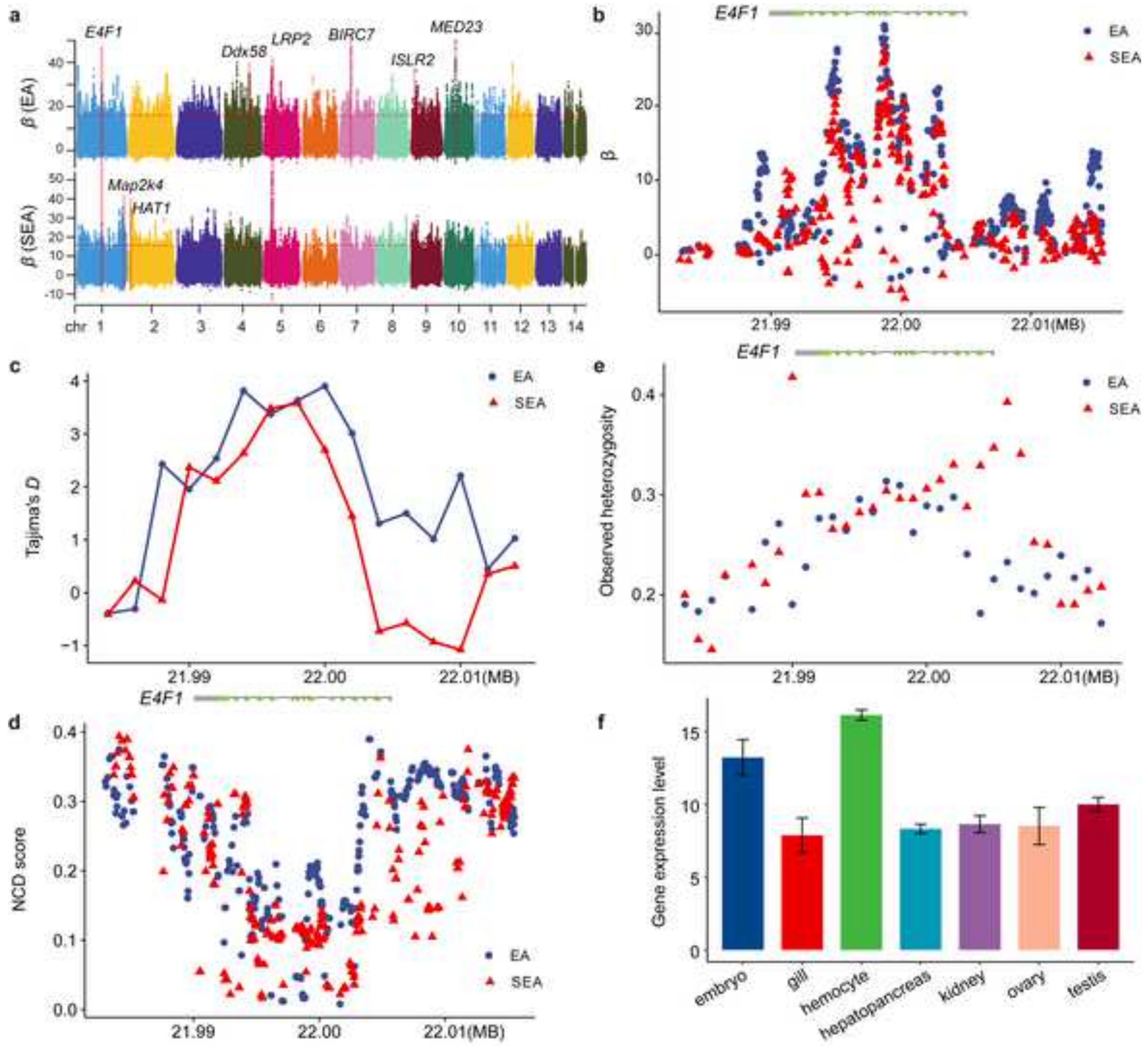

Click here to access/download
Supplementary Material
Supplementary_240618.docx

Supplementary tables

Click here to access/download
Supplementary Material
SI_Table_240618.xlsx
