# Supplementary material for: Whole-genome sequencing of the invasive golden apple snail Pomacea canaliculata from Asia reveals rapid expansion and adaptive evolution
Source: Gigascience. 2024 Sep 23;13:giae064. doi: 10.1093/gigascience/giae064 (PMC11417965; doi:10.1093/gigascience/giae064)
Supplement: giae064_Supplemental_Files [file giae064_supplemental_files.zip › Supplementary_240618.docx]

**
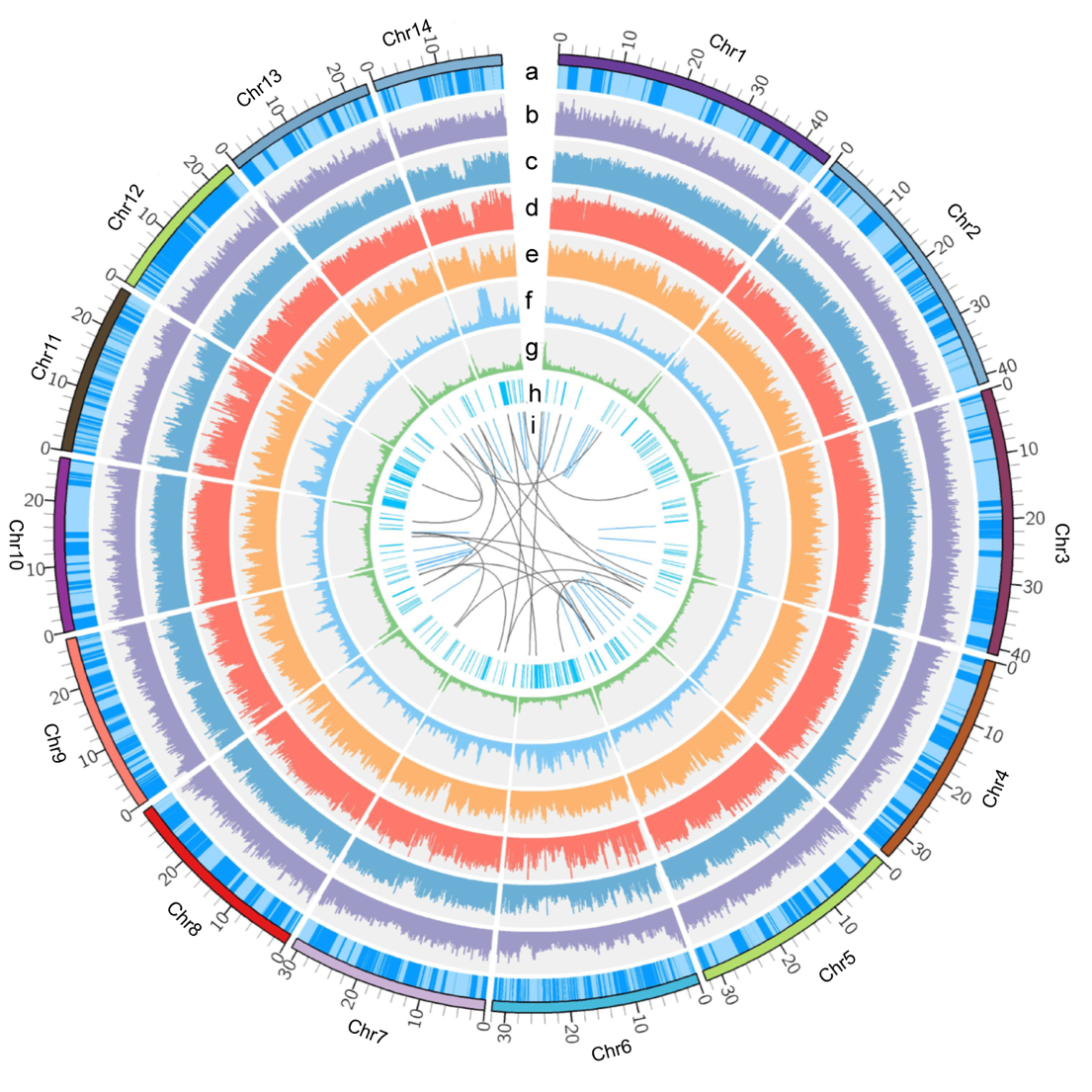
Supplementary Figure**

**Supplementary Figure S1. Circos diagram illustrating the features of the chromosome-level *Pomacea canaliculata* genome.** Chr: chromosome. The outer circle represents the chromosome length of *P. canaliculata*, with units in Mb. Tracks (outer to inner circles) depict the following: **a**, The length of contigs (alternating dark and light colors). **b**, GC content (window size of 200 kb); The sequencing depth of the third-generation (**c**) and the next-generation (**d**) sequencing data. **e,** gene density (gene number per 200 kb window). **f,** Transposable element density (window size of 200 kb). **g,** tandem repeat density (window size of 200 kb). Distribution of segmental (**h**) and tandem duplication gene (**i**).

**
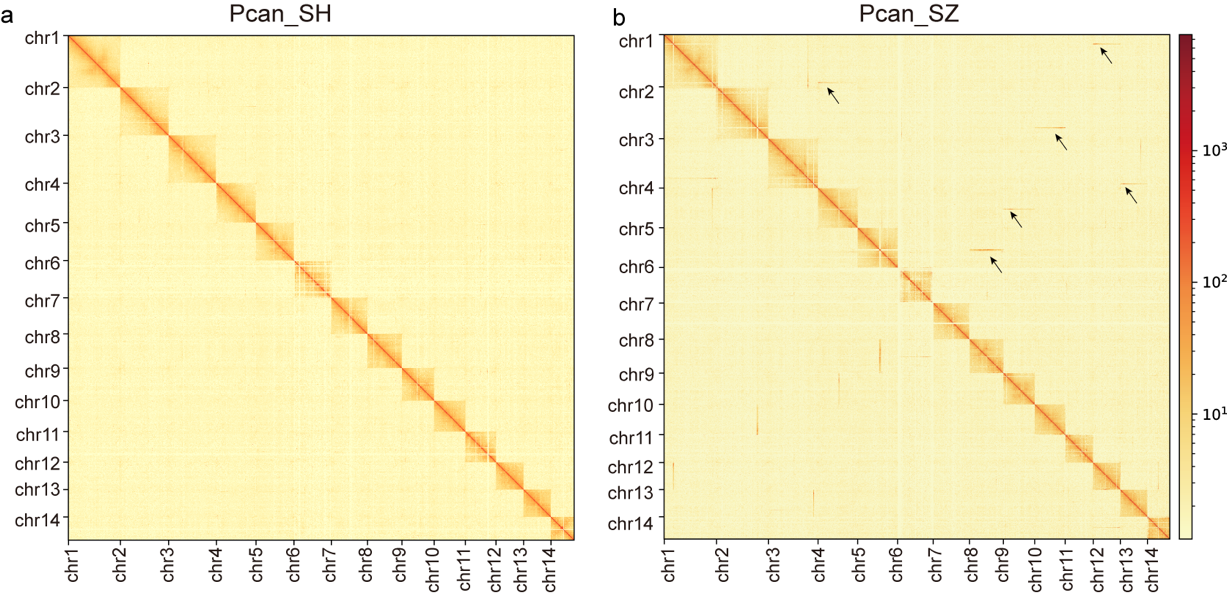
Supplementary Figure S2. Hi-C interaction heatmaps for genome assemblies of *P. canaliculata*.** (**a**) Hi-C interaction heatmap for Pcan_SH genome assembly. The linear sequence of the reference genome assembly is represented on both axes, and the diagonal shows 3D proximity of interacting pairs. No off-diagonal interactions are visible. (**b**) Hi-C interaction heatmap for Pcan_SZ genome assembly. The chromosome names in Pcan_SZ are ordered based on the Pcan_SH chromosome order using the genome synteny analysis. A number of off-diagonal interactions, marked with the black arrows, remain visible. These may arise from either missing links between scaffolds of the same chromosome or from misassembly.

**
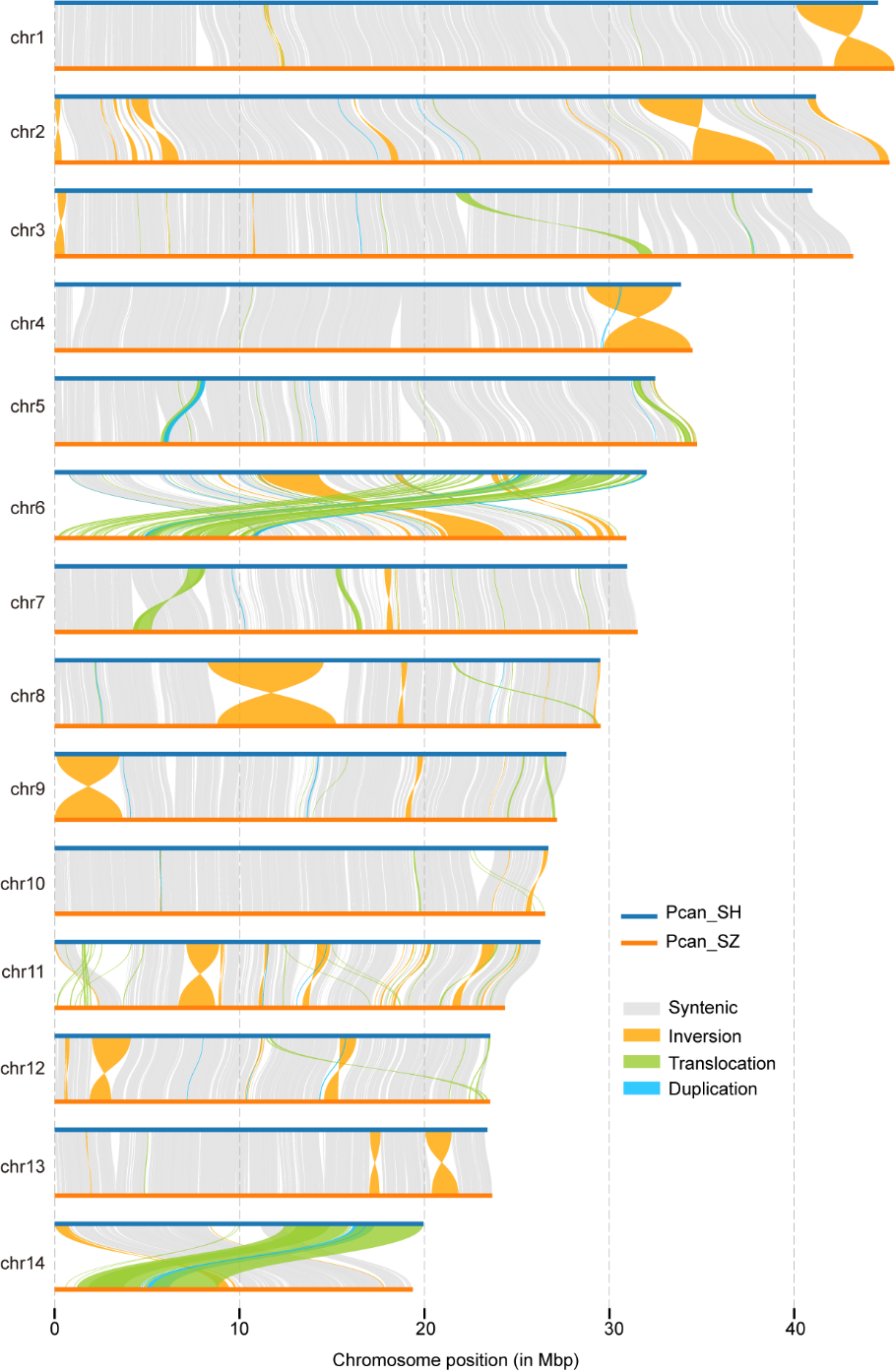
Supplementary Figure S3. Visualization of chromosome synteny and rearrangement between the two assemblies of *P. canaliculata*.** Horizontal blue line represents Pcan_SH, and the orange line represents Pcan_SZ.

**
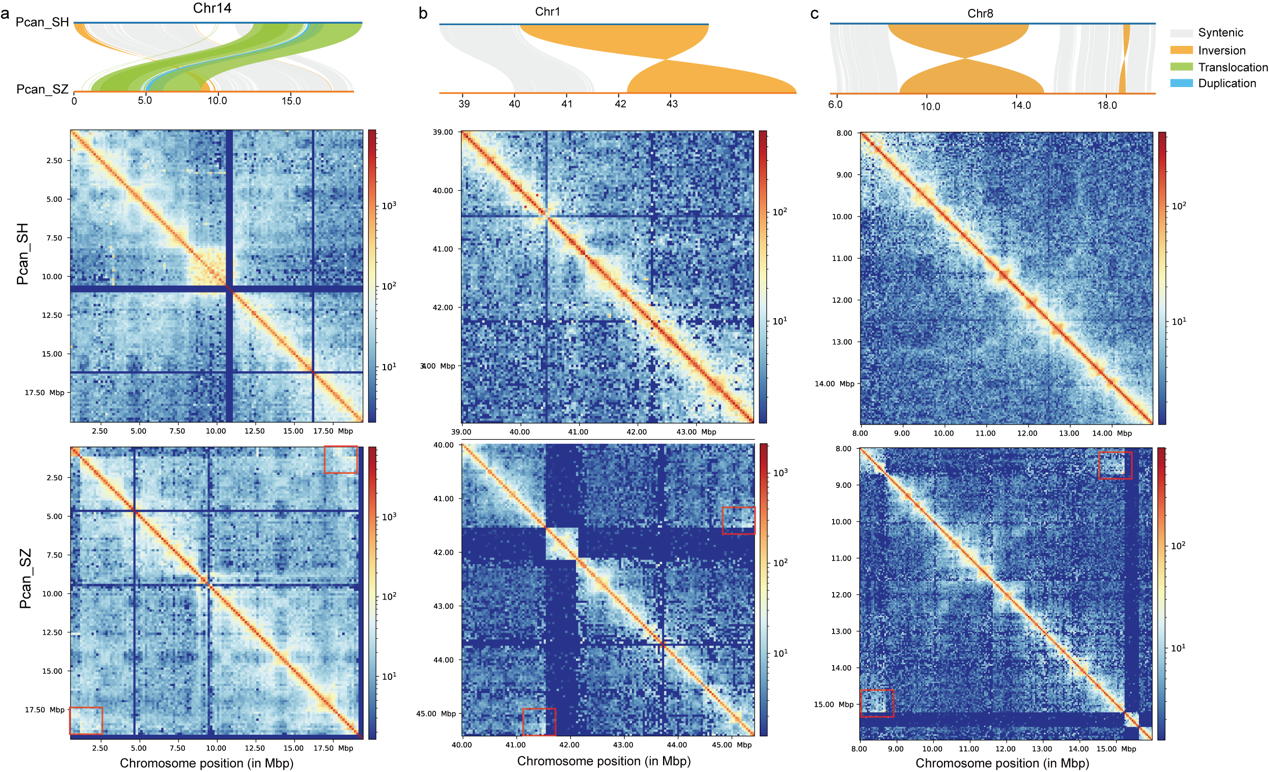
Supplementary Figure S4. Hi-C validation for translocation and inversions between two genome assemblies of *P. canaliculata*.** (**a**) Detailed view of intrachromosomal translocation on the chromosome 14. (**b**) Detailed view of the inversion spanning the regions from approximately 39 Mbp to 45 Mbp on chromosome 1. (**c**) Detailed view of the inversion spanning the regions from approximately 6.0 Mbp to 20 Mbp on chromosome 8. The translocation and inversions are supported by the high-density contacts pointed by the red square in Hi-C heatmaps generated from Pcan_SZ Hi-C reads aligned to the Pcan_SZ genome (bottom), while no corresponding contract is found in the Pcan_SH Hi-C heatmaps (middle). These results indicate accurate genome assembly of Pcan_SH.

**Supplementary**
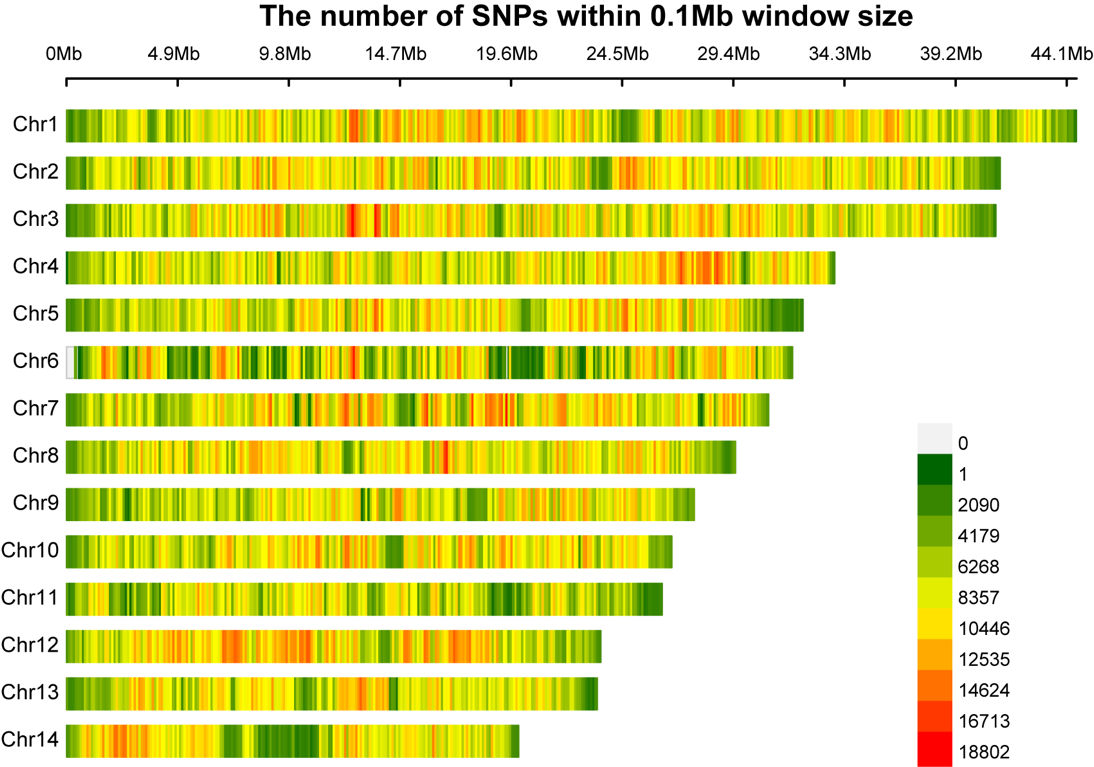
**Figure S5. SNP density plot chromosome wise representing number of SNPs within 0.1 Mb window size.** The horizontal axis shows the chromosome length (Mb), the different color depicts SNP density.


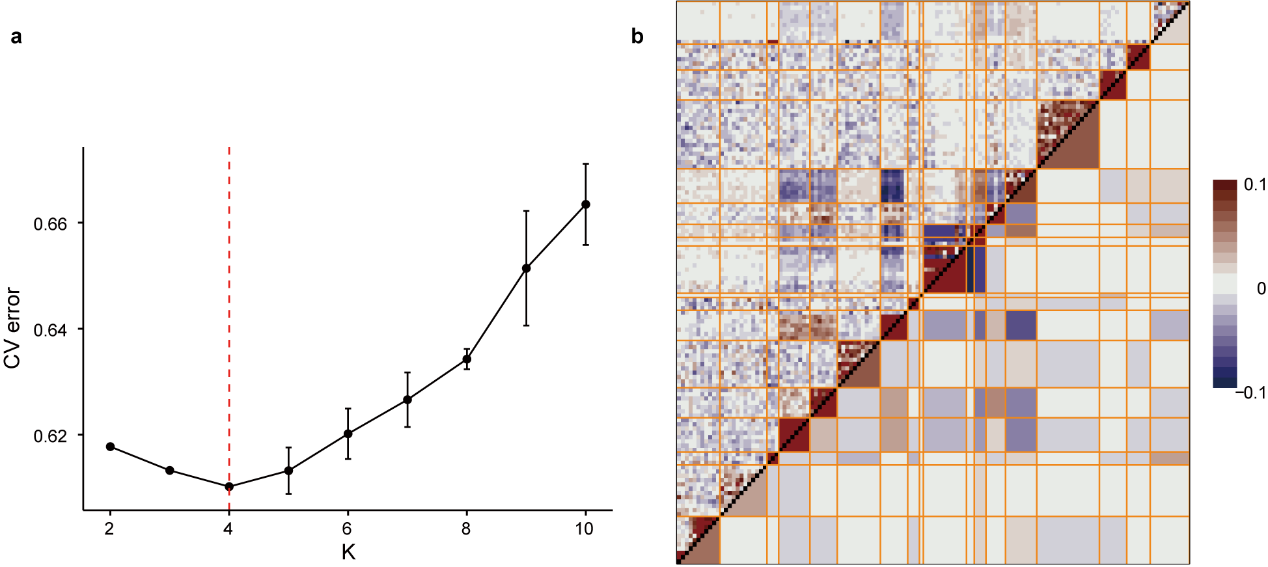


**Supplementary Figure S6. Evaluation for the population structure inferred with Admixture. a,** Plot of ADMIXTURE cross validation error from K=2 through K=10. We chose K=4 as the best K, due to the value that minimizes the error. **b,** Evaluation of the admixture proportions under the bets K=4 model inferred by using the evalAdmix program. The upper diagonal shows the correlation of residuals between individuals, and the lower the mean correlation within populations.

**Supplementary
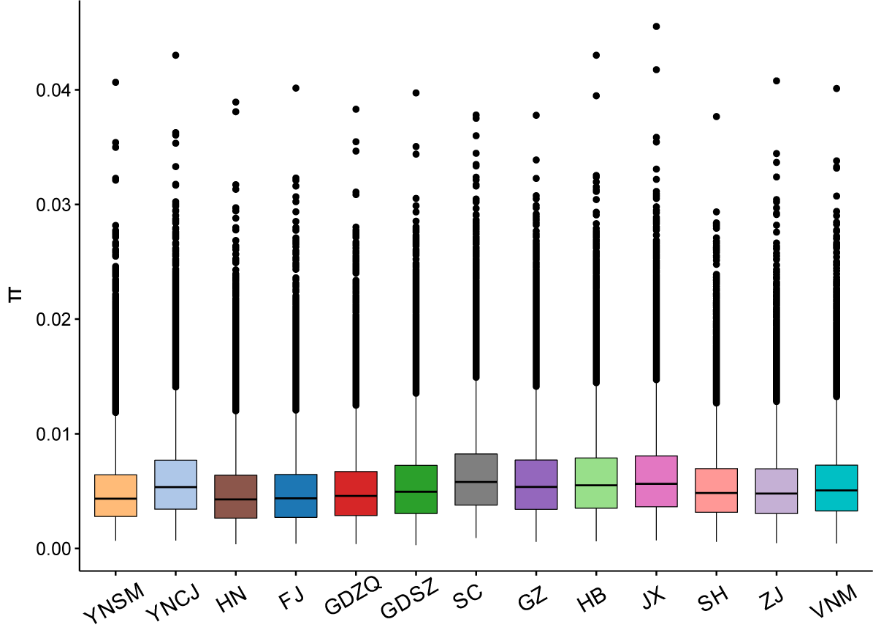
Figure S7. Nucleotide diversity (π) estimated for each population of *P. canaliculata***. Populations with samples less than 3 were removed.

**Supplementary
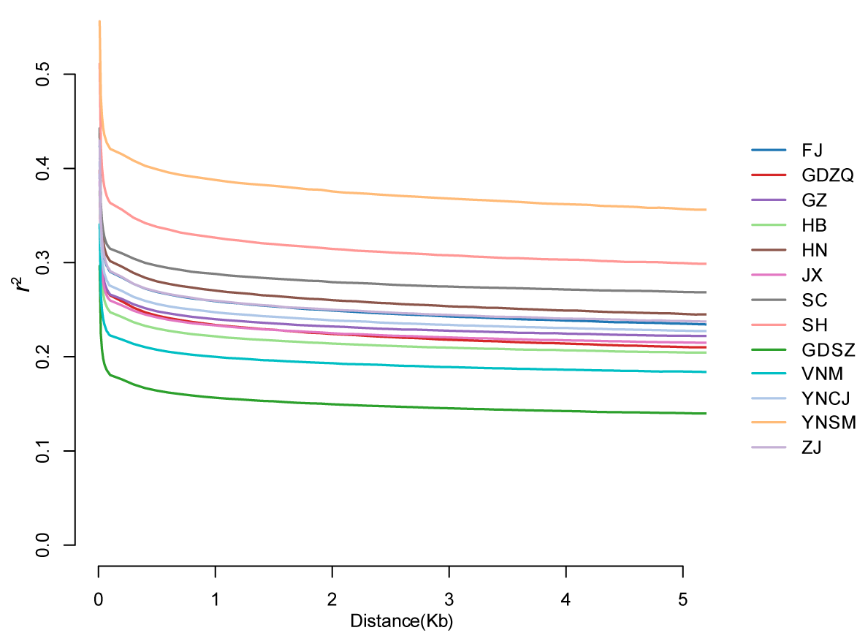
Figure S8. Linkage disequilibrium decay estimated for different populations of *P. canaliculata*.** Populations with samples less than 3 were removed.

**
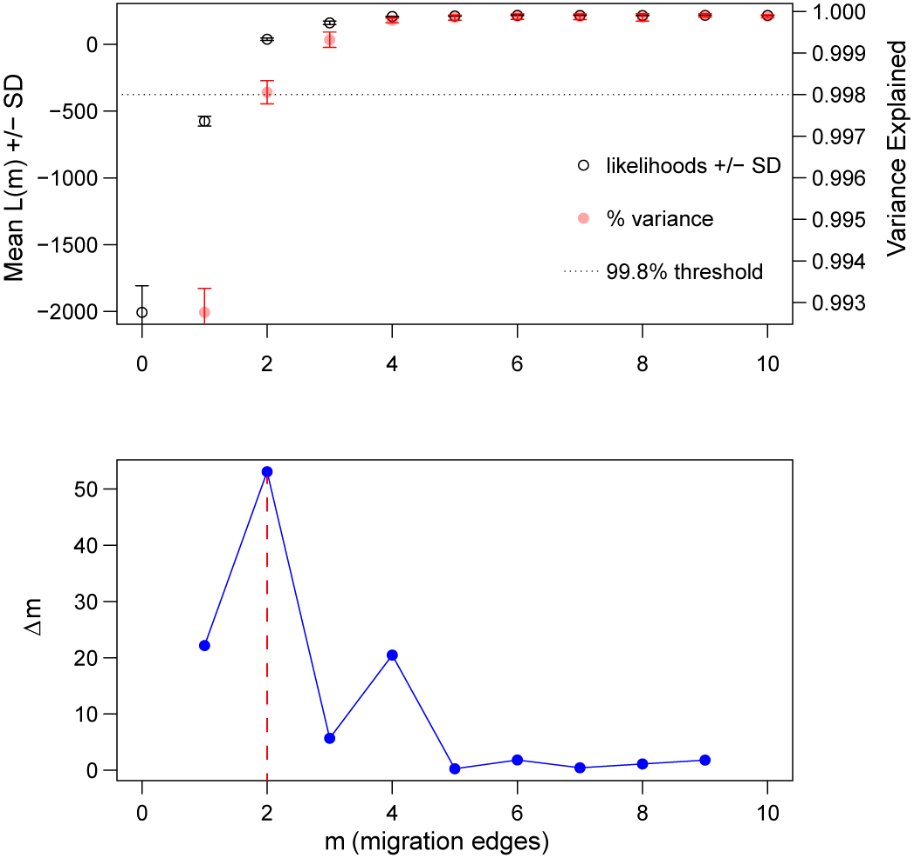
Supplementary Figure S9.** Determination of the optimal number of migration edges between 0 and 15 in TreeMix using the (**a**) log-likelihood values and percentage of variance explained, and (**b**) the second order rate of change in the log-likelihood (Evanno) method.


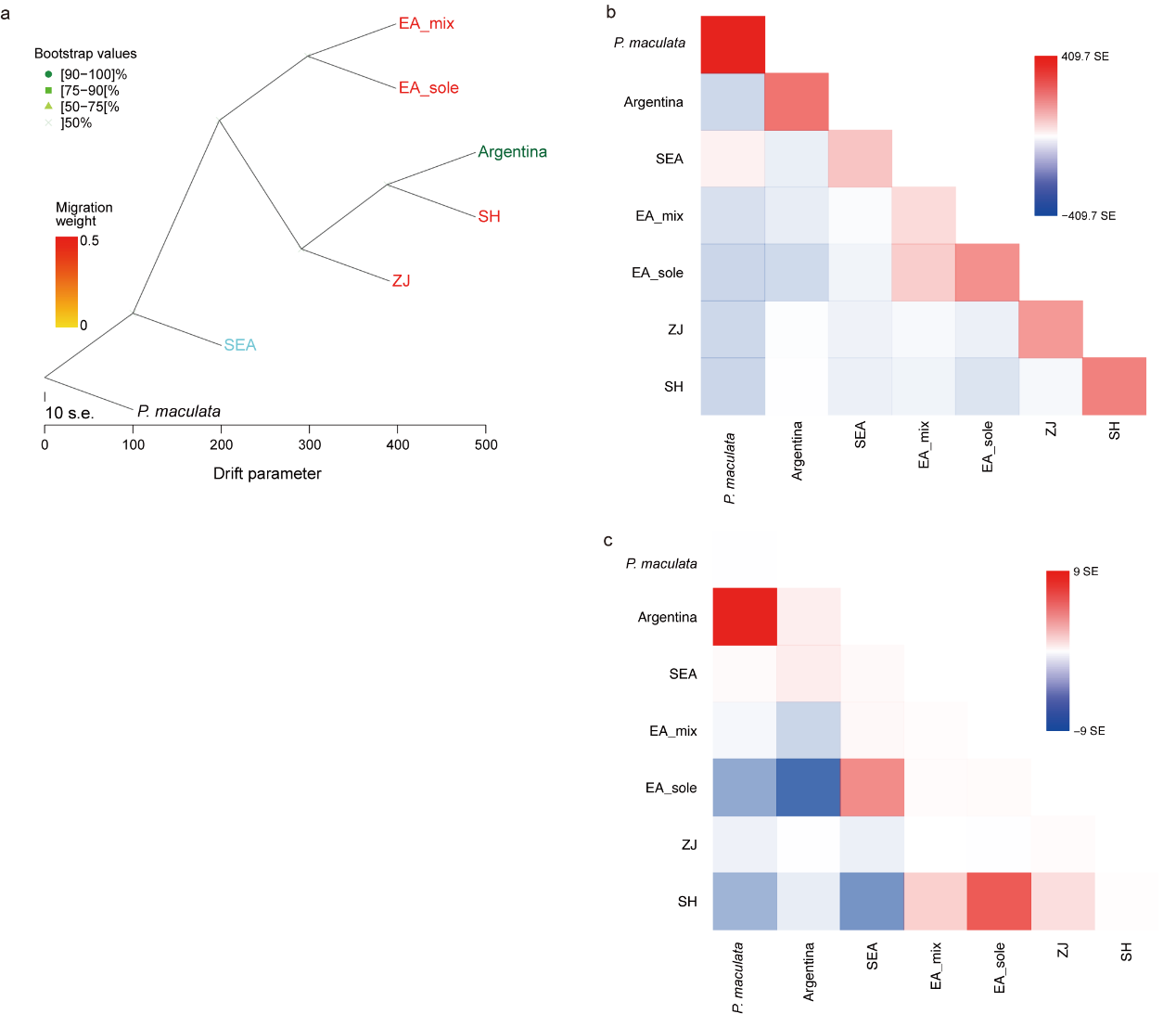


**Supplementary Figure S10. TreeMix-inferred population tree and Residual fits for different migration events.** **a,** TreeMix consensus tree and bootstraps representing the relationships among populations for no migration edge (M0). Residual fit of the observed versus the predicted squared allele frequency difference, expressed as the number of SE of the deviation for M0 (**b**) and M7 (**c**). SE values are represented by colors according to the palette on the right. Residuals above zero indicate populations that are more closely related to each other in the data than in the best-fit tree and have potentially undergone admixture. Negative residuals represent populations that are less closely related in the data than represented in the best-fit tree.


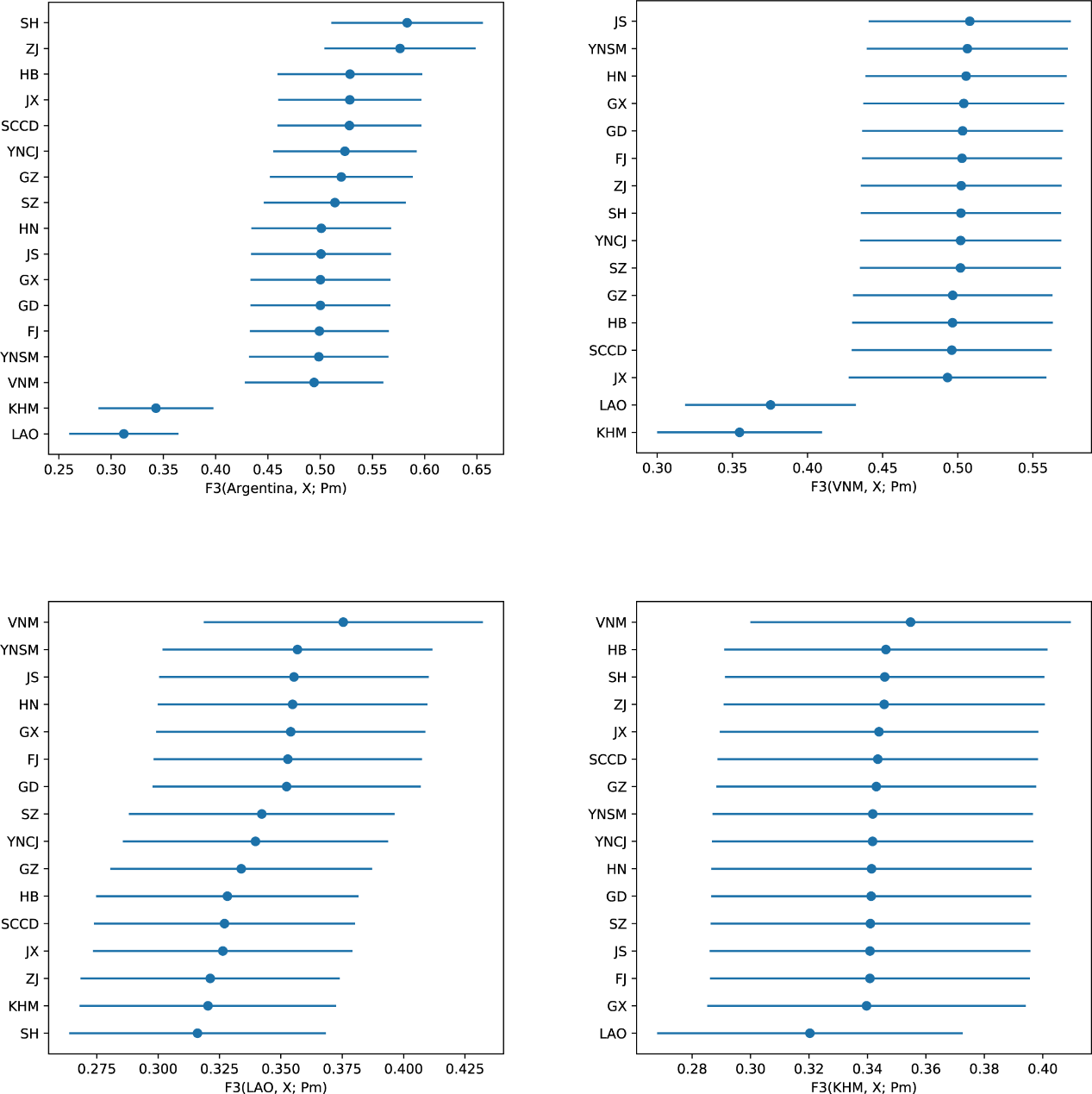


**Supplementary Figure S11.** **Outgroup *f*3 statistics between the Argentina (a), VNM (b), LAO (c), KHM (d) and all other populations of *P. canaliculata*.** Outgroup *f*3 statistics in the form *f*3 (X, Y; *P. maculata*) where *P. maculata* was selected as the target population.

**Supplementary
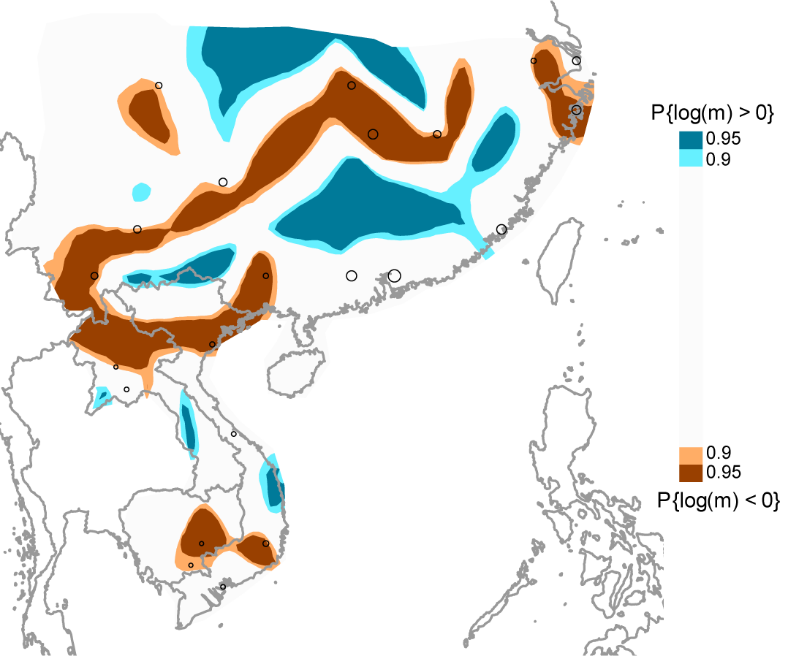
Figure S12. EEMS posterior probability of effective migration (*m*) estimated in EEMS.**

**Supplementary**
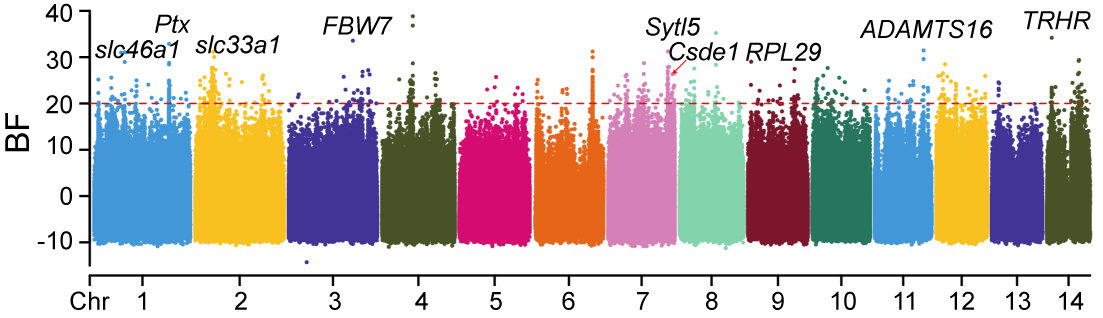
**Figure S13. Genome-wide association with the Min Temperature of Coldest Month covariable using BayPass.** The horizontal red dashed line (BF>20 dB) corresponds to the chosen significance level for genotype-climate association.

**
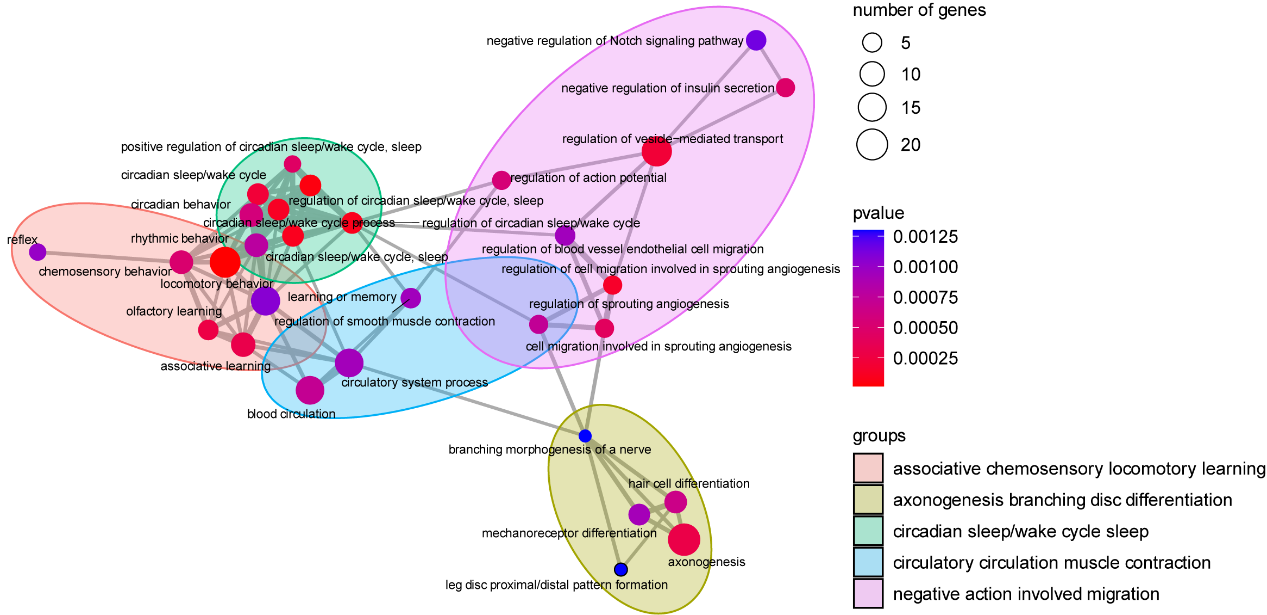
 Supplementary Figure S14. Emapplot of the GO function enrichment with the selected genes associated with the Min Temperature of Coldest Month covariable.** The GO category was grouped using ClusterProfiler R package.

**
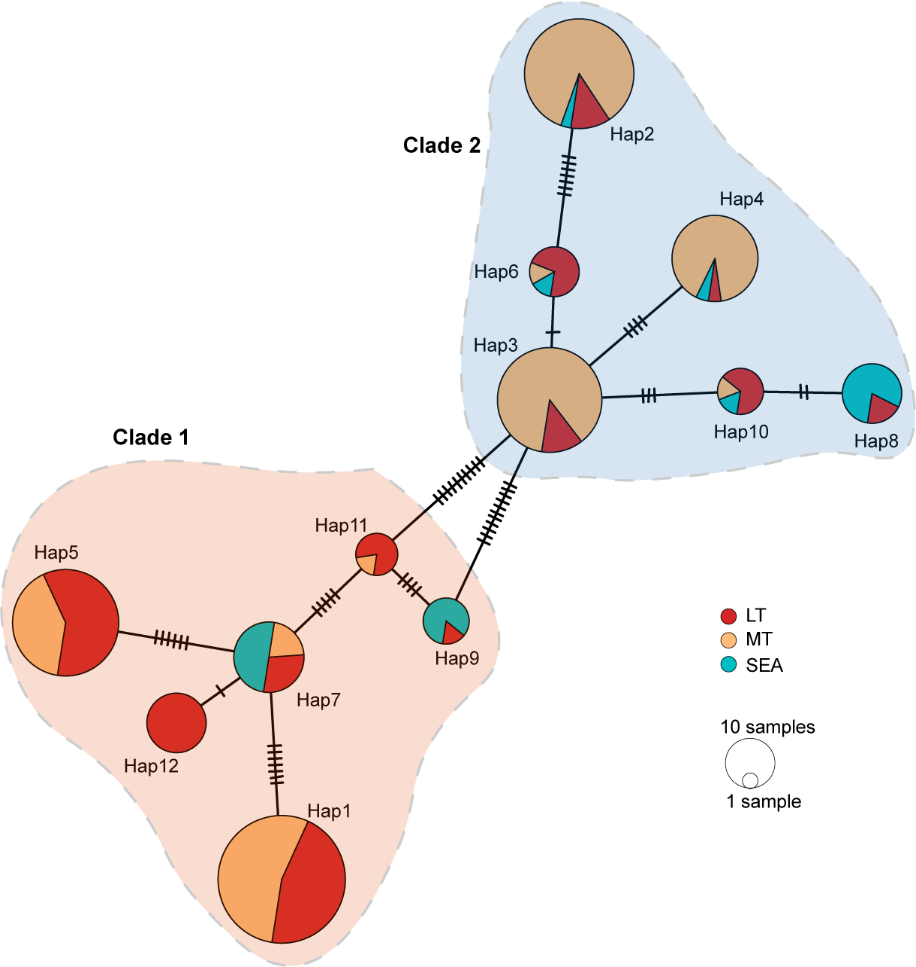
Supplementary Figure S15. Haplotype network of the 5’-UTR in *CSDE1* gene.** We used median-joint network analysis to construct the haplotype network of the 5’-UTR in *CSDE1* gene among all *P. canaliculata* accessions. The haplotypes that were present in a single accession were excluded. The node size and the parallel lines on branches represent the relative haplotype frequency and the number of mutations, respectively. The different colored portions in each node represent the proportion of different *P. canaliculata* accessions within each haplotype.

**Supplementary
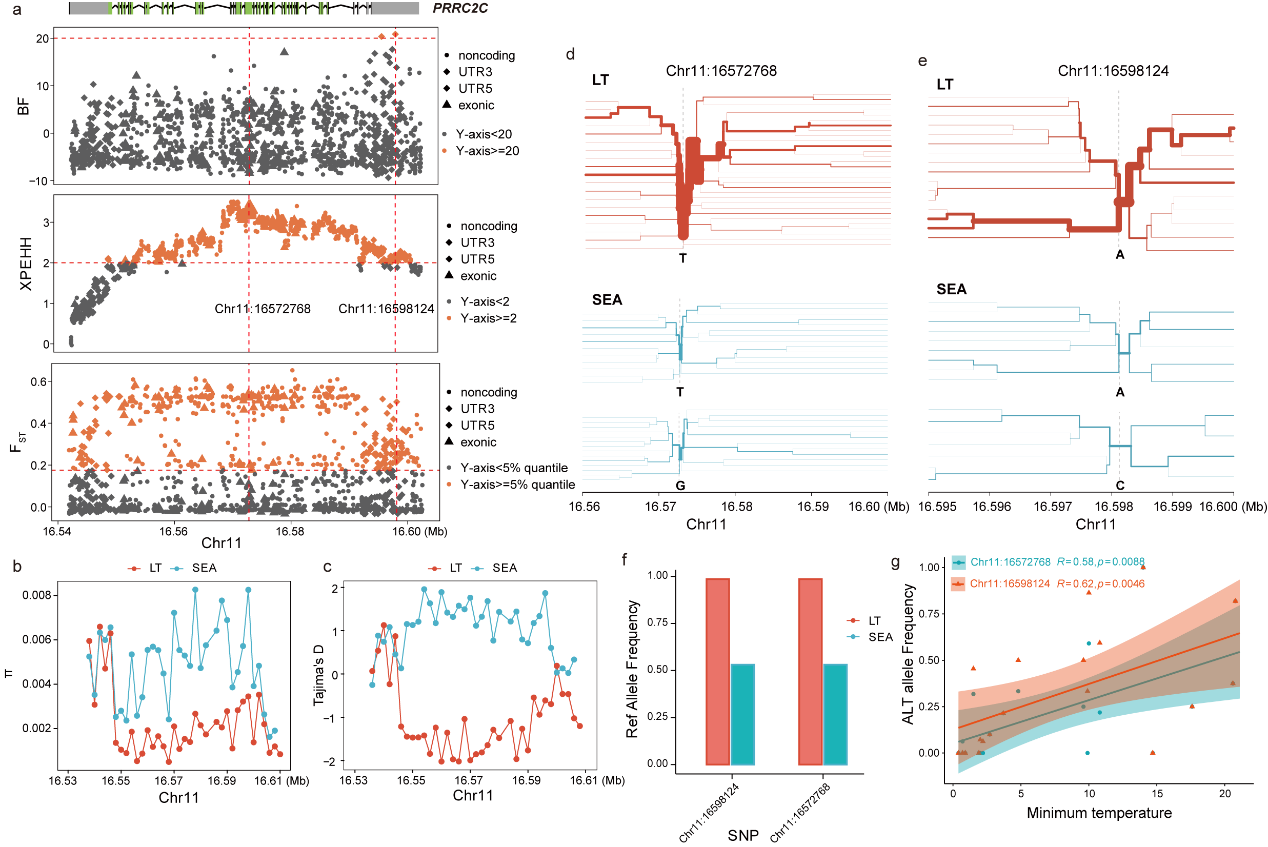
Figure S16. Signatures of positive selection in LT population and Genotype-environmental association on the *PRRC2C* gene. a,** Multiple statistics representing genotype-environmental association and positive selection on the genomic region harboring the *PRRC2C* gene. The y axis presents Bayles factor value (the 1st panel), and the normalized XPEHH (the 2nd panel) and F_ST_ values (the 3rd panel). Circles, triangles and squares denote variants under non-coding, UTR and exonic regions, respectively. Nucleotide diversity (**b**) and Tajima’s *D* statistic (**c**) in the PRRC2C gene for LT and SEA populations. Haplotype bifurcation plots for LT and SEA haplotypes with the peak SNPs of Chr11: g. 16572768 (**d**) and Chr11: g. 16598124 (**e**). Colors of each plot reflect the location of sampling. Labels indicate nucleotides at central position. **f**, Alternate allele frequency of the SNPs (Chr11: g. 16572768 and Chr11: g. 16598124). **g,** The significantly positive correlation between the alternate allele frequency of the SNPs (Chr11: g. 16572768 and Chr11: g. 16598124) and Min Temperature of Coldest Month in different sampling locations.


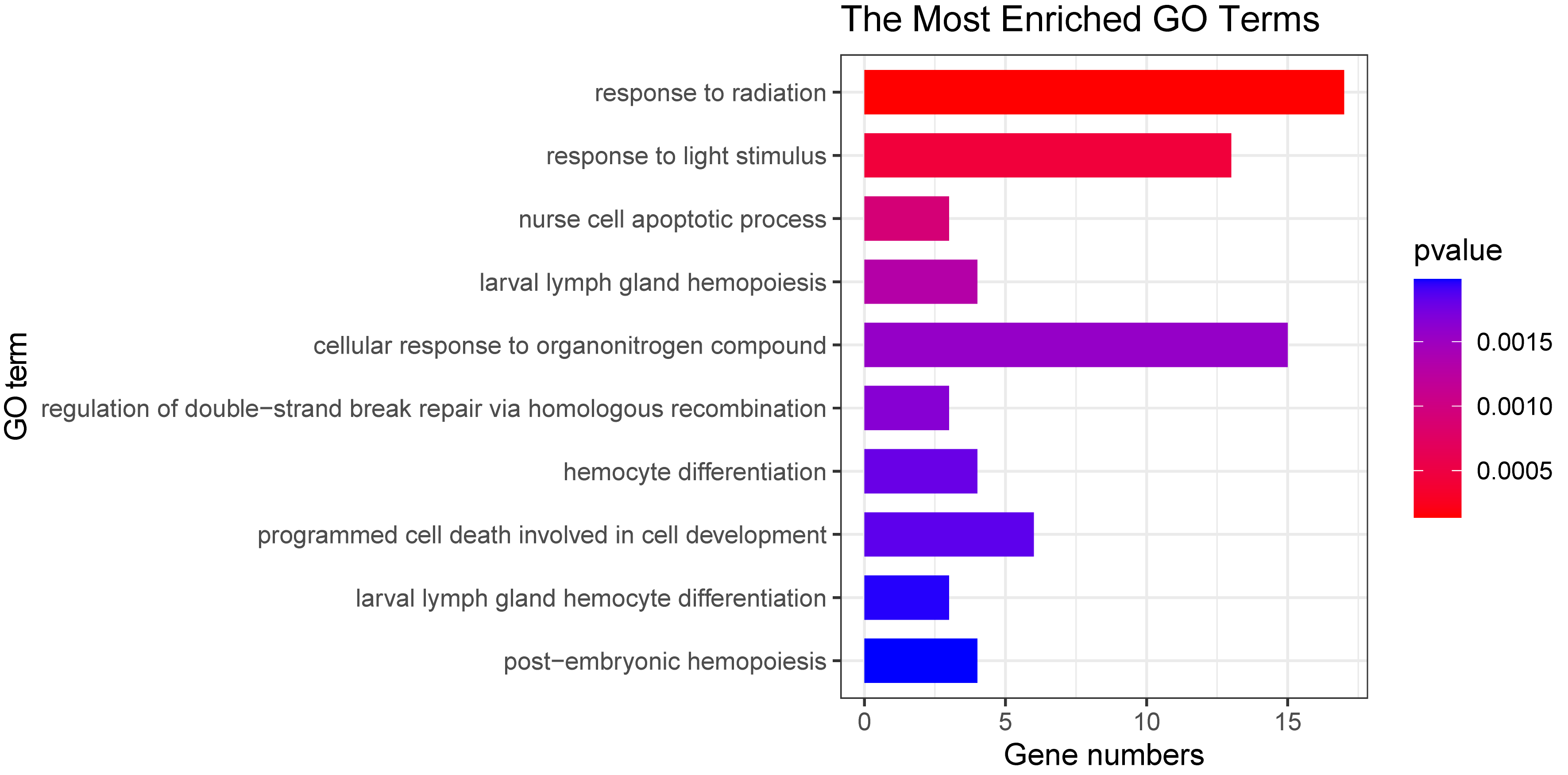


**Supplementary Figure S17. Bar plot of the GO function enrichment with the balancing selected genes in both EA and SEA populations, the top 10 GO terms are showed in here.** The GO category was grouped using ClusterProfiler R package.


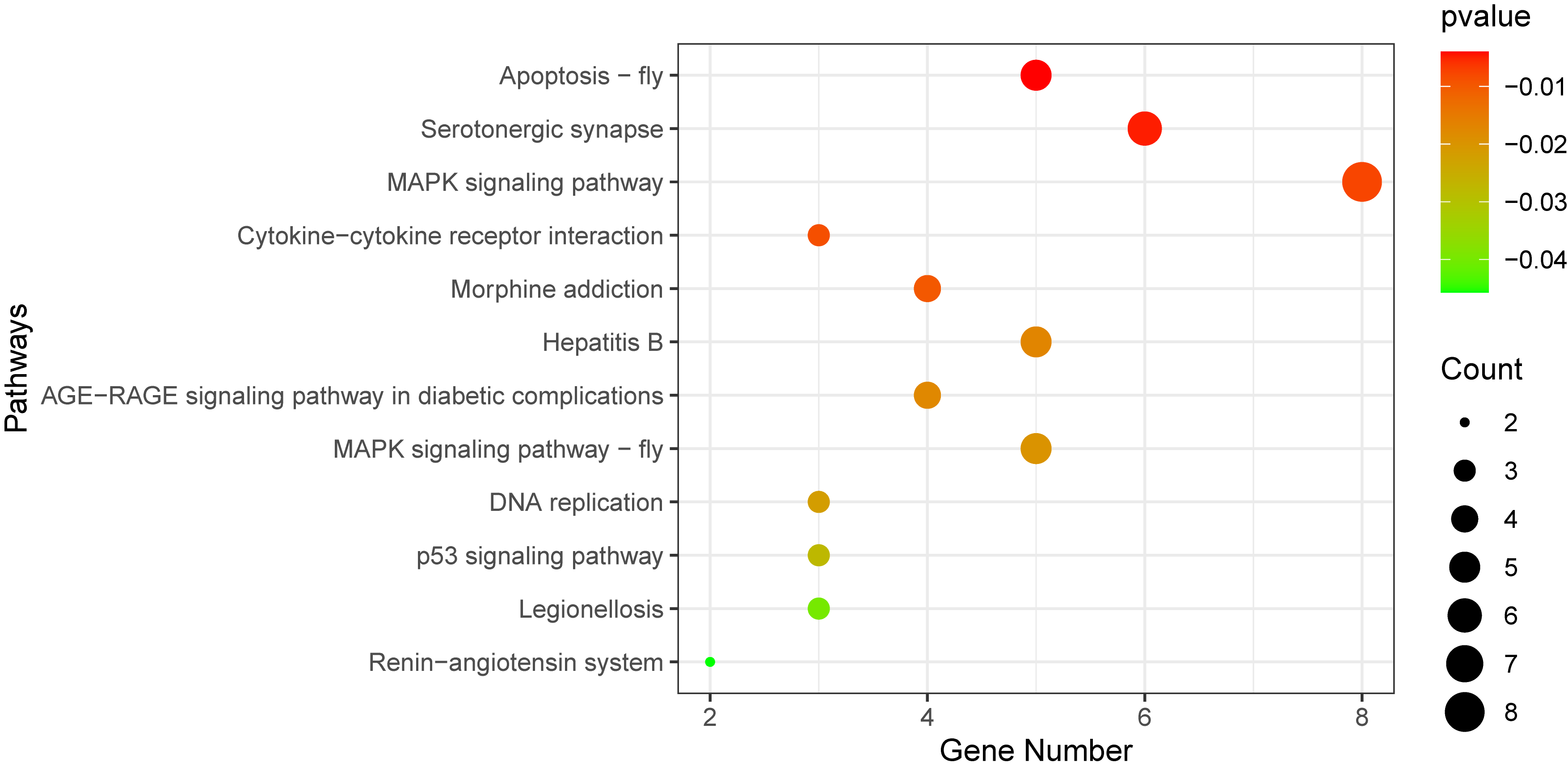


**Supplementary Figure S18. Diagram plot of the KEGG pathway enrichment with the balancing selected genes in both EA and SEA populations.** The pathway category was grouped using ClusterProfiler R package.


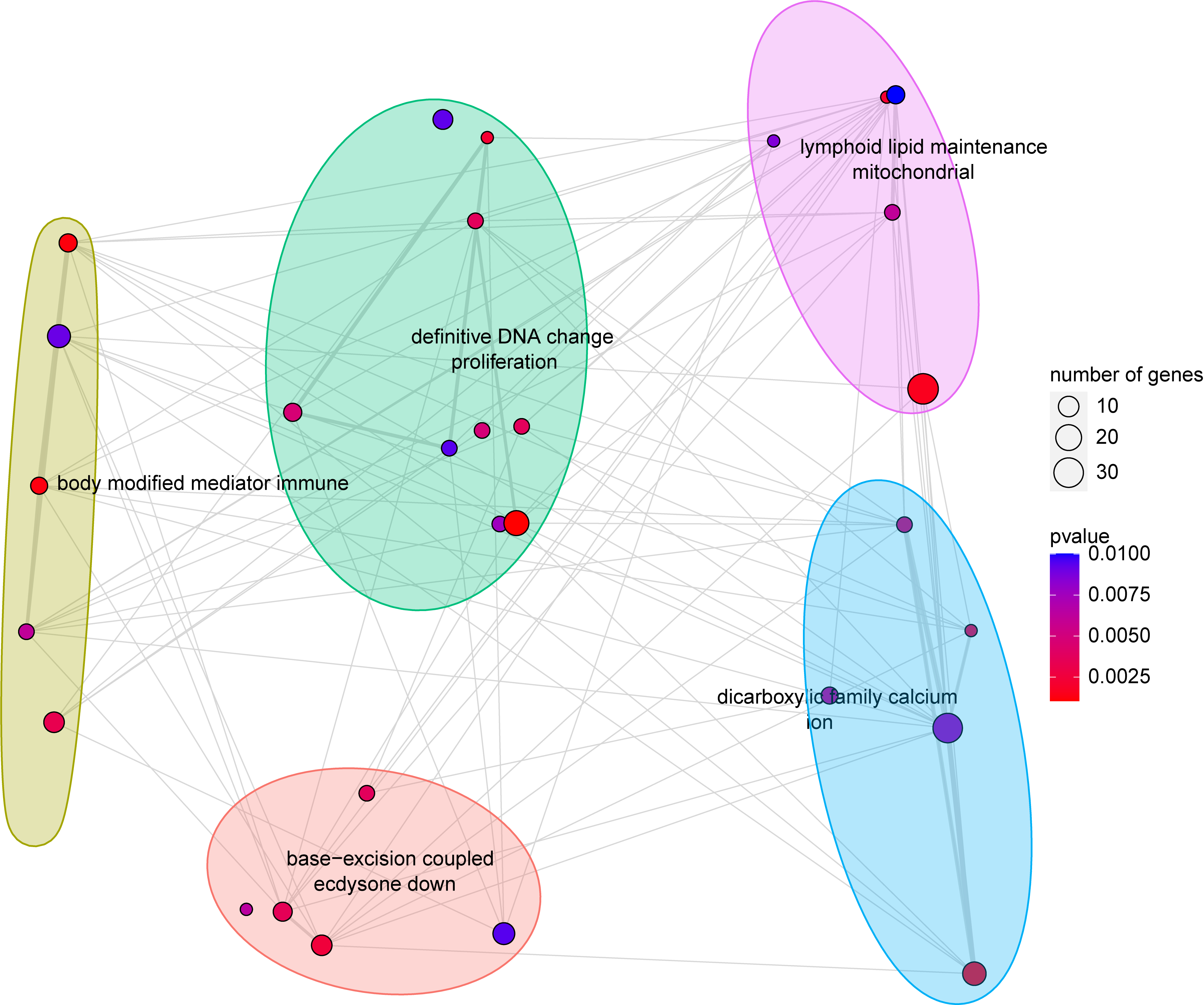


**Supplementary Figure S19. Emapplot of the GO function enrichment with the balancing selected genes in EA population.** The GO category was grouped using ClusterProfiler R package.

**
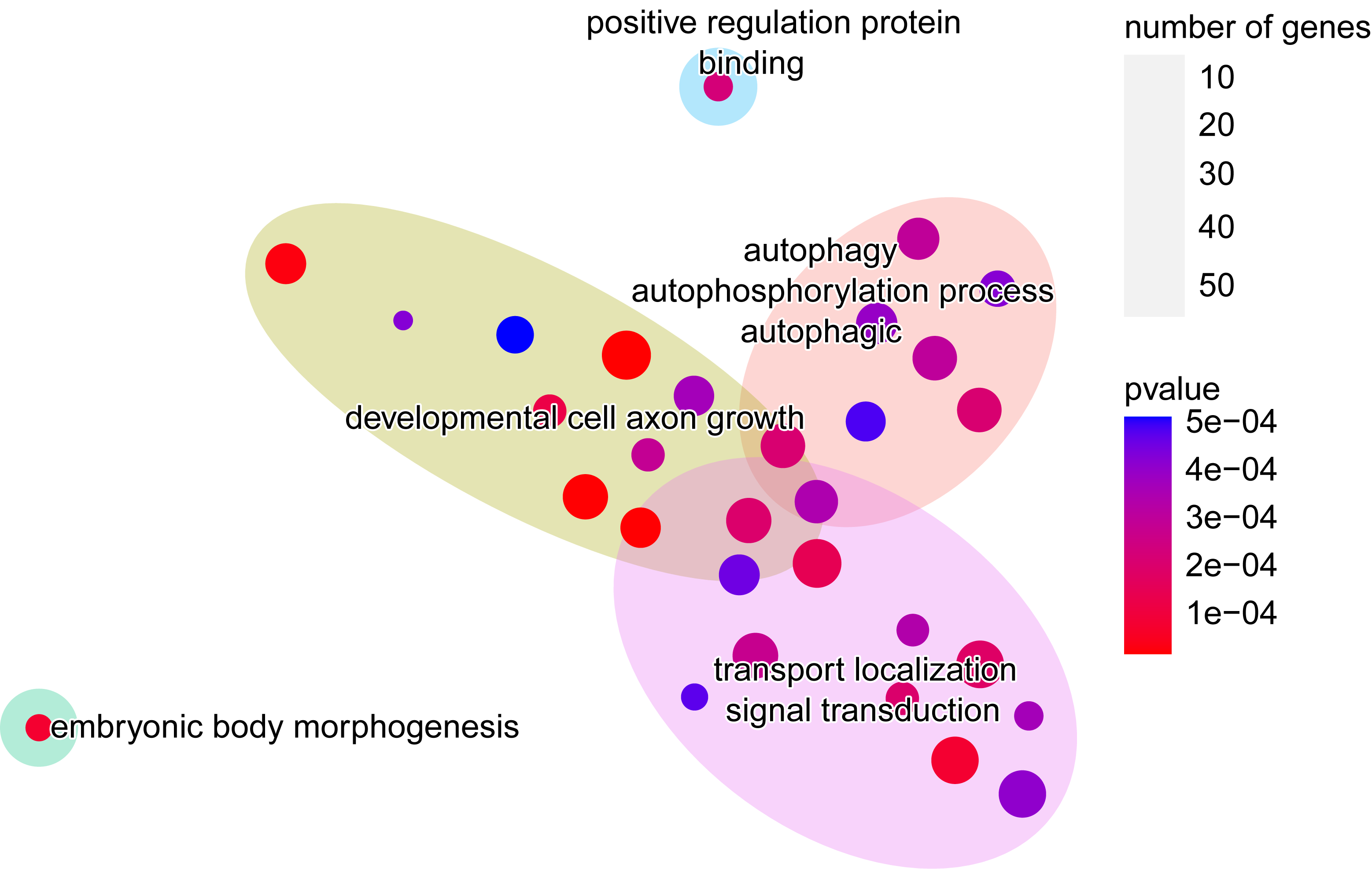
**

**Supplementary Figure S20. Emapplot of the GO function enrichment with the balancing selected genes in SEA population.** The GO category was grouped using ClusterProfiler R package.

**
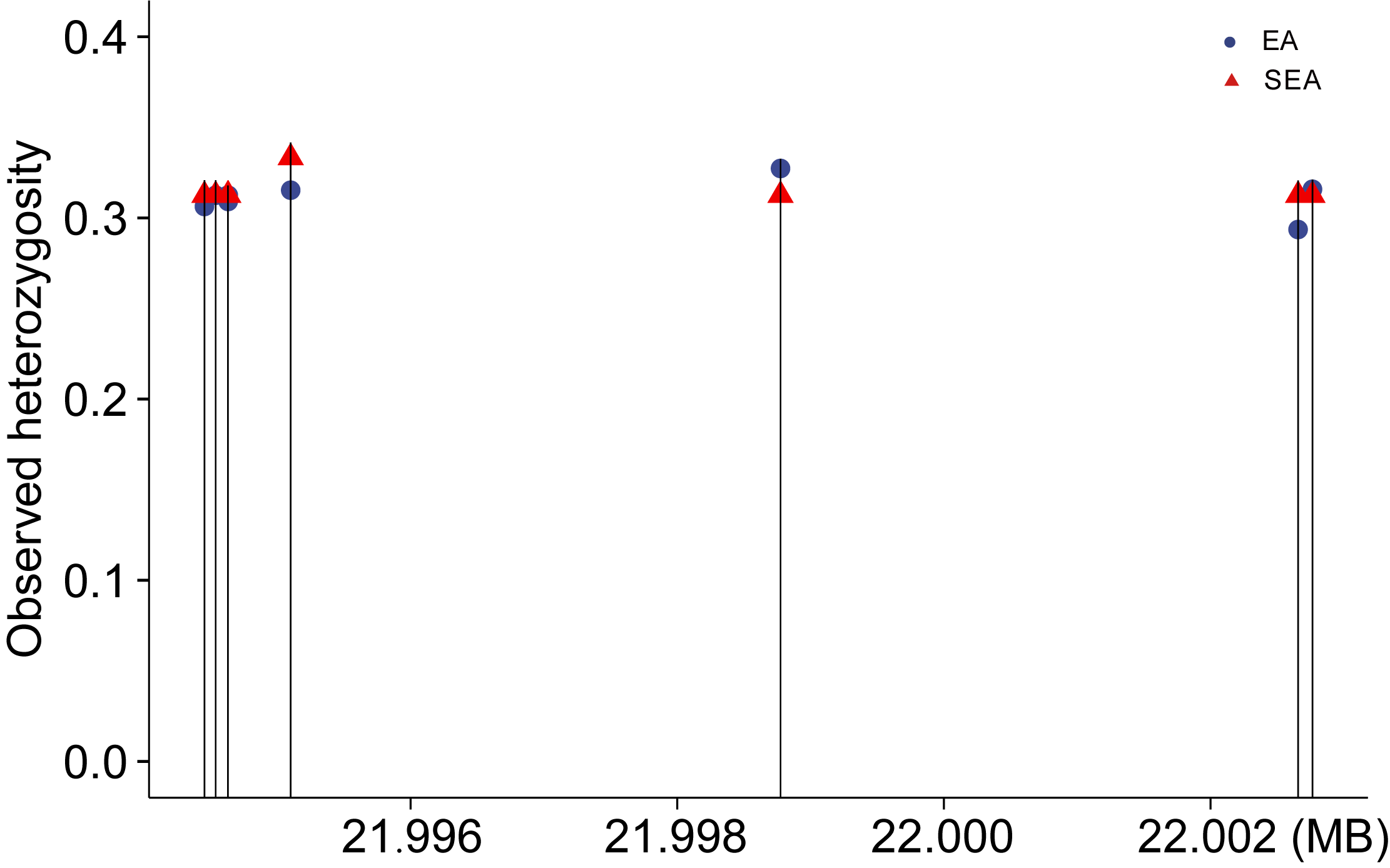
**

**Supplementary Figure S21. Diagram showing the nonsynonymous SNPs and heterozygous genotype frequency in the *E4F1* gene region**. The functions of SNPs were defined and predicted by Annovar.

**
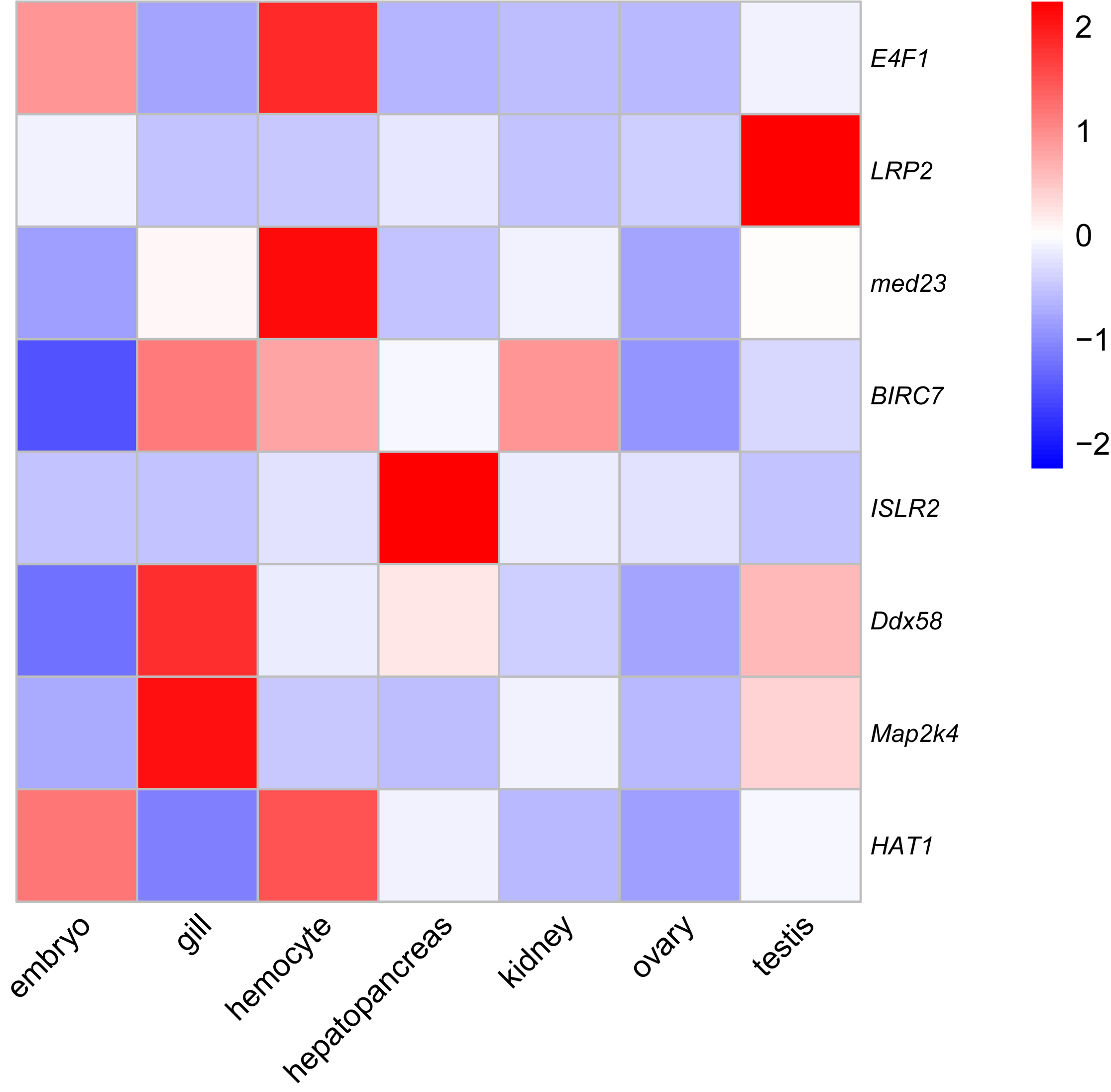
**

**Supplementary Figure S22. Expression profile of the balancing selected genes** **in different adult tissues of *P. canaliculata.*** The sequencing data were downloaded in NCBI under PRJNA427478 accession.

**Supplementary Table**

**Supplementary Table 1. Summary statistics of assembly and annotation for the *Pomacea canaliculata* genomes.**

|  | **Pcan_SH** | **Pcan_SZ** |
| --- | --- | --- |
| Assembly size (Mb) | 440.8 | 440.1 |
| GC content (%) | 40.61 | 40.61 |
| Scaffold number | 1,012 | 24 |
| Contig number | 2,235 | 746 |
| Scaffold N50 (Mb) | 31.4 | 31.5 |
| Contig N50 (Mb) | 1.16 | 1.07 |
| Sequence anchored to chromosomes (%) | 98.11 | 97.46 |
| Complete BUSCO (%) | 96.1 | 98.0 |
| Number of genes | 24,832 | 24,194 |

**Supplementary Table 3. Summary statistics of sequencing information for *P. canaliculata* accessions.**

| **Location** | **Sample Sequenced** | **Passed quality control** |
| --- | --- | --- |
| FJ | 11 | 11 |
| GDZQ | 12 | 12 |
| GX | 12 | 3 |
| GZ | 10 | 8 |
| HB | 9 | 7 |
| HN | 12 | 11 |
| JS | 6 | 3 |
| JX | 10 | 7 |
| SC | 12 | 5 |
| SH | 15 | 5 |
| GDSZ | 16 | 16 |
| ZJ | 11 | 10 |
| YNCJ | 9 | 7 |
| YNSM | 12 | 6 |
| Vietnam | 11 | 11 |
| Laos | 3 | 3 |
| Cambodia | 2 | 2 |
| Total | 173 | 130 |

**Supplementary Table 5. Top 20 candidate windows associated with the Min Temperature of Coldest Month covariable using BayPass.**

| **Chr** | **Start** | **End** | **nSNPs** | **Outlier SNPs** | **Max BF** | **Genes** |
| --- | --- | --- | --- | --- | --- | --- |
| 4 | 14312286 | 14332622 | 20336 | 9 | 38.83 | Pca0068740; Pca0068750; Pca0068760 |
| 8 | 16735327 | 16745374 | 10047 | 2 | 35.21 | Pca0136160 |
| 14 | 2184514 | 2194514 | 10000 | 1 | 34.18 | *TRHR* |
| 3 | 29670097 | 29680097 | 10000 | 1 | 33.57 | Pca0055680; *FBW7* |
| 1 | 34511981 | 34583307 | 71326 | 21 | 32.88 | Pca0016110; *Ptx* |
| 11 | 22556080 | 22575212 | 19132 | 5 | 31.45 | *ADAMTS16* |
| 7 | 27697398 | 27724807 | 27409 | 5 | 31.24 | *Sytl5* |
| 6 | 26961361 | 26986164 | 24803 | 60 | 31.23 | Pca0110060 |
| 2 | 8317973 | 8342201 | 24228 | 7 | 31.15 | *slc33a1*; *ASTE1* |
| 1 | 14040897 | 14050898 | 10001 | 2 | 31.06 | *slc46a1* |
| 2 | 8780439 | 8790439 | 10000 | 1 | 30.04 | *KCNAB2* |
| 7 | 30664456 | 30674456 | 10000 | 1 | 29.53 | Pca0126990 |
| 14 | 14819679 | 14829689 | 10010 | 2 | 29.37 | Pca0215590 |
| 14 | 14680349 | 14690349 | 10000 | 1 | 29.20 | *U2surp*; *IPO5* |
| 9 | 1436129 | 1446129 | 10000 | 1 | 29.02 | *RPL29* |
| 7 | 16541136 | 16551136 | 10000 | 1 | 28.71 | tachykinin-like peptides receptor 86C |
| 12 | 4074778 | 4084778 | 10000 | 1 | 28.48 | Pca0186660 |
| 7 | 28151634 | 28167412 | 15778 | 2 | 27.91 | pyruvate dehydrogenase E1; pyruvate dehydrogenase E1; Pca0125860; *Tmtc1* |
| 7 | 27631778 | 27647893 | 16115 | 20 | 27.82 | *ATG5*; *chmp2a*; *Csde1*; *Prmt3* |
| 2 | 8021596 | 8036555 | 14959 | 6 | 27.75 | *TMED6* |

**Supplementary Table 8. List of protein-coding genes identified by both bayPass results and selective sweep analyses.**

| **geneID** | **Alias** | **Genename** | **log2FoldChange_cold** | **padj_cold** |
| --- | --- | --- | --- | --- |
| Pca0009210 | ccnf | Cyclin-F | 0.259880262 | 0.138964166 |
| Pca0019080 | Gyc32E | Guanylate cyclase 32E | -0.828757108 | 2.23E-23 |
| Pca0032770 | pqn-25 | Glutamine/asparagine-rich protein pqn-25 | -0.534046481 | 0.013319776 |
| Pca0043460 | CARS | Cysteine--tRNA ligase, cytoplasmic | 0.099099702 | 0.45568758 |
| Pca0055660 | FBXO8 | F-box only protein 8 | 0.012214789 | 0.943002888 |
| Pca0105160 | - | Dopamine D2-like receptor | 2.849950894 | 5.75E-46 |
| Pca0128850 | CUBN | Cubilin Precursor | - | - |
| Pca0159960 | Pka-C1 | cAMP-dependent protein kinase catalytic subunit 1 | -0.416370303 | 1.39E-08 |
| Pca0179630 | PRRC2C | Protein PRRC2C | -0.6940762 | 6.92E-09 |

**Supplementary Table 12. Primers used in this study.**

| **Genes** | **Primers** | **Sequences (5’-3’)** |
| --- | --- | --- |
| *β-Actin* | qActin-F | TCACCATTGGCAACGAGCGAT |
|  | qActin-R | TCTCGTGAATACCAGCCGACT |
| *csde1* | qcdse1-F | AACAAGGACACAGAGACAG |
|  | qcsde1-R | CGGATTACCACACGAACA |
