# Supplementary material for: Whole-genome sequencing of the invasive golden apple snail Pomacea canaliculata from Asia reveals rapid expansion and adaptive evolution
Source: Gigascience. 2024 Sep 23;13:giae064. doi: 10.1093/gigascience/giae064 (PMC11417965; doi:10.1093/gigascience/giae064)
Supplement: giae064_Response_to_Reviewer_Comments_Original_Submission [file giae064_response_to_reviewer_comments_original_submission.pdf]

Author's Response To Reviewer Comments

Response to the comments of Reviewer #1

Reviewer #1: This is a remarkably thorough paper that combines whole genome assembly and annotation, population genomics, and gene expression data for the golden apple snail Pomacea canaliculata. The authors identify signals of demographic history and natural selection resulting from this invasive species' spread across Asia. They pinpoint specific genes adapted to temperature and other stressors that these snail populations have encountered. Overall, this is an impressive and sweeping genome-scale study on a poorly-known non-model species.
The authors assemble and annotate a genome for this snail species. However, the genome of this species was already published several years ago in Gigascience (Liu et al. 2018), and it is a high-quality genome featuring chromosome-level assembly. So it was not evident why they generated a new genome. It is quite plausible that their new genome is better than the published genome in various respects, but in the manuscript there is no mention of any kind of comparison between the new and old genome. While they do cite the previous genome paper (reference 25), it is only in the context of RNA-Seq data, so there is no indication in the manuscript that a published genome even exists. A brief justification and comparison is warranted. When they mention a "reference genome" (e.g. line 61), I am assuming they mean their new genome not the old one, but this should be clarified. Furthermore, given that genomic resources for mollusks are relatively sparce (see Davison & Neiman 2021), it wouldn't hurt to have a sentence or two on how this new assembly contributes to the broader set of published mollusk genomes.

Response: Thanks for your valuable feedback on our manuscript. We appreciate your insightful comments and suggestions. We agree that a more explicit justification and comparison between genome and the genome that was previously published (Lie et al., 2018) would enhance the clarity of our work.
After comparing our newly assembled P. canaliculata genome (Pcan SH, which was collected from Shanghai, China) to the previously published genome (Pcan SZ, collected from Shenzhen city), we discovered that both genomes exhibited comparable integrity and continuity, with the Pcan SH having a slightly longer Scaffold/Contig N50 size and a better BUSCO (Table S1). In particular, there is a noticeably deeper read depth of Hi-C sequencing data in the recently assembled genome Pcan SH. Taking advantage of the high coverage Hi-C data, we were able to observe that while several off-diagonal interactions were present in the previously published genome, none of them could be seen in our newly assembled Pcan SH genome, suggesting a more precise and accurate assembly process for Pcan_SH (Figure S2). It turned out that our new reference genome facilitated identifying several chromosomal rearrangements that have not been reported before with the older assembly Pcan_SZ (Figure S3). In particular, several intra-chromosomal translocations, inversions, and Hi-C contacts were determined by comparing the two assemblies. These results indicated some probable mis-assembly in previously published genome Pcan_SZ (Figure S4). These findings underscore the improvements in our assembly and highlight the impact of increased Hi-C sequencing depth in resolving chromosomal structures accurately.
Following the suggestion of the reviewer, we have added the above content to the revised manuscript, i.e., we have included a section (the line 65-92) dedicated to comparing the two genomes, highlighting improvements or unique features of our assembly. We have also clarified that we are specifically addressing our newly generated genome Pcan_SH when referring to a "reference genome". Thank you again for your constructive comments.
The selection scans are well executed and generally robust. However, I had a few concerns about their balancing selection analysis. While it is true that a mild excess of heterozygosity surpassing Hardy-Weinberg expectations is a telltale sign of balancing selection, it is virtually unheard of in any species that selection coefficients would be so strong as to push heterozygosity for a biallelic polymorphism to 80-100%, as in Figure 5e, Figure S17d, and Figure S18d. To have this effect at several different unlinked genes in the same populations is basically impossible from a genetic load perspective. A much more plausible explanation is that these genes are duplicated in many snails, so reads from two (or more) paralogs are aligning to the same gene in the reference genome, falsely inflating apparent heterozygosity. At these genes, are both alleles observed as homozygous in different snails? Given this bizarrely inflated heterozygosity, I strongly suspect that an undetected duplication event has occurred in the fucolectin gene, the GRL101 gene, the P450 gene, and maybe other balancing selection candidates. This would also explain

the other apparent signs of balancing selection, such as β score and Tajima's D. Unless the authors have compelling evidence that that the variation they observe represents segregating alleles at a single locus, they should refrain from showcasing these genes as examples of balancing selection. If a large proportion of their balancing selection candidates look like this, they should re-interpret their overall conclusions about balancing selection. For example, they might choose to scan for balancing selection by only considering sites where all three genotypes are observed (two homozygotes and the heterozygote), to minimize this cryptic paralog issue.

Response: Thank you for your thorough review of our manuscript and your insightful comments regarding the balancing selection analysis. We fully understand your concerns about the unusually high heterozygosity observed in certain genes, potentially resulting from technical issues in analysis due to genes duplication in many snails. Following the reviewer's suggestion, in order to detect gene duplications in Pcan_SH genome, orthofinder software was performed to identify gene families and duplicated genes by using genomes of P. canaliculata, P. maculata and Marisa cornuarietis. Nevertheless, no evidence of gene duplication was discovered for the genes that were specifically highlighted in our manuscript including fucolectin (Pca0147430) and GRL101 (Pca0102750). Conversely, we did find a duplication event in the P450 gene (Pca0030410), but four of the five SNPs that highlighted in Figure S18d were found in all three genotypes. However, we fully agree with the reviewer that genome complexity and gene duplication would lead to the inaccurate identification of segregating alleles at a single locus. In our original manuscript, we have implemented stringent filtering criteria for SNP. To further validate our data analysis, we applied the SNPable procedures to mask regions of low-mappablity of Pcan_PH. Briefly, the de novo genome was divided into overlapping 75-mers and remapped onto the reference, keeping only the regions where most overlapping 75-mers were mapped uniquely and did not contain 1-mismatch hits. SNPs that did not located in these regions have been removed. The Mappability Masks step largely guaranteed only genome regions with a high enough mappability—that is, no repeat regions—are included. As a result, our SNPs for balancing selection scanning were likely absent from the repeated regions. In order to further reduce false positives, we calculated the proportion of uniquely mapped reads for each base of the genomes, genotypes were marked as missing if the proportion was below 80%. For balance selection, only SNPs with a MAF > 0.05 and missing call rate < 5% in each population were kept. With these procedures, the effects of gene duplication events may be mitigated and the accuracy of segregating alleles at a single locus can be guaranteed.
After these refinements, 5,968,777and 3,667,579 SNPs were retained in EA and SEA populations, respectively, for balancing selection analysis. Despite these stringent filtering criterions, our results consistently supported that the fucolectin, GRL101 and P450 were among the genes with balancing selection. There were 48 genes exhibiting balancing selection in both EA and SEA populations, more than 70% of these genes did not experience gene duplication events. With these extra analyses, we believe our results support the presence of balance selection in the highlighted genes. Thank you again for helping us improve our manuscript.

Lines 307-310. I found this sentence confusing. Even if balancing selection occurs on a large number of genes, it is implausible that it would occur on so many genes that it would meaningfully skew the genome-wide Tajima's D values. If genome-wide Tajima's D is positive, this should be interpreted in demographic terms, not in terms of balancing selection. Tajima's D can be positive due to a founder effect bottleneck or due to recent admixture between two or more lineages, and both of these scenarios are quite plausible for an invasive species. Besides, there is no reason to invoke global Tajima's D as a justification for a balancing selection scan, because scanning for balancing selection is a very reasonable thing to do with this dataset regardless.

Response: We thank the reviewer for the suggestion. We totally agree with the reviewer and following the suggestion, we have removed the affected sentences. Thank you for your insightful suggestion again.

The association between CSDE1 and cold temperature is compelling. But in Figure 3b, there are so many haplotypes shown that no meaningful interpretation is possible. Looking at Supp Figure S12, it seems you could simplify things by grouping adjacent haplotypes. For example, the group of Hap1, Hap5, Hap12, and Hap7 represents a clade and appears to be relatively common in LT. Figure 3b would be better if only 2 or 3 of such groups are shown.

Response: Thank you for your valuable suggestion to improve the interpretation of haplotypes in Figure 3b. We have grouped the adjacent haplotypes into two distinct clades, as illustrated in revised supplementary Figure S15. We hope that we provided a clear and more concise presentation of the haplotype distribution of CSDE1 in LT population, please see the revised Figure 3b.

The TreeMix analysis in Figure 2d is interesting, but there are so many populations that it is hard to interpret. Could they group populations into four or five groups based on the PCA and ADMIXTURE results (Figure 1)? Then it might be easier to see what is going on with TreeMix.

Response: We appreciate your insightful suggestion to simplify the interpretation of the TreeMix analysis in Figure 2d by grouping populations based on the PCA and ADMIXTURE results from Figure 1. According to your suggestion, we have carefully grouped samples from SEA and EA populations into five sub-populations, including ZJ, SH, EA_mix, EA_sole and SEA populations. Based on the proportions of genetic components inferred by ADMIXTURE results, we found that SH and ZJ samples shared their unique components, samples from SC, GDSZ, YNCJ, GZ, HB and JX locations presented both East Asian and Southeast Asian components (EA_mix), samples from HN, GX, JS FJ and GDZQ locations (EA_sole) were clustered together and barely shared genetic components with the SEA populations. We hope that this revised presentation aligns better with your expectations and facilitates a more straightforward interpretation of the data.

Response to the comments of Reviewer #2

Lu et al. investigated 173 genomes of P. canaliculata, representing 17 geographic populations, offering new insights into evolution and low-temperature adaptation. While the sequencing data has informative value, the manuscript requires significant reorganization due to several issues.

Specific points to address:
1. The concern lies in the author's exaggeration, notably in the title. The sampling encompasses only mainland China and parts of the Indochina Peninsula, neglecting crucial areas like Japan, South Korea, South Asia, and the Malay Peninsula, which are significant in the Asian distribution of applesnail. To enhance the manuscript's academic value, expanding the sample range extensively is recommended.

Response: Thank you for your suggestion. We fully understand the reviewer’s concern that the apple snails distribute in many Asian countries, such as Japan, Malaysia, Indonesia and Thailand. It would be perfect and valuable suppose we could be able to collect samples from all over Asia, as well as from Australia, Papua New Guinea and even samples from its very original place: South America. Actually, over the previous few years, we had made an effort to cover these sampling areas which was also suggested by the reviewer. However, when obtaining samples from other courtiers, we had to recognize that we were hampered by the challenging problems with international cooperation and potential biological security in invasive species. Fortunately, we were able to obtain a small number of samples from three countries in Southeast Asia. To our knowledge, our current study has been the only one that covered the most comprehensive geographical samples so far. In addition, our results showed that the latitude-varying sample distributions employed in this manuscript are applicable for investigating the expansion and adaptive evolutions and deriving the conclusions presented. We will surely make greater efforts to broaden the sample distribution according to the reviewer’s suggestions in our further study, we thank the reviewer who appreciate our efforts in making the best representative samples available in current study.

2. Exaggeration extends to the abstract, claiming a lack of whole-genome data on P. canaliculata's evolution and adaptation. However, considerable genomic and transcriptome data, including multiple genome versions and transcriptomes related to low-temperature stimulation, have been previously published.

Response: Thank you for your comments. We would clarify that it is the fact that the field is lack of the WHOLE-GENOME RE-SEQUNCING data on P. canaliculate, especially there have been NO any other POPULATION-LEVEL sequencing data available. We feel regret that our description resulted in misunderstanding of the reviewer. Accordingly, we have made revisions on the affected description. Please see the Abstract.

3. The introduction offers a biased or incomplete review of PC research, omitting vital information. For instance, the statement about PC's absence in the northern region contradicts a 2020 report by Qin et al. on a northern population's resistance to low temperatures. Mentioning numerous articles on low-temperature adaptation, covering genomics, transcriptomics, and gene families, is necessary.

Response: Thank you for the comment. However, it turned out that the reviewer apparently mis-interpreted the “the northern area” mentioned in Qin et al,2020 (Huanggang in Hubei, Central China) as “Northern China” in our manuscript, by saying “Northern China”, we generally refer to much more Northern than Hubei, as also a common sense in the literature and general readers. In fact, we carefully checked the publication mentioned by the reviewer. Qin et al,2020 sampled P. canaliculate populations from five latitude and longitude ranges of subtropical China: Guangzhou, Yingtan, Ningbo, Ya'an and Huanggang. Despite being in the northernmost subtropical zone, Huanggang is in central China. After carefully reviewing our manuscript, we discovered that the sentence "PC has established natural populations in most of southern China, but none in the northern area" was a reference to Yang QQ et al. (2018). Here, "northern area" refers to northern China which is much colder than the subtropical zone. In order to avoid misleading, we included the reference and added northern China. Following the reviewer’s suggestion, references on gene families, transcriptomics, and pathways related to low-temperature adaptation are included. Please see the line 36-44.

4. Accurate location and environmental information for the samples are crucial for genetic structure and low-temperature adaptation analysis, yet lacking in the manuscript.

Response: Thank you for your comments. We have included the information mentioned by the reviewer in Table S2.

5. The manuscript's reassembly of the PC genome raises questions about its necessity given the existence of previous genome versions. The author proposes that this is a genome from a female individual, and if compared to published versions, clarifying the improvements of this version will help enhance the value of this article. For example, improvements in assembly and gene prediction, as well as identification of sex chromosomes, etc

Response: Thank you for your comments and suggestions. We agreed that providing a detailed comparison and highlighting the improvements will enhance the value and clarity of our article. We have included a section in the revised manuscript to showcase the summary statistics and improvements in our newly assembled genome. Please see the line 65-92.
Comparing the previously published genome (Liu et al., 2018), our newly assembled genome exhibits comparable genome contiguity and completeness (Table S1). Taking advantage of the high coverage Hi-C data, we were able to observe that while several off-diagonal interactions were present in the previously published genome, none of them could be seen in our newly assembled Pcan SH genome, suggesting a more precise and accurate assembly process for Pcan_SH (Figure S2). It turned out that our new reference genome facilitated identifying several chromosomal rearrangements that have not been reported before with the older assembly (Figure S3).
Genome synteny analysis with MUMmer and SyRI software identified significant chromosomal rearrangements between the two genome assemblies, including a total of 95 inversion, 1242 translocations events, which can either result from misassembly or structural variations (Figure S4). These results suggest potential difference in genome structures or sequences between P. canaliculata from Shanghai city and Shenzhen city, underscoring the significance of our newly assembled genome as an indepentent representative genome for P. canaliculata.
Furthermore, we have paid special attention to identification of sex chromosomes, but we did not observe discernible differences in genome coverage for each chromosome among all re-sequenced samples, including female and male snails. Consistent to our observation, previous studies (e.g., Diupotex-Chong, 1994) have revealed that no sex chromosomes were identified in any Mexican species of Pomacea either. It suggests that sex determination in Pomacea still needs further research. Thank you once again for your constructive feedback.

6. Citing omics data from various sources without addressing potential biases from population differences,

sample processing methods, and sequencing techniques raises concerns about their comparability with the author's data.

Response: Thank you for your comments. Although we did not fully get the point of the reviewer, we agree that data from various sources could have batch effects. Therefore, we deliberately AVOID combing data from different resources. Instead, we analyze only genomic data from our own group. For genomic data, all DNA samples were taken from the same tissue using the same protocol and sequencing platform. To eliminate any potential biases or batch effects, all WGS data were mapped to a P. canaliculate reference genome and applied with a joint-calling procedure. For RNA-seq data, all raw reads were downloaded from the SRA (BioProject PRJNA427478) to avoid potential bias in omics data from other studies. To clarify, we must emphasize that population difference is per se the key question we aimed to study, it is exactly the scientific interest rather than a technical issue to be addressed. It is a common routine practice for population genomics analysis and has been used extensively in population genetics across a variety of species, including humans[1], animals[2], plants[3, 4] and pathogens[5]. We wish the reviewer could understand and support.

1. Ge X, Lu Y, Chen S, Gao Y, Ma L, Liu L, et al. Genetic Origins and Adaptive Evolution of the Deng People on the Tibetan Plateau. Mol Biol Evol. 2023;40 10 doi:10.1093/molbev/msad205.
2. Pecnerova P, Garcia-Erill G, Liu X, Nursyifa C, Waples RK, Santander CG, et al. High genetic diversity and low differentiation reflect the ecological versatility of the African leopard. Curr Biol. 2021;31 9:1862-71 e5. doi:10.1016/j.cub.2021.01.064.
3. Zeng L, Tu XL, Dai H, Han FM, Lu BS, Wang MS, et al. Whole genomes and transcriptomes reveal adaptation and domestication of pistachio. Genome Biol. 2019;20 1:79. doi:10.1186/s13059-019-1686-3.
4. Zhao H, Sun S, Ding Y, Wang Y, Yue X, Du X, et al. Analysis of 427 genomes reveals moso bamboo population structure and genetic basis of property traits. Nat Commun. 2021;12 1:5466. doi:10.1038/s41467-021-25795-x.
5. Luo F, Yang W, Yin M, Mo X, Pang Y, Sun C, et al. A chromosome-level genome of the human blood fluke Schistosoma japonicum identifies the genomic basis of host-switching. Cell Rep. 2022;39 1:110638. doi:10.1016/j.celrep.2022.110638.

7. In the PCA analysis, distinguishing EA and SEA samples using different colors proves ineffective due to clustered points, particularly between these regions.

Response: We appreciated your feedback regarding the challenges in distinguishing EA and SEA samples using different colors. To address the issue, we utilized different shapes for EA and SEA samples allowing for a clearer distinction. Please see the revised Figure 1b.

8. The structural diagram presents confusion, especially regarding JS, SH, and ZJ samples due to their proximity in geographical location. The cause behind this result is unclear, attributed by the author to human factors without substantial evidence. It cannot be ruled out that the error was caused during the sampling process.

Response: Thank the reviewer for the comments. However, the reviewer seemly misunderstood our description in the manuscript. We did not attribute the genetic structure to human factors. Instead, we interpreted the results as “the possibility of multiple invasions of Asia by showing varying degrees of migration and genetic interactions”. Please see the line 137-140.

9. Extensive discussions on screened genes in the manuscript lack substantial evidence for their functions. If inferred based on sequence similarity, providing similarity values between screened and verified functional genes is essential. The expression data of transcriptome and rt PCR cannot confirm the function of the gene, which greatly increases the confusion of the conclusion.

Response: Thank you for your comments. Although we did not fully get the point of the reviewer, we do not believe it makes much sense “providing similarity values between screened and verified functional genes”. The “verified functional genes” are already in the database or literature, but any genes can have sequence variations, a value to measure similarity of a gene sequence observed in a studied sample and the gene in the database does not provide any informative message for the gene function. Without functional experiments, one cannot get any clue about the functionality of the gene version observed in the

studied sample. Actually, RT-PCR and expression data can provide very helpful results for understanding the functionality of the gene variations. Of course, biology is complex, any one single study of limited number of genes is not expected to provide a full picture of the functional mechanism, because many genes can be involved, thousands of studies of hundreds of genes with a huge number of different assays are necessary to eventually understand the function of even a single gene. Therefore, we agree that further study is needed to investigate genes that are potentially interesting or functionally important.

10. The writing of the article was careless. There are many grammar and spelling problems. Including but not limited to, the Latin names of species in the introduction were not changed to abbreviations during the second appearance.

Response: We thank the reviewer for the comments. Although we agree that the Latin names of species in the introduction were not changed to abbreviations during the second appearance, we did not identify MANY grammar and spelling problems, in fact, we have carefully checked our text before submission, we also used a commercial service of language editing to improve our writing. However, we would appreciate if the reviewer could point detailed grammar and spelling problems so that we are able to further improve the manuscript text. Thank you again.
