# Supplementary material for: Whole-genome sequencing of the invasive golden apple snail Pomacea canaliculata from Asia reveals rapid expansion and adaptive evolution
Source: Gigascience. 2024 Sep 23;13:giae064. doi: 10.1093/gigascience/giae064 (PMC11417965; doi:10.1093/gigascience/giae064)
Supplement: giae064_Response_to_Reviewer_Comments_Revision_1 [file giae064_response_to_reviewer_comments_revision_1.pdf]

Author's Response To Reviewer Comments

Point-to-point response to the reviewers' comments

Response to the comments of Reviewer #1
Reviewer #1: The authors have made admirable improvements to the manuscript, in particular the comparisons between Pcan_SH and Pcan_SZ. However, there is still a fatal flaw in the balancing selection analysis. The authors state that an orthofinder search was employed to find duplicated genes. But this does not address the issue I raised earlier. My concern is that there are hidden paralogs that have not been assembled into to genome, which would probably be missed by orthofinder, especially if the duplications are recent and unique to this species. The fact remains that Figure 5e is is just biologically impossible, and this will be readily apparent to any reader with a basic understanding of population genetics. You simply can't have a set of biallelic SNPs with heterozygosity of 1 in a large number of diploid samples. And Figure 5e is just the gene they happen to highlight; they report a similar pattern in several genes. The only explanation is misaligned reads. Their balancing selection analysis is confounded by these artifactual genotypes. It is unacceptable to publish this balancing selection analysis without first correcting or excluding these artifacts. The authors took steps to remove SNPs in low-mappability regions or with lots of missing data, which is fine, but that doesn't get at the main problem. Hardy-Weinberg filtering is required. Even a very stringent Hardy-Weinberg filter, allowing for minor deviations due to population structure and selection, would still remove the egregious cases of SNPs showing heterozygosity > 0.8 among over 100 samples. I cannot approve the manuscript as is.
Response: Thank you very much for your insightful suggestion and instruction. We agreed that we could not fully exclude the possibility of the hidden paralogs that have not been assembled into to genome, indeed, which could probably be missed by orthofinder. Accordingly, we carefully reconsidered this issue and agree with you that the SNPs filtering based on the unique mapping ratio indeed cannot solve the problem. In particular, suppose the duplications are recent and the genome duplication occurred, we might have missed assembly of these regions accurately. A set of biallelic SNPs with heterozygosity of 1 might be the result of misaligned reads.
Therefore, we applied some more stringent criteria to address this problem. In particular, we treated the genotypes as missing data when the proportion of reads that uniquely mapped was below 80%. Furthermore, following the method recommended by Heng Li (https://github.com/lh3/psmc), we retained the genotype only when the depth of variants was at least one-third of the average read depth and no more than two-fold of the average read depth. By applying the sequence depth, we can filter out the SNPs located in duplicated regions if their sequence depth is greater than two-fold of the average read depth. Similarly, we can filter out the SNPs located in depletion regions if their sequence depth is less than one-third of the sample average depth. We also have incorporated these updated methods into our manuscript with the aforementioned information. Eventually, we have carefully examined the results the SNPs showing strong balancing selection do not exhibit extremely high observed heterozygosity, as the reviewer anticipated. In particular, 99.9% of the SNPs in our reported data are in heterozygosity < 0.75, which we believe are much closer to the expectation as the reviewer mentioned. The candidate genes list has partially changed and we have updated the results section (the lines 377-405) as well as the Table S9-S11. We appreciate your persistence in pointing out this issue. We hope that these refinements address the concerns raised regarding the balancing selection analysis. Thank you again for helping us improve our manuscript.

Response to the comments of Reviewer #2
Reviewer #2: The authors have made new analysis and answered the questions, although the questions about sample information and gene function are in my opinion not perfect.
Response: Thank you again for your feedback on our manuscript. We appreciate your acknowledgment of the improvements we've made in response to your suggestions. We have carefully considered your comments regarding the sample information and gene function sections, and we are committed to making further improvements based on your feedback to ensure the manuscript meets the highest standards.

The environmental information of the sample is crucial for population research. Not similar to other naturally distributed species, the apple snail was considered an economic species for a long time and was

artificially transported and spread. Simultaneously, through online transactions and express delivery networks, this dissemination has been accelerated. Therefore, discovering a snail at a location does not necessarily mean that it has existed here for a long time and has adapted to the environment. It is very important to determine whether the snails in the sampling area are stable and colonized populations. The author accurately listed the longitude and latitude of the sample locations, but after verification, it was found that these locations were located by the municipal governments of each city. So, the table in the answer is manipulated.
Response: We apologize for the sample's geographical location issue. We carefully checked the records during the sample collection and now we can clarify that all samples were collected in rivers, rice fields, and ponds, NOT in Municipal Government buildings which were incorrectly marked by one of the authors who was not aware of the difference between the exact sample location and the rough place representing a province or a city. The reason was that the author assumed the coordinates for a sample were just a label to reflect the information of a province or a city because our comparative analyses were at a scale of a province or a city. Please find the revised table S2.
In addition, the sample location information was obtained from the original record in the National Parasitic Resource Center (https://www.tdrc.org.cn/, Chen et al., Establishment and application of the National Parasitic Resource Center (NPRC) in China, Adv Parasitol. 2020:110:373-400), which is a first-level platform under the Basic Condition Platform Center of the Ministry of Science and Technology of China. We just took the ORIGINAL sample information from NPRC (www.portal.tdrc.org.cn). The samples in one site were selected from close or surrounding places to ensure the temperature remained consistent within a certain range. It is a routine sample collection strategy not only in population genomics analysis to investigate the genetic variation at a population level rather than the individual level but also in the P. canaliculata populations study in responses to cold acclimation (Qin Z et al., Survivorship of geographic P. canaliculata populations in responses to cold acclimation. Ecol Evol. 2020;10 8:3715-26). We hope the revised table S2 with the location information will address the reviewer’s concern.

Unfortunately, this sampling location information does not help explain the samples in some areas, making them appear more suspicious. For example, JS, SH, and ZJ are geographically very close, but in the structure diagram, they display almost completely different patterns.
Response: Thank you for your comments. Through population genetics methods, we found no significant positive correlation between the genetic distance and geographic distance of P. canaliculata in China. This finding is consistent with the results of our PCA and structure analysis. It is an important result of our study that the spread of P. canaliculata within China is still largely influenced by human activities, resulting in extensive gene flow events.
Specifically, although JS, SH, and ZJ are geographically very close, they exhibit almost completely different patterns in the structure diagram. This further supports our conclusion. Previous literature has documented multiple introduction and invasion events of P. canaliculata in China, explaining why the genetic distances between JS, SH, and ZJ populations are vast despite their geographic proximity. These populations likely originated from different introduction and invasion events, leading to significant genetic differences.
In summary, we believe these results not only reflect the complex history of the spread and dispersion of P. canaliculata but also highlight the critical role of human activities in its distribution.

Another issue is gene function. We have noticed that the author did not use the genome and genes from NCBI Refseq. But rather reassembled and annotated a new version of the genome. In the method of functional prediction, the author mentions "using local BLASTX and BLASTN programs with an e-value of 1e-5". Using only e-value without filtering identity and coverage, these results are very rough. Partial alignments can easily lead to incorrect annotation results. For the genes screened by the author, their functions need further confirmation and more scientific methods should be used. Quantity RT-PCR mainly verifies the FPKM/TPM of the transcriptome. And it hardly helps with confirming the functionality.
Response: Thank you for your comments. We appreciate your attention to detail and your valuable feedback regarding our approach to gene function prediction and validation.
In fact, we used the re-assembled genome and gene annotation that sampled from the Shanghai city for our population analysis. Through rigorous genome quality assessment methods, we established the suitability of this genome and annotation for our population genomics study. Given that genomic resources for mollusks are relatively sparse, Pcan_SH contributes to the broader set of published mollusk genomes. Furthermore, the observed chromosomal structural variations between the Pcan_SH and Pcan_SZ genomes underscore the significance of utilizing a new reference genome for our research.

Regarding functional prediction method employing local BLASTX and BLASTN programs with an e-value threshold of 1e-5, we acknowledge your concerns about relying solely on e-values without considering identity and coverage thresholds. However, it's worth noting that this parameter is commonly used in various research studies for gene annotation, as demonstrated in recent publications such as "Chromosome-level genome assembly and population genomic resource to accelerate orphan crop lablab breeding" (Nature Communications, 2023) and "A chromosome-level genome assembly of radish (Raphanus sativus L.) reveals insights into genome adaptation and differential bolting regulation" (Plant Biotechnology Journal, 2023). We believe this parameter is appropriate for gene annotation, due to its widespread acceptance in the scientific community. In our original manuscript, we incorporated additional methods for gene functional annotation to address the limitations of relying solely on e-values from BLAST software, although we neglected to include this information in our original method section. We employed InterProScan for protein domain annotation, and Kyoto Encyclopedia of Genes and Genomes (KEGG) pathway and Gene Ontology (GO) annotations were updated using the eggNOG v5.0 database with specific parameters ("--evalue 0.001 --score 60 --pident 40 --query_cover 20 --subject_cover 20"). These additional methods provide complementary information and enhance the robustness of our gene function predictions.

For the genes we highlighted by positive selection or balancing selection methods, we conducted thorough gene annotation validation by integrating multiple software tools such as BLAST, eggNOG, and InterProScan. For instance, for the gene CSDE1, we identified high sequence identity with known CSDE1 proteins in databases such as SwissProt and eggNOG, as well as the presence of a 'Cold-shock' DNA-binding domain predicted by InterProScan. Furthermore, quantitative RT-PCR confirmed a significant increase in CSDE1 expression in response to cold exposure, supporting its potential role in the cold-shock response. These findings provide valuable insights into the potential function of CSDE1 in cold response.

Actually, both CRISPR-Cas9 and RNAi are reliable experimental approaches for examining and validating the gene functions. None of these techniques, however, can be used successfully on the apple snails so far. In this work, we identified a set of putative genes with the goal of offering hints regarding their potential functions for future biological investigations, as well as to provide a valuable resource concerning the invasive species.

Thank you once again for your valuable input and we look forward to incorporating your suggestions to improve our manuscript.
