# Supplementary material for: Whole-genome sequencing of the invasive golden apple snail Pomacea canaliculata from Asia reveals rapid expansion and adaptive evolution
Source: Gigascience. 2024 Sep 23;13:giae064. doi: 10.1093/gigascience/giae064 (PMC11417965; doi:10.1093/gigascience/giae064)
Supplement: giae064_Reviewer_1_Report_Original_Submission — Jacob Tennessen -- 10/30/2023 Reviewed [file giae064_reviewer_1_report_original_submission.pdf]

Reviewer Report

Title: Whole-genome sequencing of the invasive golden apple snail Pomacea canaliculata from Asia reveals rapid expansion and adaptive evolution

Version: Original Submission **Date:** 10/30/2023

Reviewer name: Jacob Tennessen

Reviewer Comments to Author:

This is a remarkably thorough paper that combines whole genome assembly and annotation, population genomics, and gene expression data for the golden apple snail Pomacea canaliculata. The authors identify signals of demographic history and natural selection resulting from this invasive species' spread across Asia. They pinpoint specific genes adapted to temperature and other stressors that these snail populations have encountered. Overall, this is an impressive and sweeping genome-scale study on a poorly-known non-model species.

The authors assemble and annotate a genome for this snail species. However, the genome of this species was already published several years ago in Gigascience (Liu et al. 2018), and it is a high-quality genome featuring chromosome-level assembly. So it was not evident why they generated a new genome. It is quite plausible that their new genome is better than the published genome in various respects, but in the manuscript there is no mention of any kind of comparison between the new and old genome. While they do cite the previous genome paper (reference 25), it is only in the context of RNA-Seq data, so there is no indication in the manuscript that a published genome even exists. A brief justification and comparison is warranted. When they mention a "reference genome" (e.g. line 61), I am assuming they mean their new genome not the old one, but this should be clarified. Furthermore, given that genomic resources for mollusks are relatively sparce (see Davison & Neiman 2021), it wouldn't hurt to have a sentence or two on how this new assembly contributes to the broader set of published mollusk genomes.

The selection scans are well executed and generally robust. However, I had a few concerns about their balancing selection analysis. While it is true that a mild excess of heterozygosity surpassing Hardy-Weinberg expectations is a telltale sign of balancing selection, it is virtually unheard of in any species that selection coefficients would be so strong as to push heterozygosity for a biallelic polymorphism to 80-100%, as in Figure 5e, Figure S17d, and Figure S18d. To have this effect at several different unlinked genes in the same populations is basically impossible from a genetic load perspective. A much more plausible explanation is that these genes are duplicated in many snails, so reads from two (or more) paralogs are aligning to the same gene in the reference genome, falsely inflating apparent heterozygosity. At these genes, are both alleles observed as homozygous in different snails? Given this bizarrely inflated heterozygosity, I strongly suspect that an undetected duplication event has occurred in the fucolectin gene, the GRL101 gene, the P450 gene, and maybe other balancing selection candidates. This would also explain the other apparent signs of balancing selection, such as Î² score and Tajima's D. Unless the authors have compelling evidence that that the variation they observe represents segregating alleles at a single locus, they should refrain from showcasing these genes as examples of

balancing selection. If a large proportion of their balancing selection candidates look like this, they should re-interpret their overall conclusions about balancing selection. For example, they might choose to scan for balancing selection by only considering sites where all three genotypes are observed (two homozygotes and the heterozygote), to minimize this cryptic paralog issue.

Lines 307-310. I found this sentence confusing. Even if balancing selection occurs on a large number of genes, it is implausible that it would occur on so many genes that it would meaningfully skew the genome-wide Tajima's D values. If genome-wide Tajima's D is positive, this should be interpreted in demographic terms, not in terms of balancing selection. Tajima's D can be positive due to a founder effect bottleneck or due to recent admixture between two or more lineages, and both of these scenarios are quite plausible for an invasive species. Besides, there is no reason to invoke global Tajima's D as a justification for a balancing selection scan, because scanning for balancing selection is a very reasonable thing to do with this dataset regardless.

The association between CSDE1 and cold temperature is compelling. But in Figure 3b, there are so many haplotypes shown that no meaningful interpretation is possible. Looking at Supp Figure S12, it seems you could simplify things by grouping adjacent haplotypes. For example, the group of Hap1, Hap5, Hap12, and Hap7 represents a clade and appears to be relatively common in LT. Figure 3b would be better if only 2 or 3 of such groups are shown.

The TreeMix analysis in Figure 2d is interesting, but there are so many populations that it is hard to interpret. Could they group populations into four or five groups based on the PCA and ADMIXTURE results (Figure 1)? Then it might be easier to see what is going on with TreeMix.

Methods

Are the methods appropriate to the aims of the study, are they well described, and are necessary controls included? Choose an item.

Conclusions

Are the conclusions adequately supported by the data shown? Choose an item.

Reporting Standards

Does the manuscript adhere to the journal's guidelines on minimum standards of reporting? Choose an item.

Choose an item.

Statistics

Are you able to assess all statistics in the manuscript, including the appropriateness of statistical tests used? Choose an item.

Quality of Written English

Please indicate the quality of language in the manuscript: Choose an item.

Declaration of Competing Interests

Please complete a declaration of competing interests, considering the following questions:

- Have you in the past five years received reimbursements, fees, funding, or salary from an organisation that may in any way gain or lose financially from the publication of this manuscript, either now or in the future?
- Do you hold any stocks or shares in an organisation that may in any way gain or lose financially from the publication of this manuscript, either now or in the future?
- Do you hold or are you currently applying for any patents relating to the content of the manuscript?
- Have you received reimbursements, fees, funding, or salary from an organization that holds or has applied for patents relating to the content of the manuscript?
- Do you have any other financial competing interests?
- Do you have any non-financial competing interests in relation to this paper?

If you can answer no to all of the above, write 'I declare that I have no competing interests' below. If your reply is yes to any, please give details below.

I declare that I have no competing interests

I agree to the open peer review policy of the journal. I understand that my name will be included on my report to the authors and, if the manuscript is accepted for publication, my named report including any attachments I upload will be posted on the website along with the authors' responses. I agree for my report to be made available under an Open Access Creative Commons CC-BY license (http://creativecommons.org/licenses/by/4.0/). I understand that any comments which I do not wish to be included in my named report can be included as confidential comments to the editors, which will not be published.

Choose an item.

To further support our reviewers, we have joined with Publons, where you can gain additional credit to further highlight your hard work (see: https://publons.com/journal/530/gigascience). On publication of this paper, your review will be automatically added to Publons, you can then choose whether or not to claim your Publons credit. I understand this statement.

Yes Choose an item.
