# Supplementary material for: Whole-genome sequencing of the invasive golden apple snail Pomacea canaliculata from Asia reveals rapid expansion and adaptive evolution
Source: Gigascience. 2024 Sep 23;13:giae064. doi: 10.1093/gigascience/giae064 (PMC11417965; doi:10.1093/gigascience/giae064)
Supplement: giae064_Reviewer_1_Report_Revision_1 — Jacob Tennessen -- 2/19/2024 Reviewed [file giae064_reviewer_1_report_revision_1.pdf]

Reviewer Report

Title: Whole-genome sequencing of the invasive golden apple snail Pomacea canaliculata from Asia reveals rapid expansion and adaptive evolution

Version: Revision 1 **Date:** 2/19/2024

Reviewer name: Jacob Tennessen

Reviewer Comments to Author:

The authors have made admirable improvements to the manuscript, in particular the comparisons between Pcan_SH and Pcan_SZ. However, there is still a fatal flaw in the balancing selection analysis. The authors state that an orthofinder search was employed to find duplicated genes. But this does not address the issue I raised earlier. My concern is that there are hidden paralogs that have not been assembled into to genome, which would probably be missed by orthofinder, especially if the duplications are recent and unique to this species. The fact remains that Figure 5e is is just biologically impossible, and this will be readily apparent to any reader with a basic understanding of population genetics. You simply can't have a set of biallelic SNPs with heterozygosity of 1 in a large number of diploid samples. And Figure 5e is just the gene they happen to highlight; they report a similar pattern in several genes. The only explanation is misaligned reads. Their balancing selection analysis is confounded by these artifactual genotypes. It is unacceptable to publish this balancing selection analysis without first correcting or excluding these artifacts. The authors took steps to remove SNPs in low-mappability regions or with lots of missing data, which is fine, but that doesn't get at the main problem. Hardy-Weinberg filtering is required. Even a very stringent Hardy-Weinberg filter, allowing for minor deviations due to population structure and selection, would still remove the egregious cases of SNPs showing heterozygosity > 0.8 among over 100 samples. I cannot approve the manuscript as is.

Methods

Are the methods appropriate to the aims of the study, are they well described, and are necessary controls included? Choose an item.

Conclusions

Are the conclusions adequately supported by the data shown? Choose an item.

Reporting Standards

Does the manuscript adhere to the journal’s guidelines on minimum standards of reporting? Choose an item.

Choose an item.

Statistics

Are you able to assess all statistics in the manuscript, including the appropriateness of statistical tests used? Choose an item.

Quality of Written English

Please indicate the quality of language in the manuscript: Choose an item.

Declaration of Competing Interests

Please complete a declaration of competing interests, considering the following questions:

- Have you in the past five years received reimbursements, fees, funding, or salary from an organisation that may in any way gain or lose financially from the publication of this manuscript, either now or in the future?
- Do you hold any stocks or shares in an organisation that may in any way gain or lose financially from the publication of this manuscript, either now or in the future?
- Do you hold or are you currently applying for any patents relating to the content of the manuscript?
- Have you received reimbursements, fees, funding, or salary from an organization that holds or has applied for patents relating to the content of the manuscript?
- Do you have any other financial competing interests?
- Do you have any non-financial competing interests in relation to this paper?

If you can answer no to all of the above, write 'I declare that I have no competing interests' below. If your reply is yes to any, please give details below.

I declare that I have no competing interests

I agree to the open peer review policy of the journal. I understand that my name will be included on my report to the authors and, if the manuscript is accepted for publication, my named report including any attachments I upload will be posted on the website along with the authors' responses. I agree for my report to be made available under an Open Access Creative Commons CC-BY license (http://creativecommons.org/licenses/by/4.0/). I understand that any comments which I do not wish to be included in my named report can be included as confidential comments to the editors, which will not be published.

Choose an item.

To further support our reviewers, we have joined with Publons, where you can gain additional credit to further highlight your hard work (see: https://publons.com/journal/530/gigascience). On publication of this paper, your review will be automatically added to Publons, you can then choose whether or not to claim your Publons credit. I understand this statement.

Yes Choose an item.
