# Supplementary material for: Whole-genome sequencing of the invasive golden apple snail Pomacea canaliculata from Asia reveals rapid expansion and adaptive evolution
Source: Gigascience. 2024 Sep 23;13:giae064. doi: 10.1093/gigascience/giae064 (PMC11417965; doi:10.1093/gigascience/giae064)
Supplement: giae064_Reviewer_2_Report_Original_Submission — Conghui Liu -- 11/13/2023 Reviewed [file giae064_reviewer_2_report_original_submission.pdf]

Reviewer Report

Title: Whole-genome sequencing of the invasive golden apple snail Pomacea canaliculata from Asia reveals rapid expansion and adaptive evolution

Version: Original Submission **Date:** 11/13/2023

Reviewer name: Conghui Liu

Reviewer Comments to Author:

Lu et al. investigated 173 genomes of P. canaliculata, representing 17 geographic populations, offering new insights into evolution and low-temperature adaptation. While the sequencing data has informative value, the manuscript requires significant reorganization due to several issues.
Specific points to address:
1. The concern lies in the author's exaggeration, notably in the title. The sampling encompasses only mainland China and parts of the Indochina Peninsula, neglecting crucial areas like Japan, South Korea, South Asia, and the Malay Peninsula, which are significant in the Asian distribution of applesnail. To enhance the manuscript's academic value, expanding the sample range extensively is recommended.
2. Exaggeration extends to the abstract, claiming a lack of whole-genome data on P. canaliculata's evolution and adaptation. However, considerable genomic and transcriptome data, including multiple genome versions and transcriptomes related to low-temperature stimulation, have been previously published.
3. The introduction offers a biased or incomplete review of PC research, omitting vital information. For instance, the statement about PC's absence in the northern region contradicts a 2020 report by Qin et al. on a northern population's resistance to low temperatures. Mentioning numerous articles on low-temperature adaptation, covering genomics, transcriptomics, and gene families, is necessary.
4. Accurate location and environmental information for the samples are crucial for genetic structure and low-temperature adaptation analysis, yet lacking in the manuscript.
5. The manuscript's reassembly of the PC genome raises questions about its necessity given the existence of previous genome versions. The author proposes that this is a genome from a female individual, and if compared to published versions, clarifying the improvements of this version will help enhance the value of this article. For example, improvements in assembly and gene prediction, as well as identification of sex chromosomes, etc
6. Citing omics data from various sources without addressing potential biases from population differences, sample processing methods, and sequencing techniques raises concerns about their comparability with the author's data.
7. In the PCA analysis, distinguishing EA and SEA samples using different colors proves ineffective due to clustered points, particularly between these regions.
8. The structural diagram presents confusion, especially regarding JS, SH, and ZJ samples due to their proximity in geographical location. The cause behind this result is unclear, attributed by the author to human factors without substantial evidence. It cannot be ruled out that the error was caused during the

sampling process.
9. Extensive discussions on screened genes in the manuscript lack substantial evidence for their functions. If inferred based on sequence similarity, providing similarity values between screened and verified functional genes is essential. The expression data of transcriptome and rt PCR cannot confirm the function of the gene, which greatly increases the confusion of the conclusion.
10. The writing of the article was careless. There are many grammar and spelling problems. Including but not limited to, the Latin names of species in the introduction were not changed to abbreviations during the second appearance

Methods

Are the methods appropriate to the aims of the study, are they well described, and are necessary controls included? Choose an item.

Conclusions

Are the conclusions adequately supported by the data shown? Choose an item.

Reporting Standards

Does the manuscript adhere to the journal's guidelines on minimum standards of reporting? Choose an item.

Choose an item.

Statistics

Are you able to assess all statistics in the manuscript, including the appropriateness of statistical tests used? Choose an item.

Quality of Written English

Please indicate the quality of language in the manuscript: Choose an item.

Declaration of Competing Interests

Please complete a declaration of competing interests, considering the following questions:

- Have you in the past five years received reimbursements, fees, funding, or salary from an organisation that may in any way gain or lose financially from the publication of this manuscript, either now or in the future?
- Do you hold any stocks or shares in an organisation that may in any way gain or lose financially from the publication of this manuscript, either now or in the future?

- Do you hold or are you currently applying for any patents relating to the content of the manuscript?
- Have you received reimbursements, fees, funding, or salary from an organization that holds or has applied for patents relating to the content of the manuscript?
- Do you have any other financial competing interests?
- Do you have any non-financial competing interests in relation to this paper?

If you can answer no to all of the above, write 'I declare that I have no competing interests' below. If your reply is yes to any, please give details below.

I declare that I have no competing interests

I agree to the open peer review policy of the journal. I understand that my name will be included on my report to the authors and, if the manuscript is accepted for publication, my named report including any attachments I upload will be posted on the website along with the authors' responses. I agree for my report to be made available under an Open Access Creative Commons CC-BY license (http://creativecommons.org/licenses/by/4.0/). I understand that any comments which I do not wish to be included in my named report can be included as confidential comments to the editors, which will not be published.

Choose an item.

To further support our reviewers, we have joined with Publons, where you can gain additional credit to further highlight your hard work (see: https://publons.com/journal/530/gigascience). On publication of this paper, your review will be automatically added to Publons, you can then choose whether or not to claim your Publons credit. I understand this statement.

Yes Choose an item.
