# Supplementary material for: Whole-genome sequencing of the invasive golden apple snail Pomacea canaliculata from Asia reveals rapid expansion and adaptive evolution
Source: Gigascience. 2024 Sep 23;13:giae064. doi: 10.1093/gigascience/giae064 (PMC11417965; doi:10.1093/gigascience/giae064)
Supplement: giae064_Reviewer_2_Report_Revision_1 — Conghui Liu -- 2/26/2024 Reviewed [file giae064_reviewer_2_report_revision_1.pdf]

Reviewer Report

Title: Whole-genome sequencing of the invasive golden apple snail Pomacea canaliculata from Asia reveals rapid expansion and adaptive evolution

Version: Revision 1 **Date:** 2/26/2024

Reviewer name: Conghui Liu

Reviewer Comments to Author:

The authors have made new analysis and answered the questions, although the questions about sample information and gene function are in my opinion not perfect.
The environmental information of the sample is crucial for population research. Not similar to other naturally distributed species, the apple snail was considered an economic species for a long time and was artificially transported and spread. Simultaneously, through online transactions and express delivery networks, this dissemination has been accelerated. Therefore, discovering a snail at a location does not necessarily mean that it has existed here for a long time and has adapted to the environment. It is very important to determine whether the snails in the sampling area are stable and colonized populations. The author accurately listed the longitude and latitude of the sample locations, but after verification, it was found that these locations were located by the municipal governments of each city. So, the table in the answer is manipulated.
Unfortunately, this sampling location information does not help explain the samples in some areas, making them appear more suspicious. For example, JS, SH, and ZJ are geographically very close, but in the structure diagram, they display almost completely different patterns.
Another issue is gene function. We have noticed that the author did not use the genome and genes from NCBI Refseq. But rather reassembled and annotated a new version of the genome. In the method of functional prediction, the author mentions "using local BLASTX and BLASTN programs with an e-value of 1e-5". Using only e-value without filtering identity and coverage, these results are very rough. Partial alignments can easily lead to incorrect annotation results. For the genes screened by the author, their functions need further confirmation and more scientific methods should be used. Quantity RT-PCR mainly verifies the FPKM/TPM of the transcriptome. And it hardly helps with confirming the functionality.

Methods

Are the methods appropriate to the aims of the study, are they well described, and are necessary controls included? Choose an item.

Conclusions

Are the conclusions adequately supported by the data shown? Choose an item.

Reporting Standards

Does the manuscript adhere to the journal's guidelines on minimum standards of reporting? Choose an item.

Choose an item.

Statistics

Are you able to assess all statistics in the manuscript, including the appropriateness of statistical tests used? Choose an item.

Quality of Written English

Please indicate the quality of language in the manuscript: Choose an item.

Declaration of Competing Interests

Please complete a declaration of competing interests, considering the following questions:

- Have you in the past five years received reimbursements, fees, funding, or salary from an organisation that may in any way gain or lose financially from the publication of this manuscript, either now or in the future?
- Do you hold any stocks or shares in an organisation that may in any way gain or lose financially from the publication of this manuscript, either now or in the future?
- Do you hold or are you currently applying for any patents relating to the content of the manuscript?
- Have you received reimbursements, fees, funding, or salary from an organization that holds or has applied for patents relating to the content of the manuscript?
- Do you have any other financial competing interests?
- Do you have any non-financial competing interests in relation to this paper?

If you can answer no to all of the above, write 'I declare that I have no competing interests' below. If your reply is yes to any, please give details below.

I declare that I have no competing interests' below

I agree to the open peer review policy of the journal. I understand that my name will be included on my report to the authors and, if the manuscript is accepted for publication, my named report including any attachments I upload will be posted on the website along with the authors' responses. I agree for my report to be made available under an Open Access Creative Commons CC-BY license (http://creativecommons.org/licenses/by/4.0/). I understand that any comments which I do not wish to be included in my named report can be included as confidential comments to the editors, which will not be published.

Choose an item.

To further support our reviewers, we have joined with Publons, where you can gain additional credit to further highlight your hard work (see: https://publons.com/journal/530/gigascience). On publication of this paper, your review will be automatically added to Publons, you can then choose whether or not to claim your Publons credit. I understand this statement.

Yes Choose an item.
